# Supplementary material for: Identification of 5-HT2A receptor signaling pathways associated with psychedelic potential
Source: Nat Commun. 2023 Dec 15;14:8221. doi: 10.1038/s41467-023-44016-1 (PMC10724237; doi:10.1038/s41467-023-44016-1)
Supplement: Supplementary file 3 — Description of Additional Supplementary Files [file 41467_2023_44016_MOESM3_ESM.pdf]

Description of Additional Supplementary items

File name: Supplementary file 1
Description: Contains ^{1}H, ^{13}C, and ^{19}F NMR chemical shift assignments for synthesized compounds

File name: Supplementary file 2
Description: Contains ^{1}H, ^{13}C, and ^{19}F NMR spectra for synthesized compounds

File name: Supplementary file 3
Description: HPLC traces for synthesized compounds

File name: Supplementary file 4
Description: Initial coordinate and simulation input file for 25CN-NBOH MD simulation

File name: Supplementary file 5
Description: Final coordinate and simulation output file for 25CN-NBOH MD simulation

File name: Supplementary file 6
Description: Initial coordinate and simulation input file for 25N-N1-Nap (16) MD simulation

File name: Supplementary file 7
Description: Final coordinate and simulation output file for 25N-N1-Nap (16) MD simulation
