# Supplementary material for: Identification of 5-HT2A receptor signaling pathways associated with psychedelic potential
Source: Nat Commun. 2023 Dec 15;14:8221. doi: 10.1038/s41467-023-44016-1 (PMC10724237; doi:10.1038/s41467-023-44016-1)

^{1}H NMR Spectra for Synthesized *N*-benzyl-phenethylamines.

Spectra 1. ^{1}H NMR of 25N-NB (2) HCl.

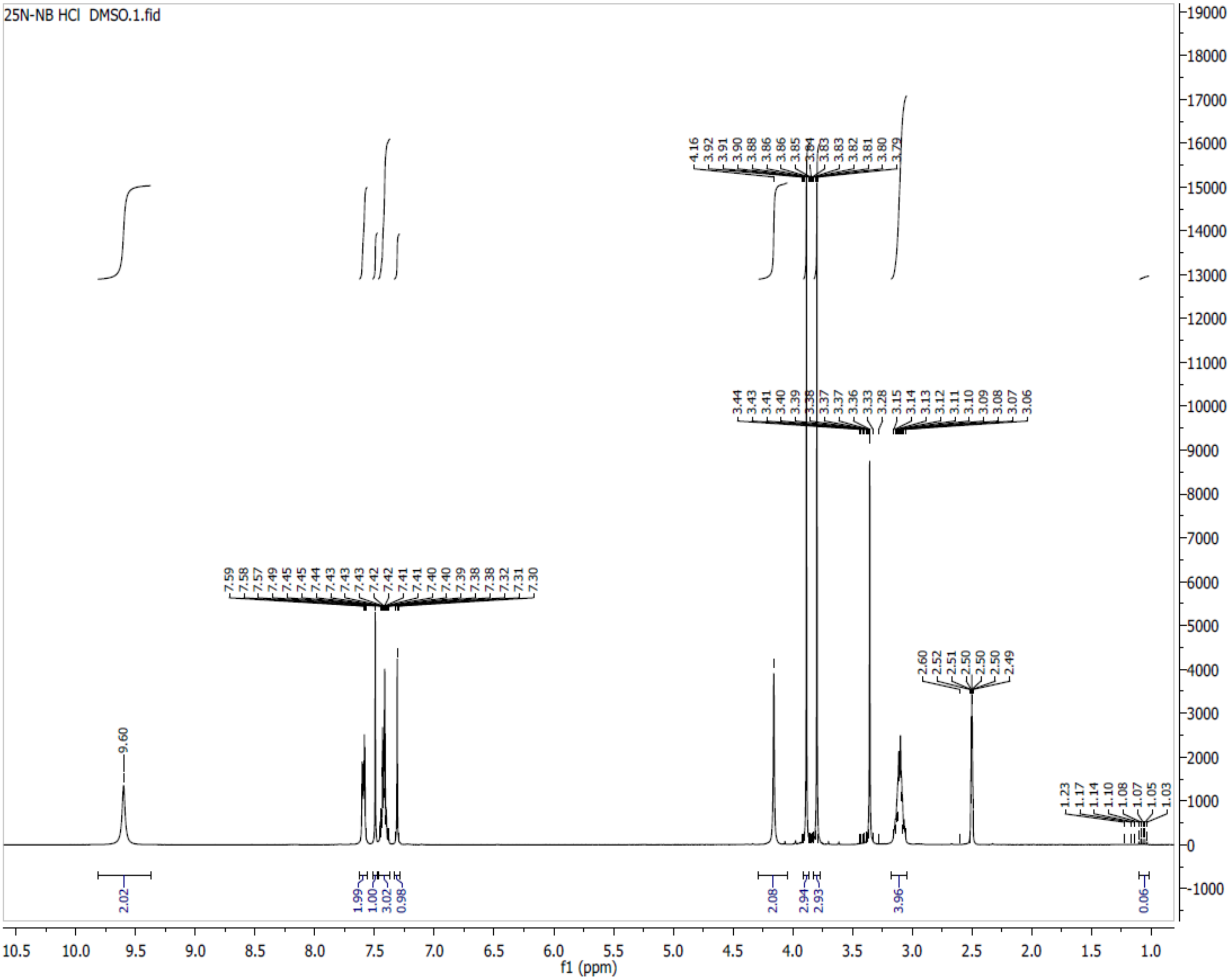

Spectra 2. ^{1}H NMR of 25N-NBOH (3) HCl

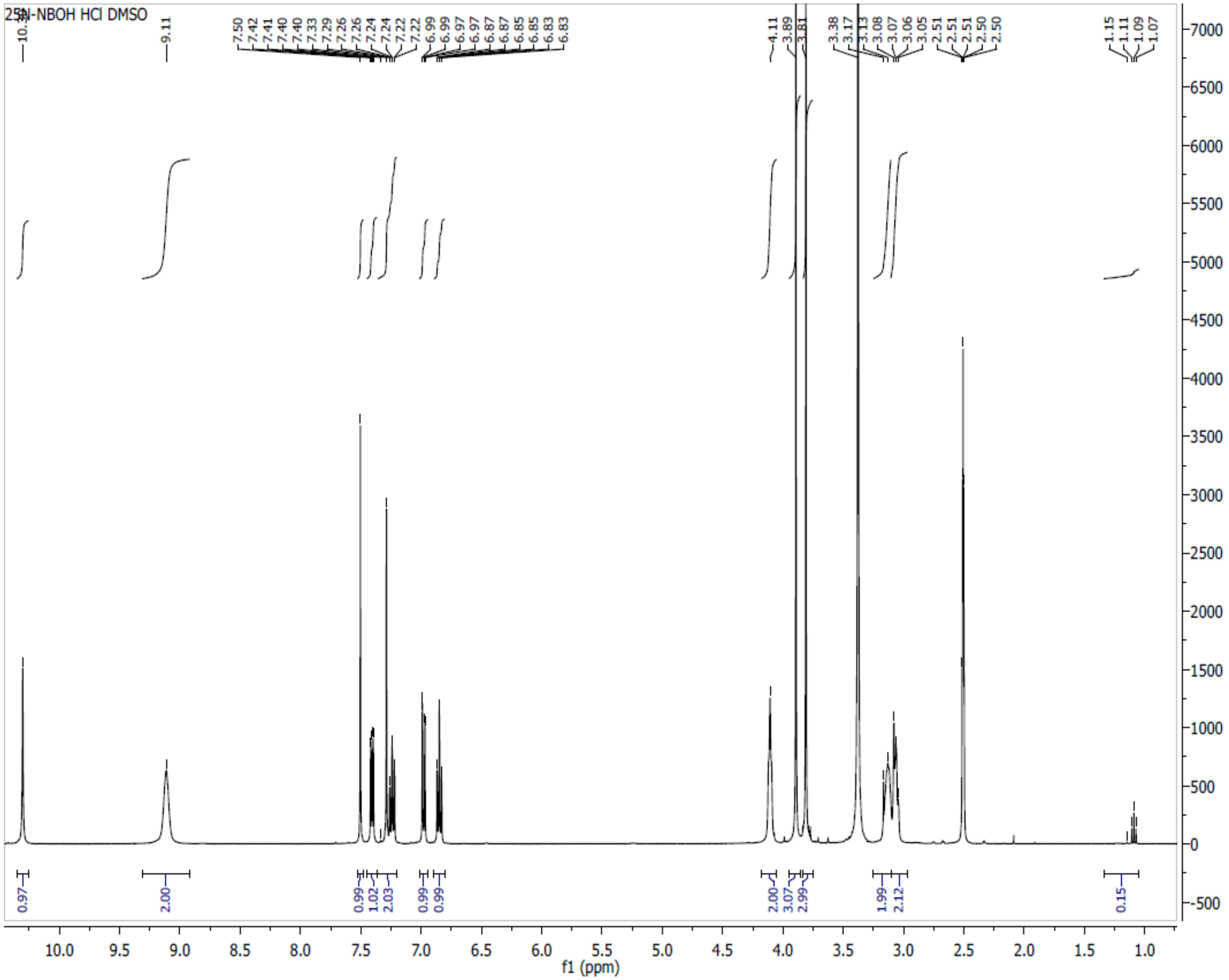

Spectra 3. ^{1}H NMR of 25N-NBOMe (4) HCl

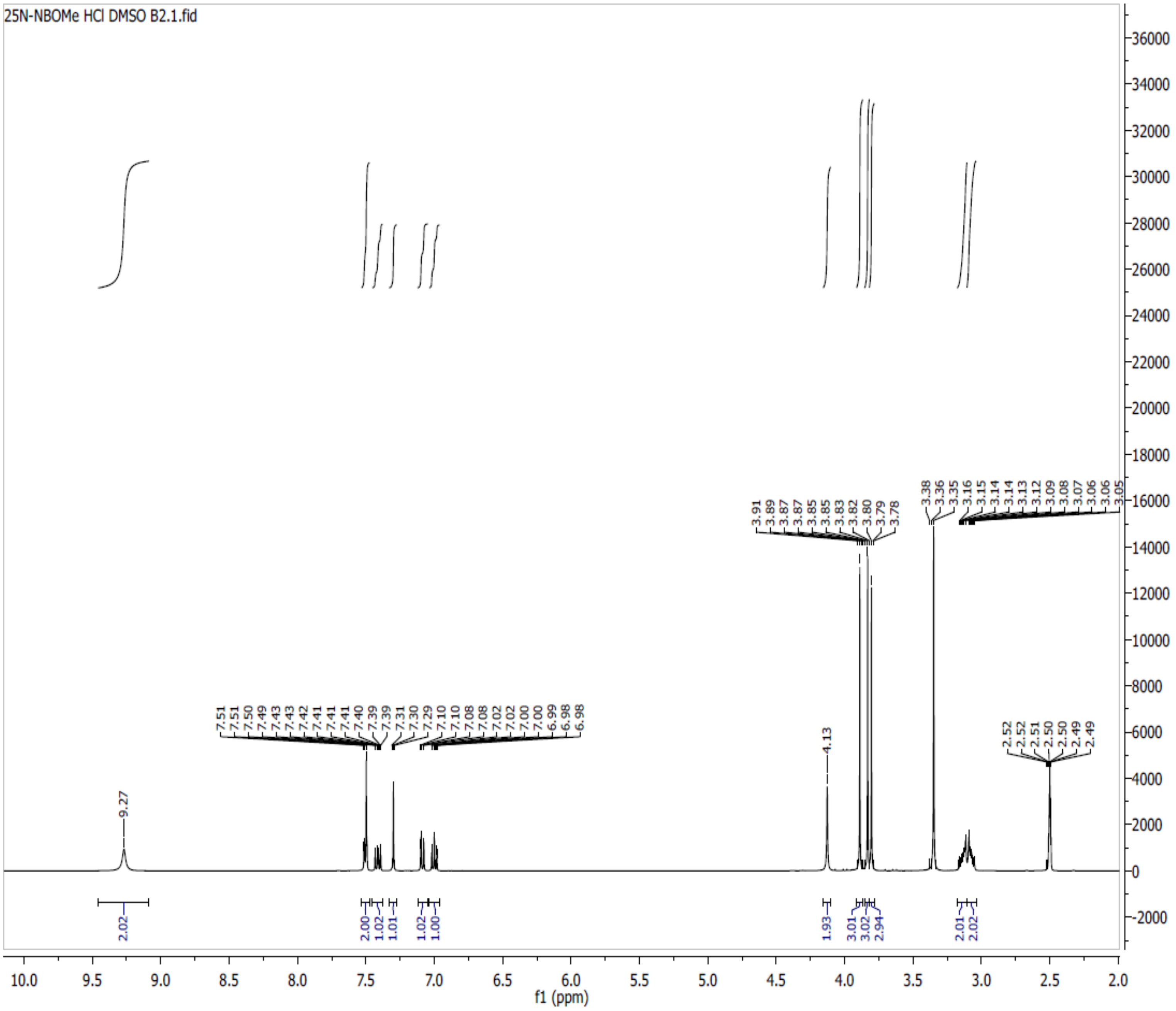

Spectra 4. ^{1}H NMR of 25N-NBOEt (5) HCl

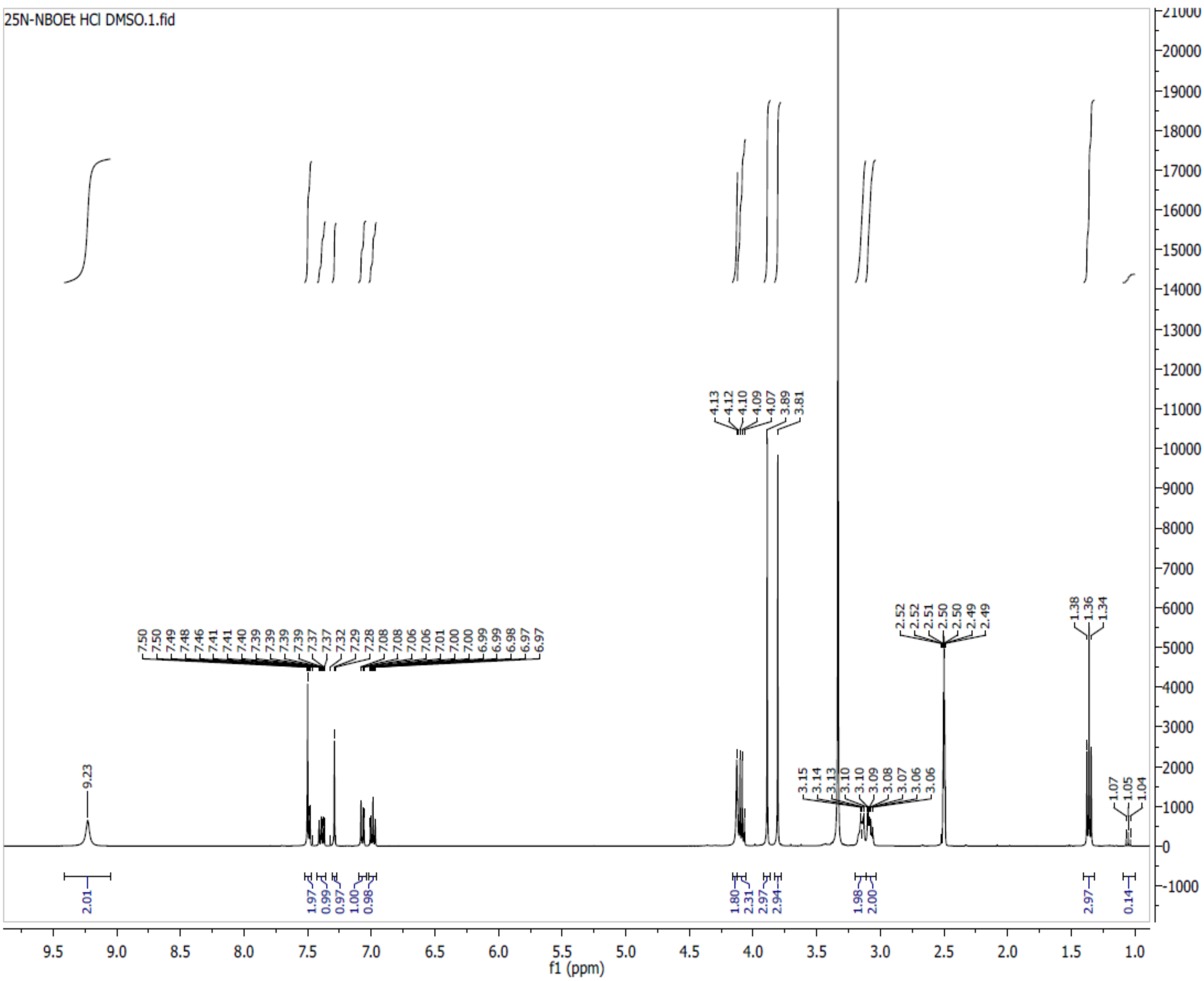

Spectra 5. ^{1}H NMR of 25N-NBMe (6) HCl

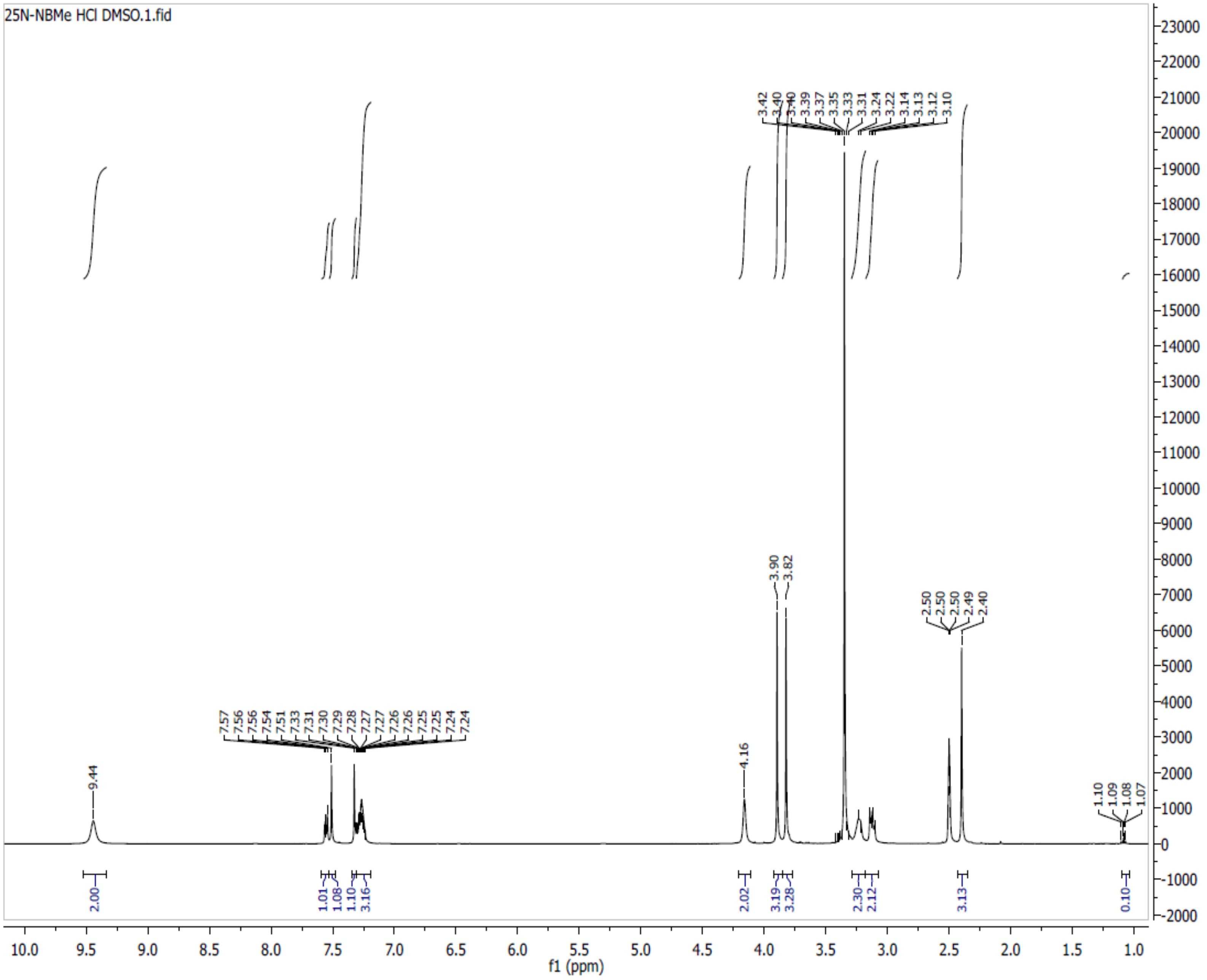

Spectra 6. ^{1}H NMR of 25N-NBF (7) HCl

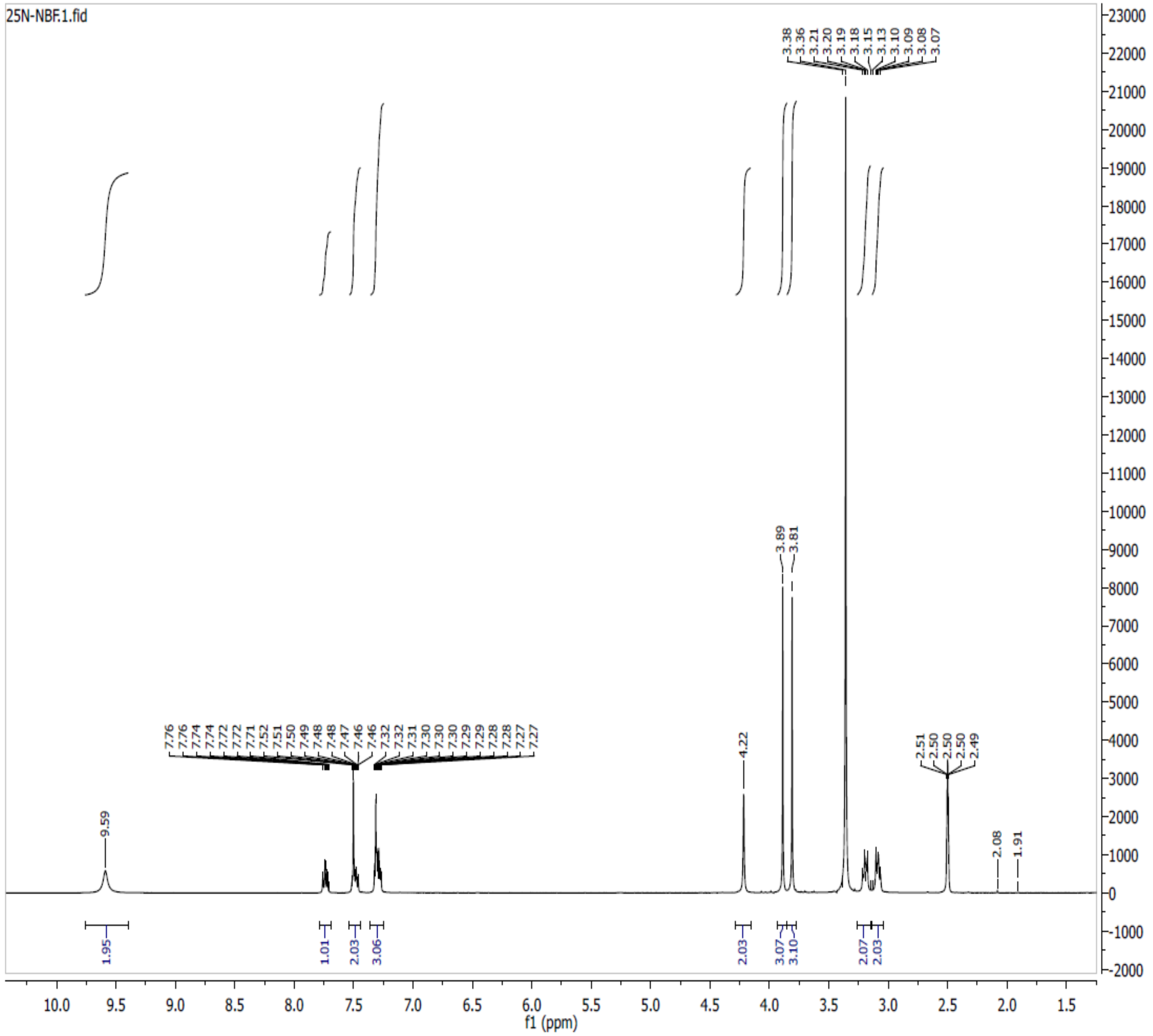

Spectra 7. ^{1}H NMR of 25N-NBCl (8) HCl

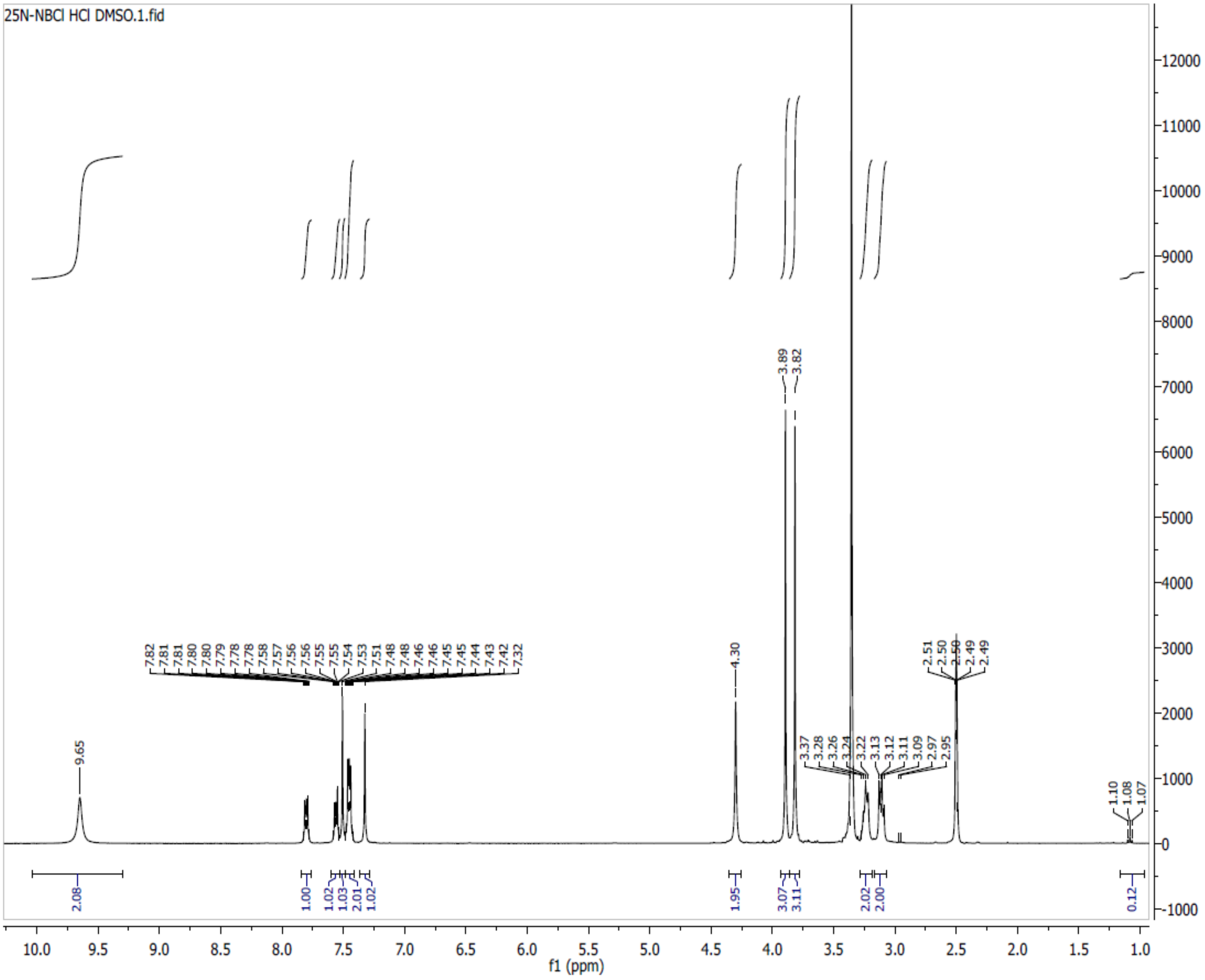

Spectra 8. ^{1}H NMR of 25N-NBBr (9) HCl

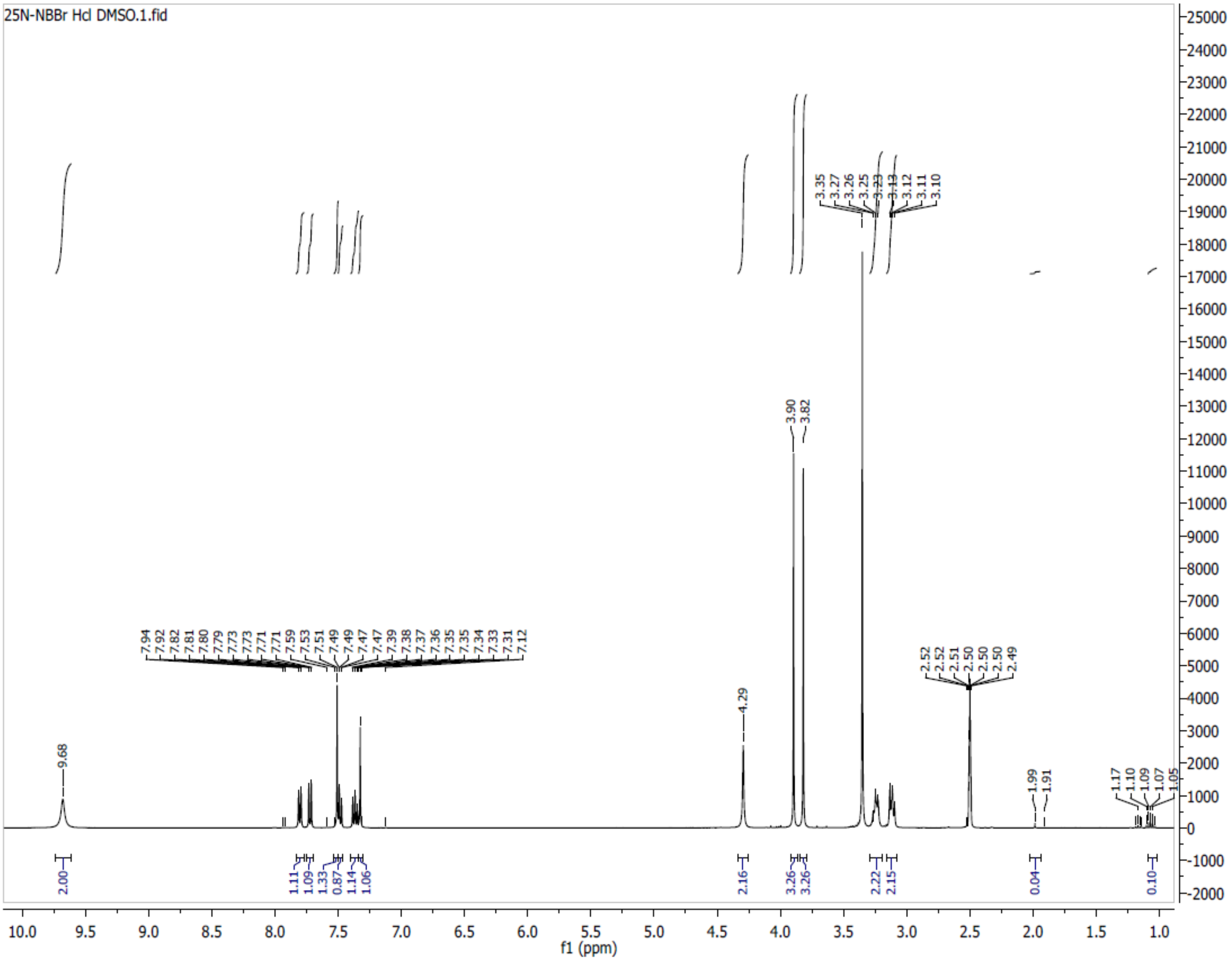

Spectra 9. ^{1}H NMR of 25N-NBI (10) HCl

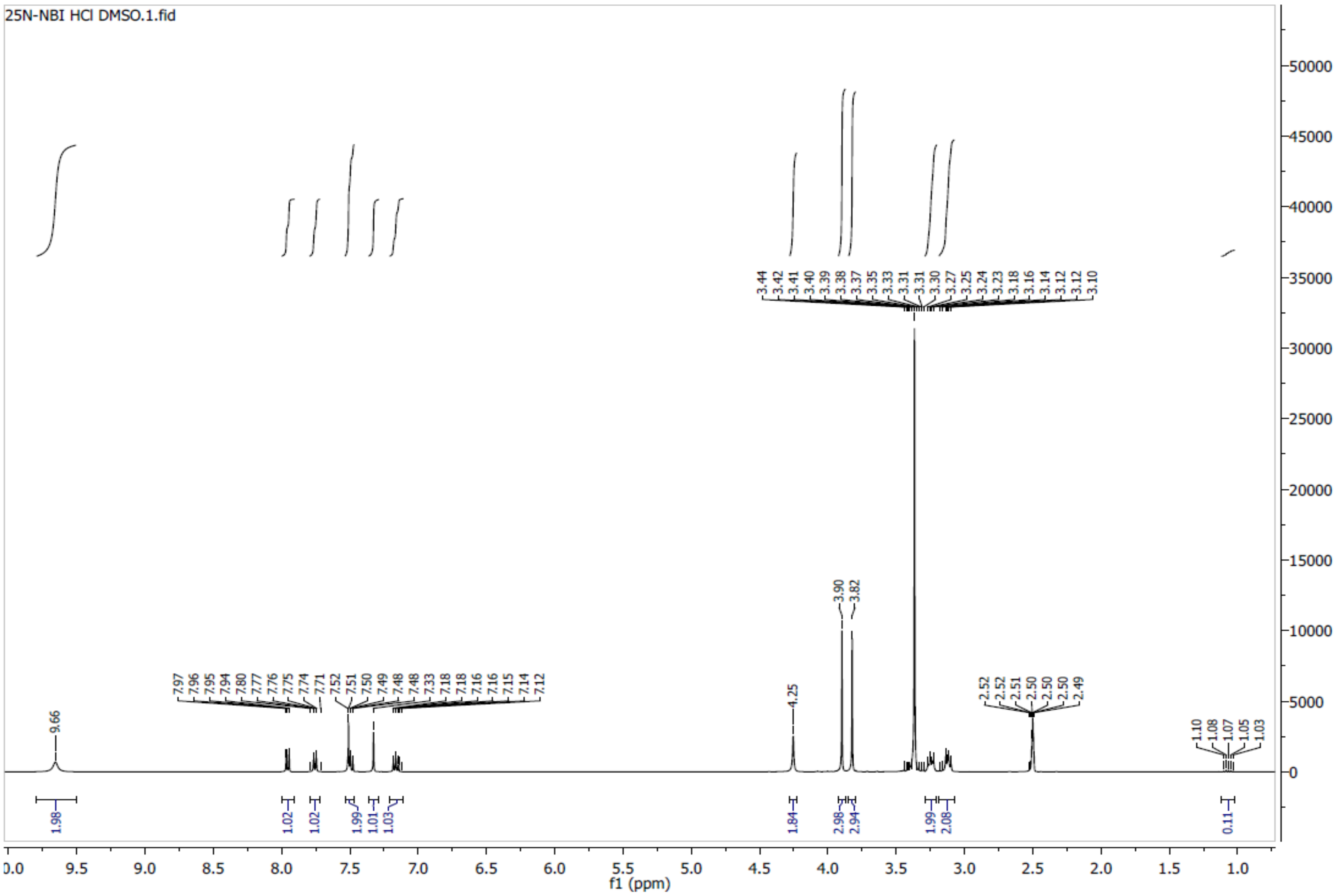

Spectra 10. ^{1}H NMR of 25N-NBOCF$_2$H (11) HCl

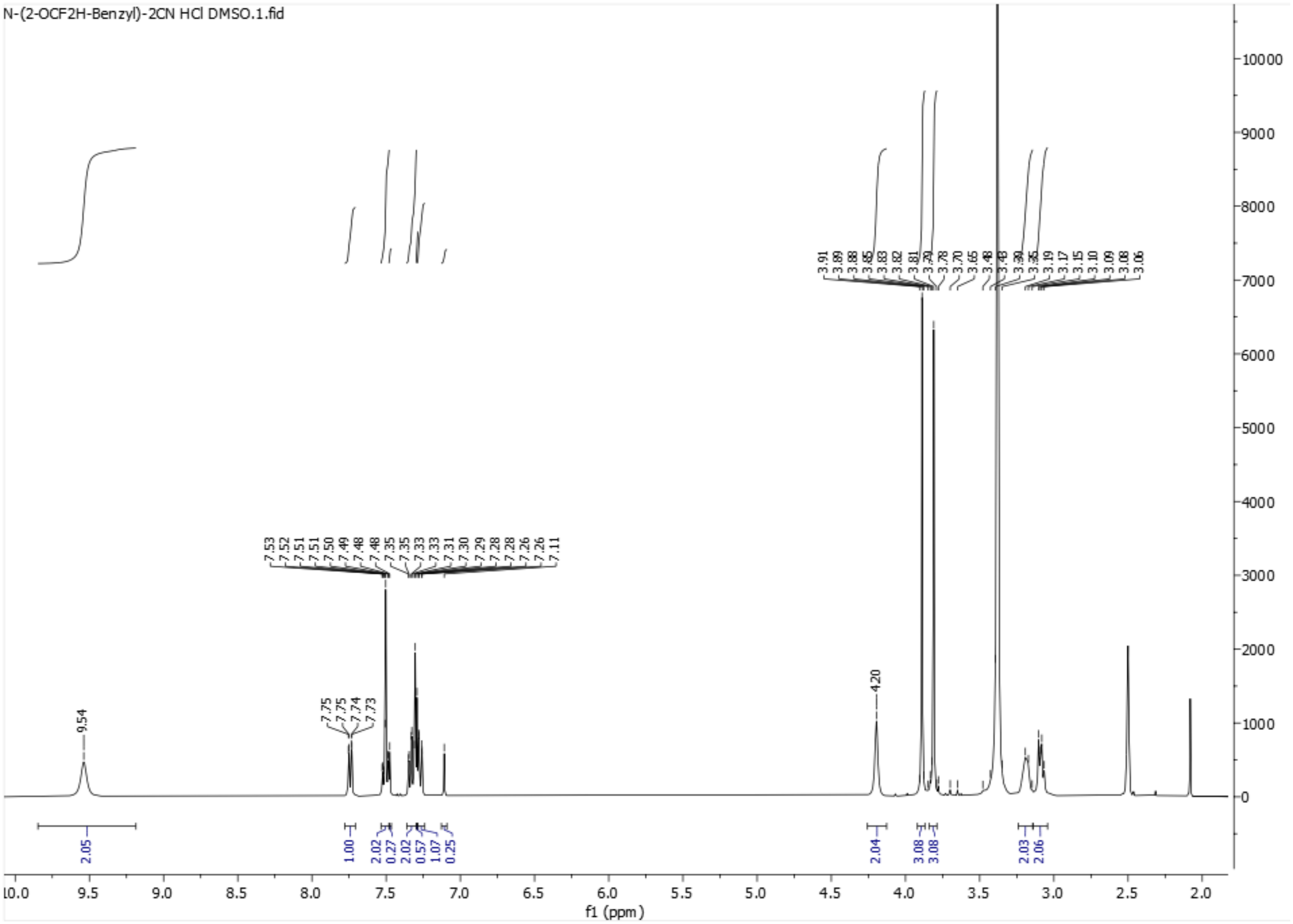

Spectra 11. ¹H NMR of 25N-NBOCF$_3$ (12) HCl

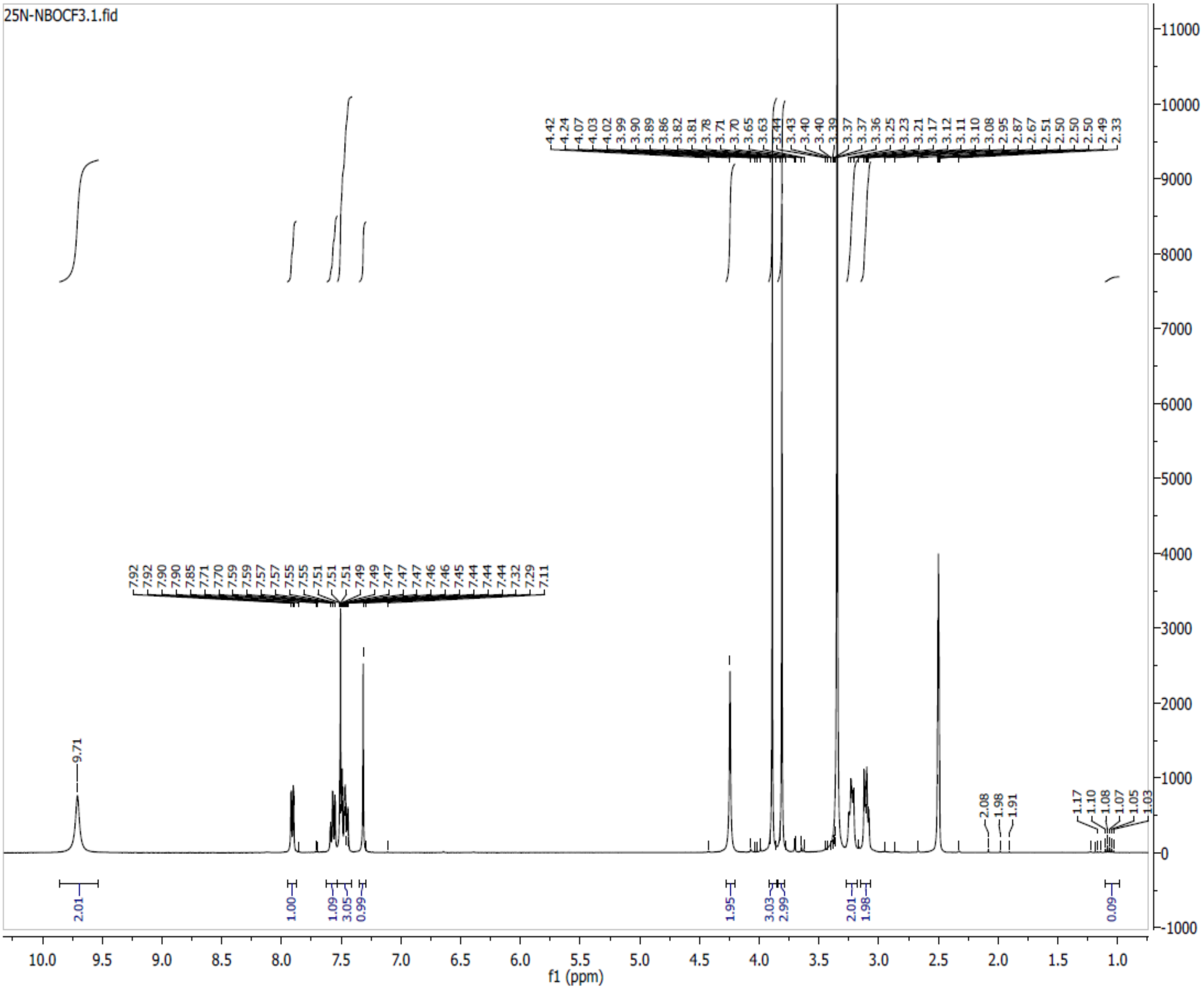

Spectra 12. ^{1}H NMR of 25N-NBMDF$_2$ (13) HCl

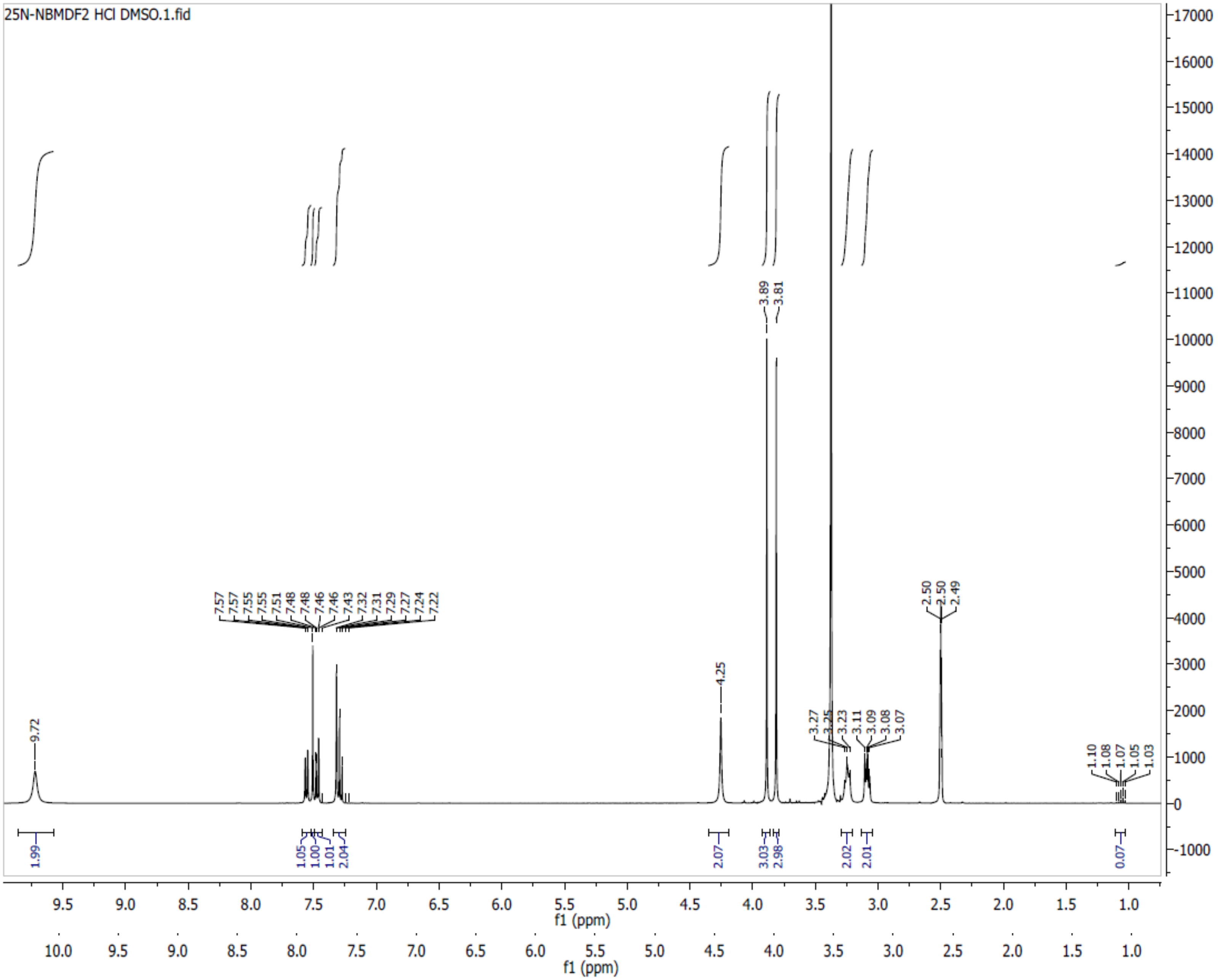

Spectra 13. ^{1}H NMR of 25N-NBCF$_3$ (14) HCl

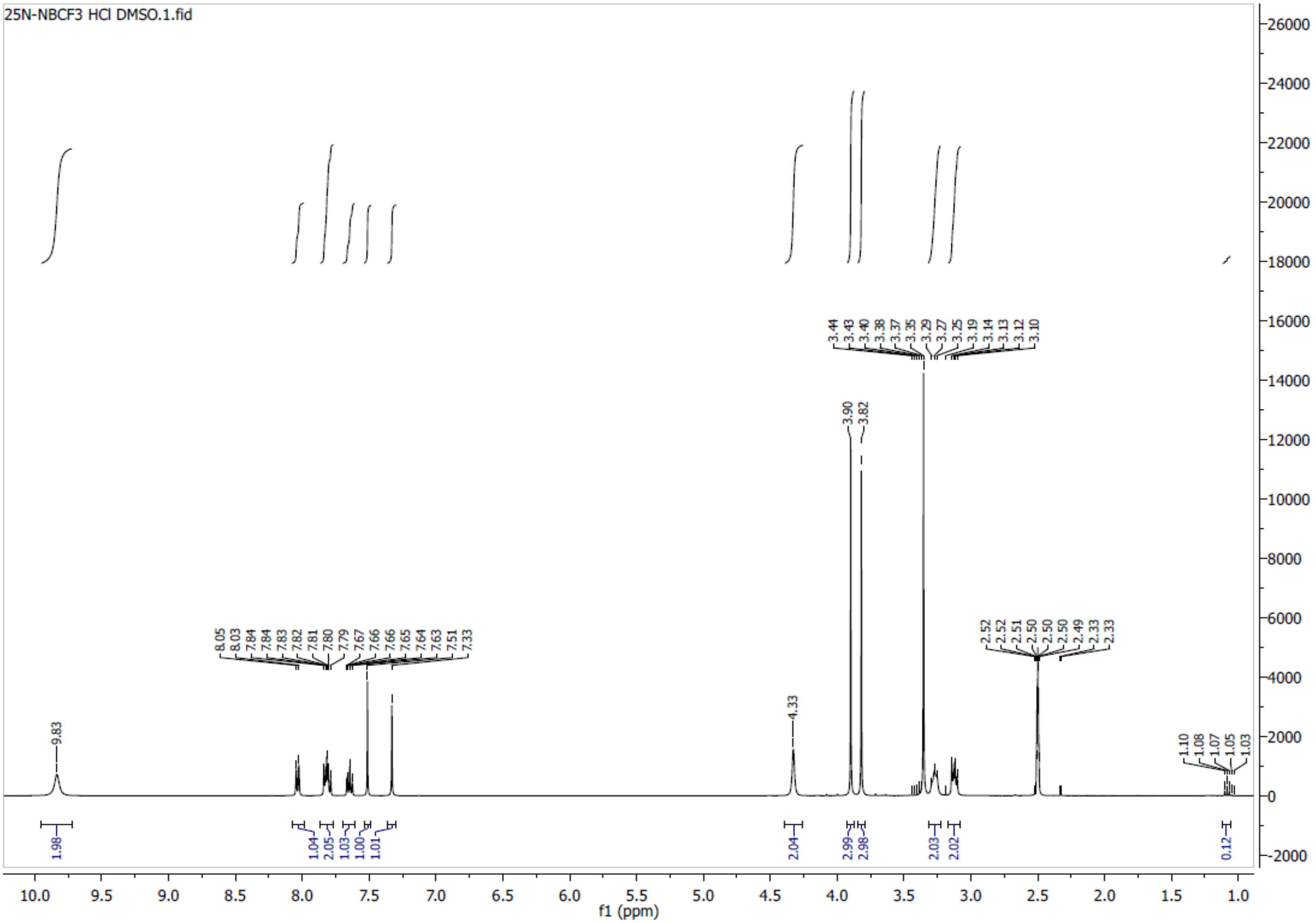

Spectra 14. ^{1}H NMR of 25N-NBNO$_2$ (15) HCl

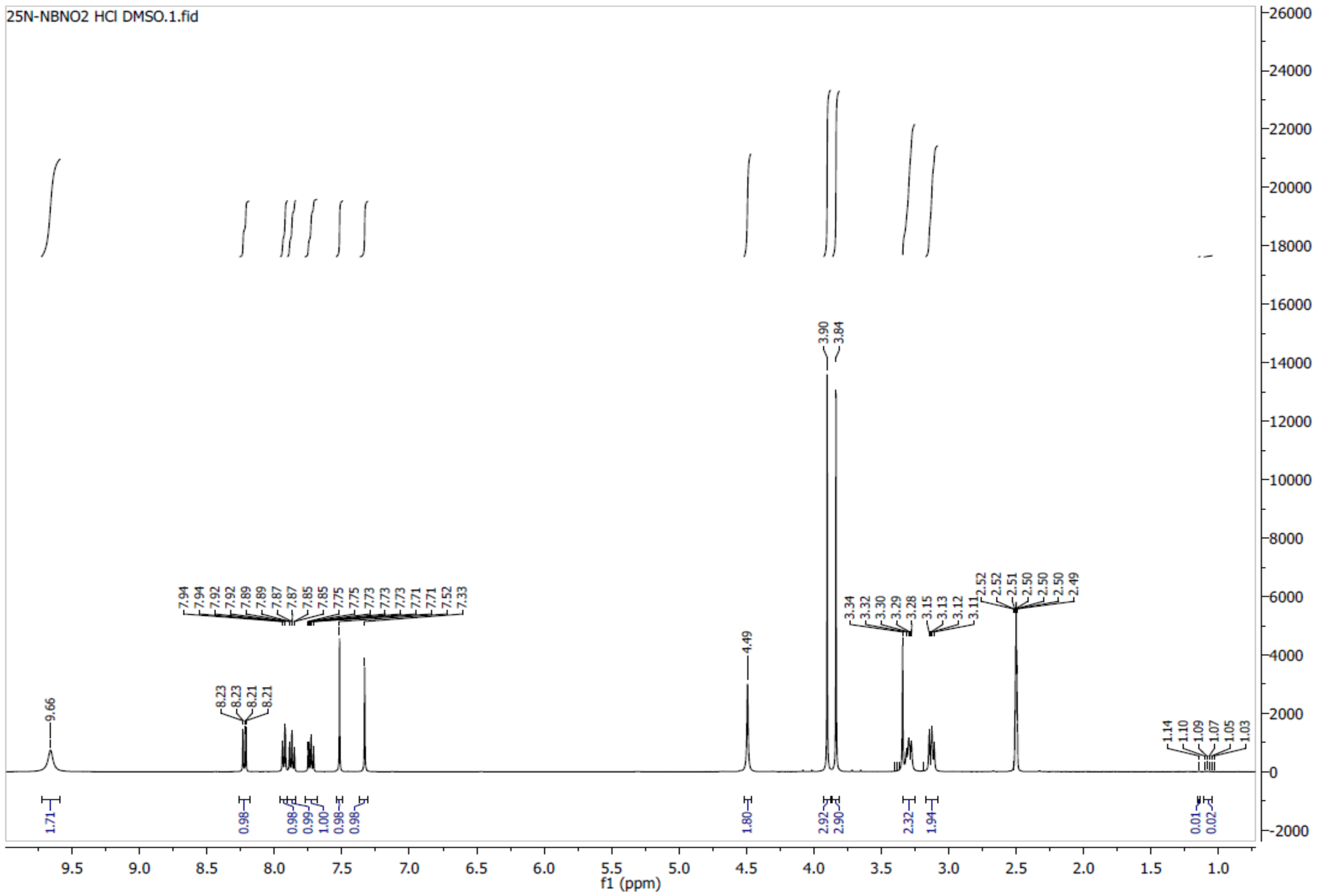

Spectra 15. ^{1}H NMR of 25N-N1-Nap (16) HCl

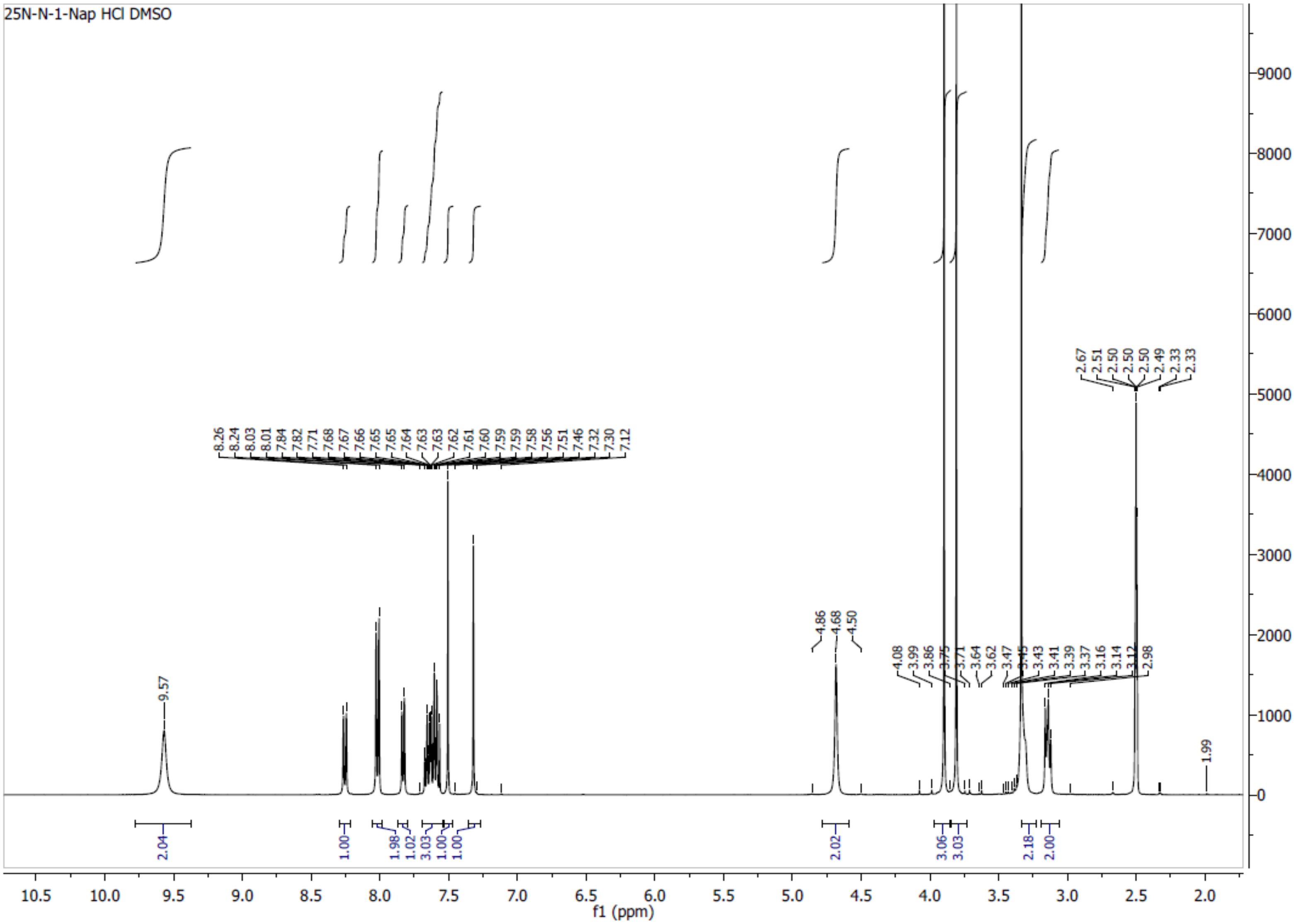

Spectra 16. ^{1}H NMR of 25N-NBPh (17) HCl

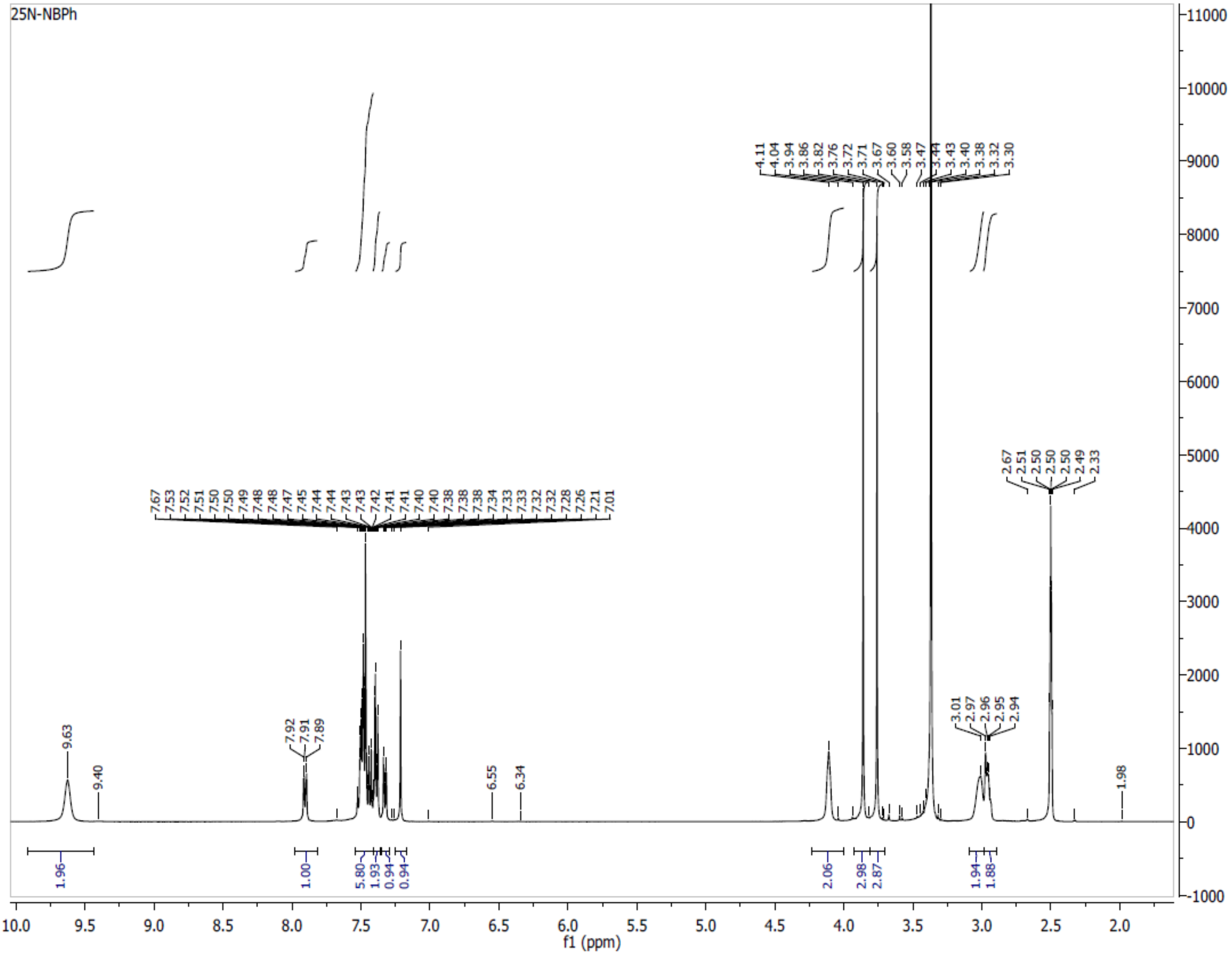

Spectra 17. ^{1}H NMR of 25N-NB-2-OH-3-Me (18) HCl

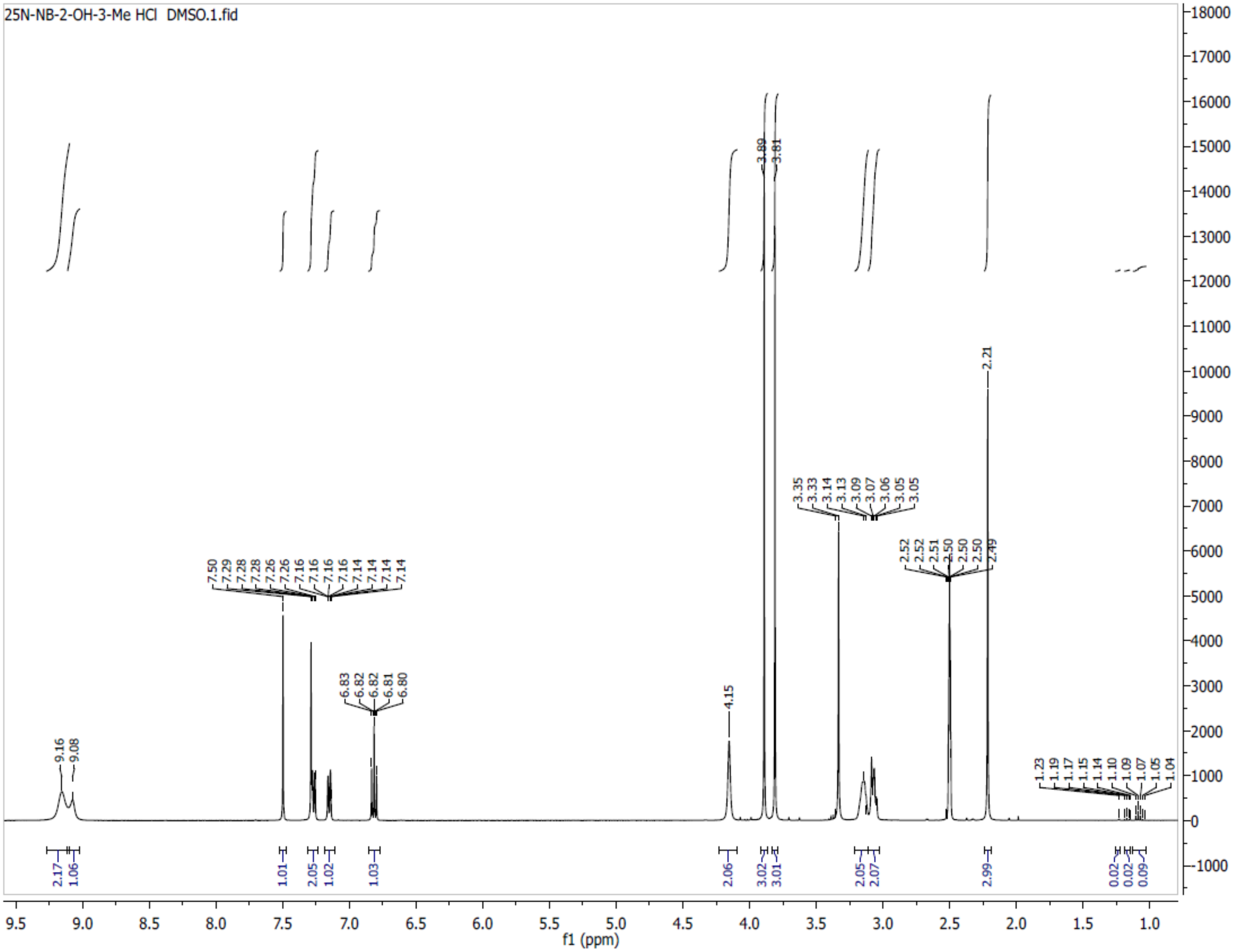

Spectra 18. ^{1}H NMR of 25N-NB-2-MeO-3-F (19) HCl

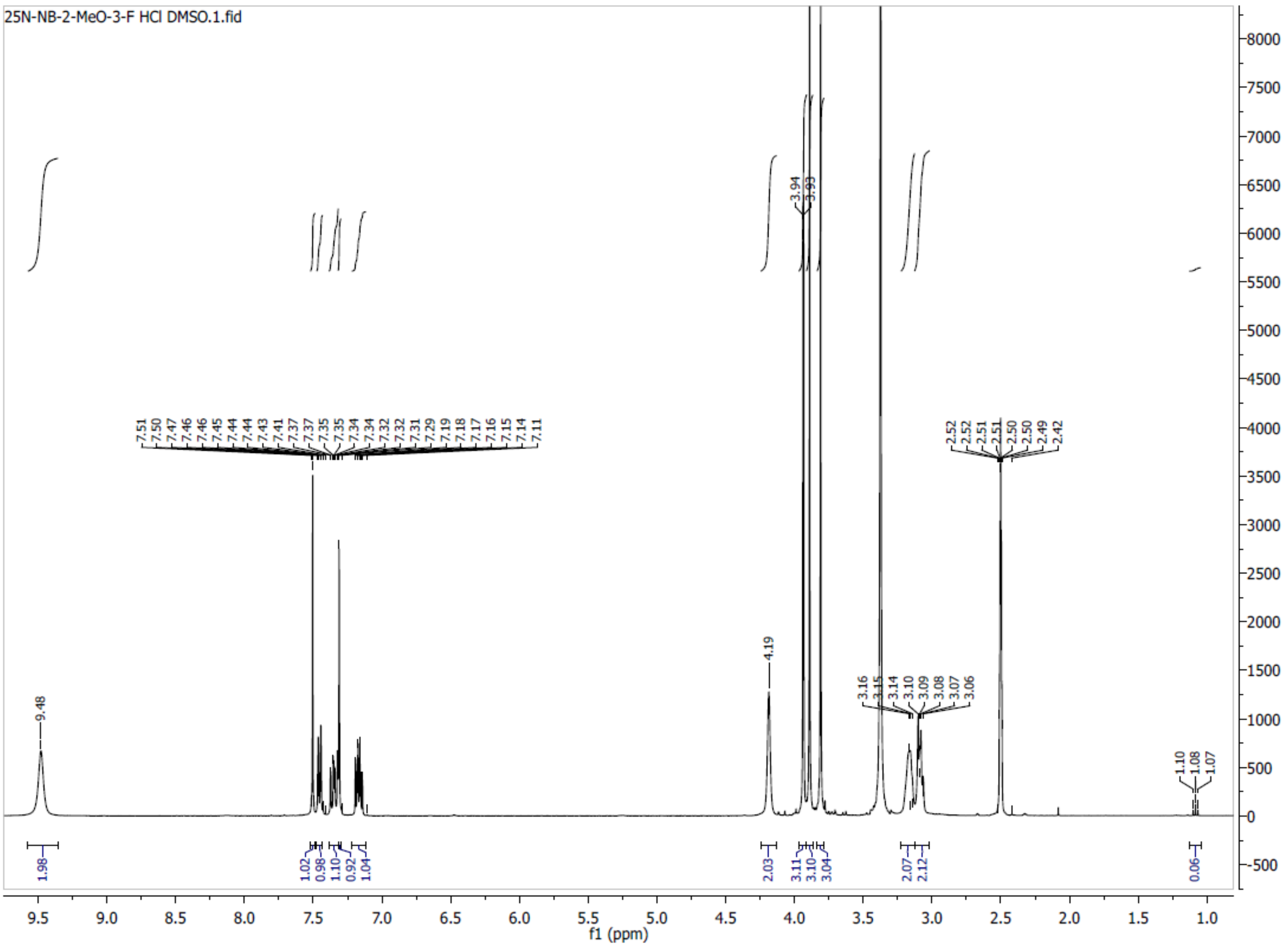

Spectra 19. ^{1}**H NMR of 25N-NB-2,5-DiMeO (20) HCl**

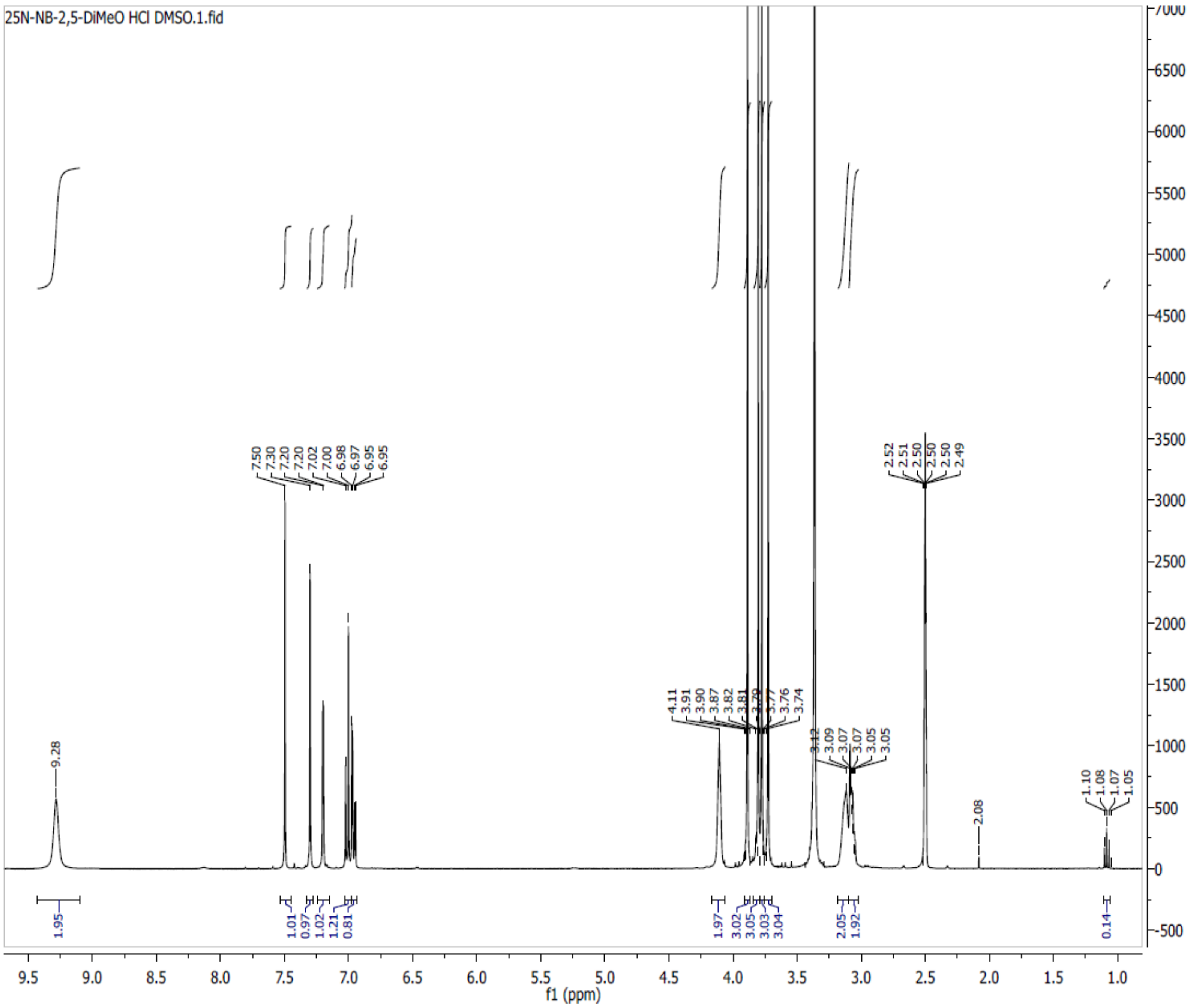

Spectra 20. ^{1}H NMR of 25N-NB-3-OH (21) HCl

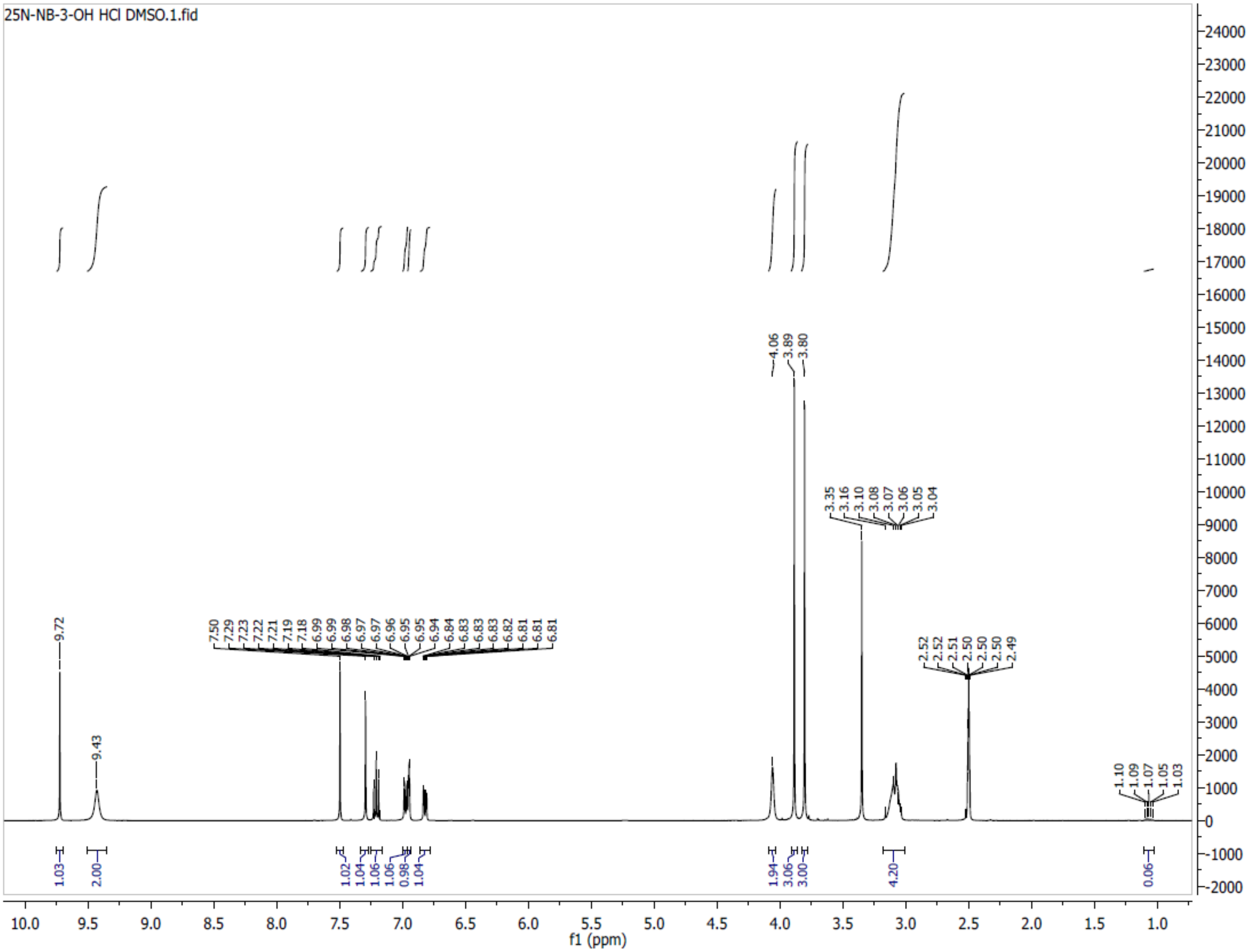

Spectra 21. ^{1}H NMR of 25N-NB-3-Me (22) HCl

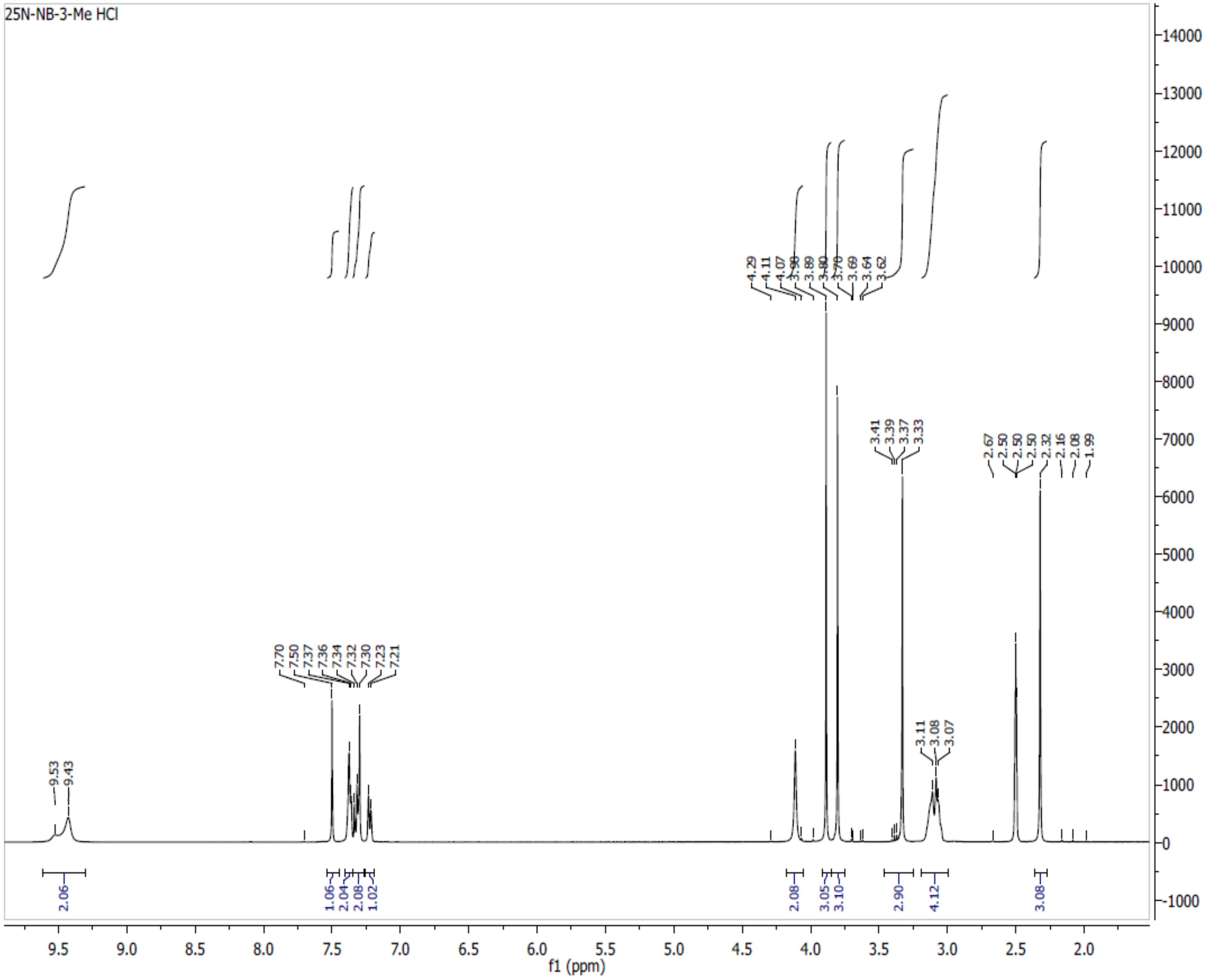

Spectra 22. ^{1}H NMR of 25N-NB-4-Me (23) HCl

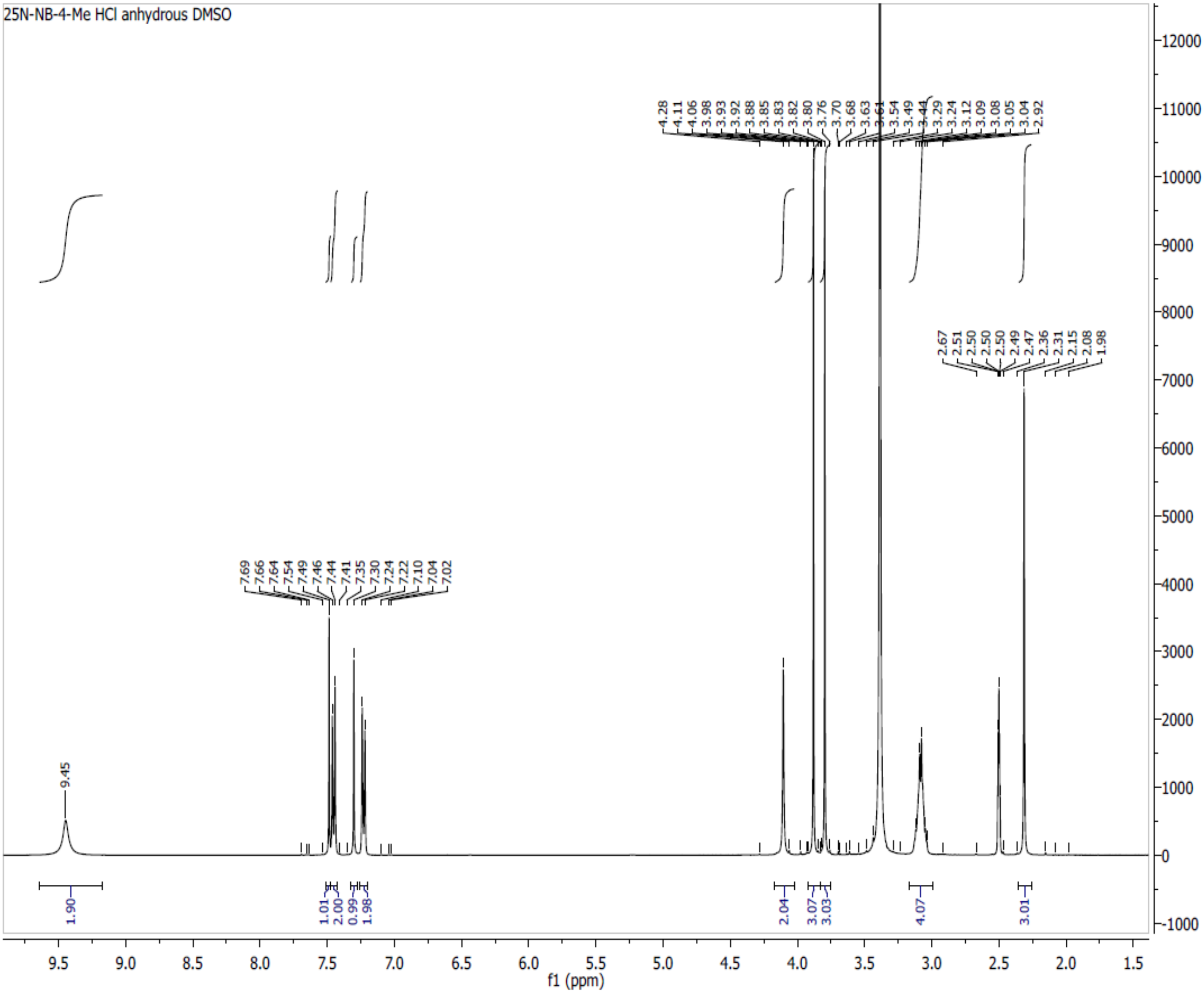

Spectra 23. ^{1}H NMR of 25N-NB-3-F (24) HCl

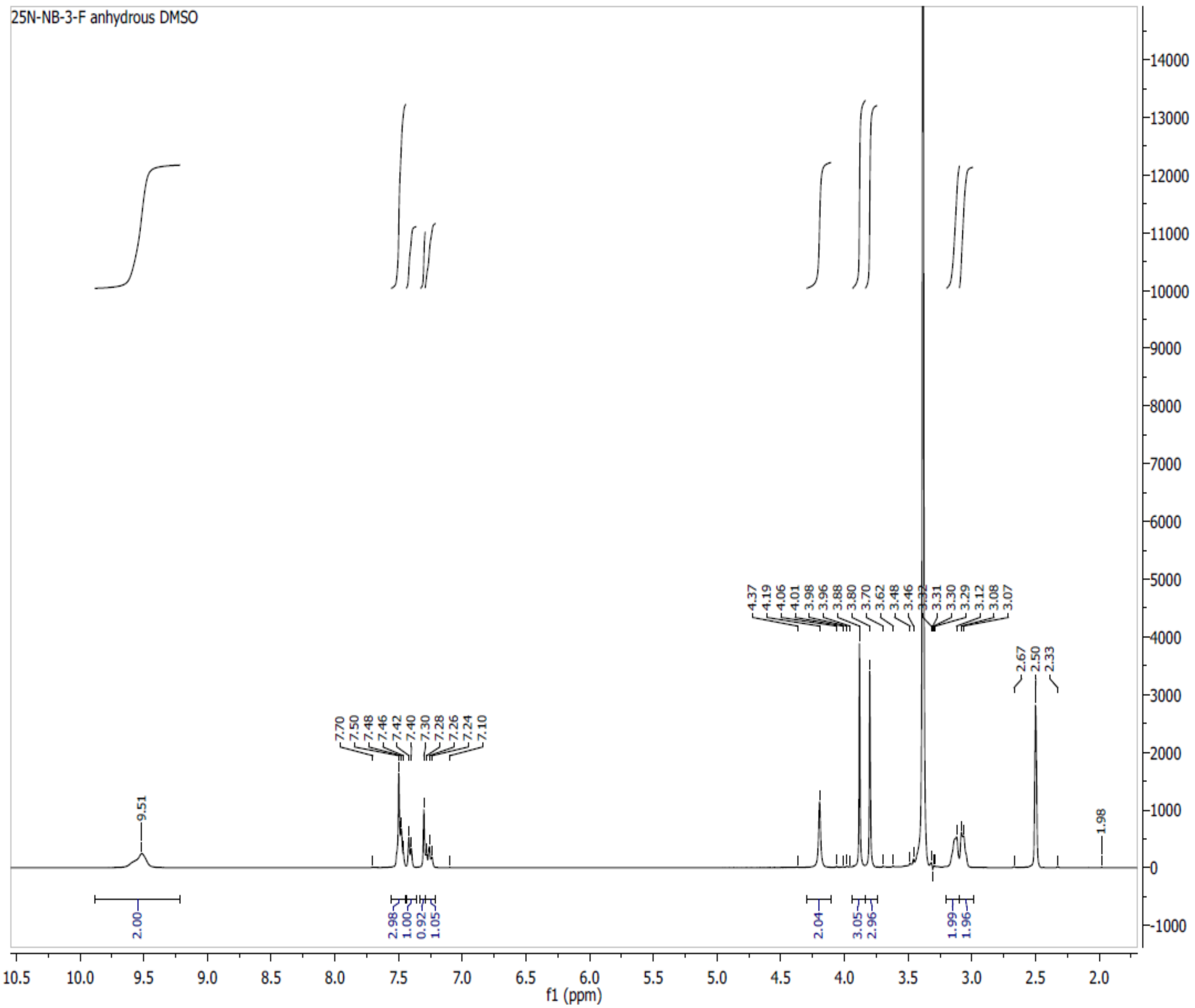

Spectra 24. ^{1}H NMR of 25N-NB-4-F (25) HCl

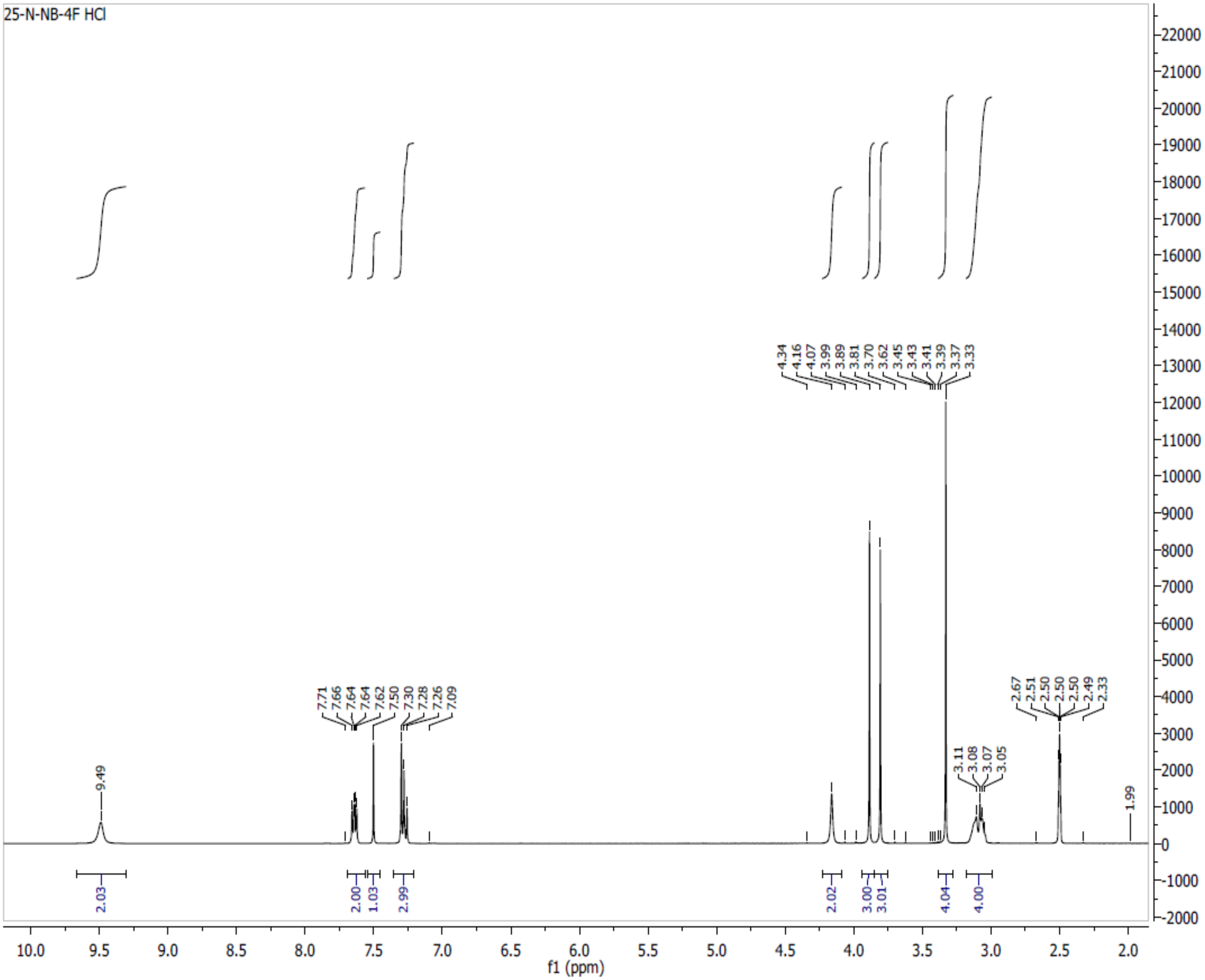

Spectra 25. **^{1}H NMR of 25D-N1-Nap (26) HCl**

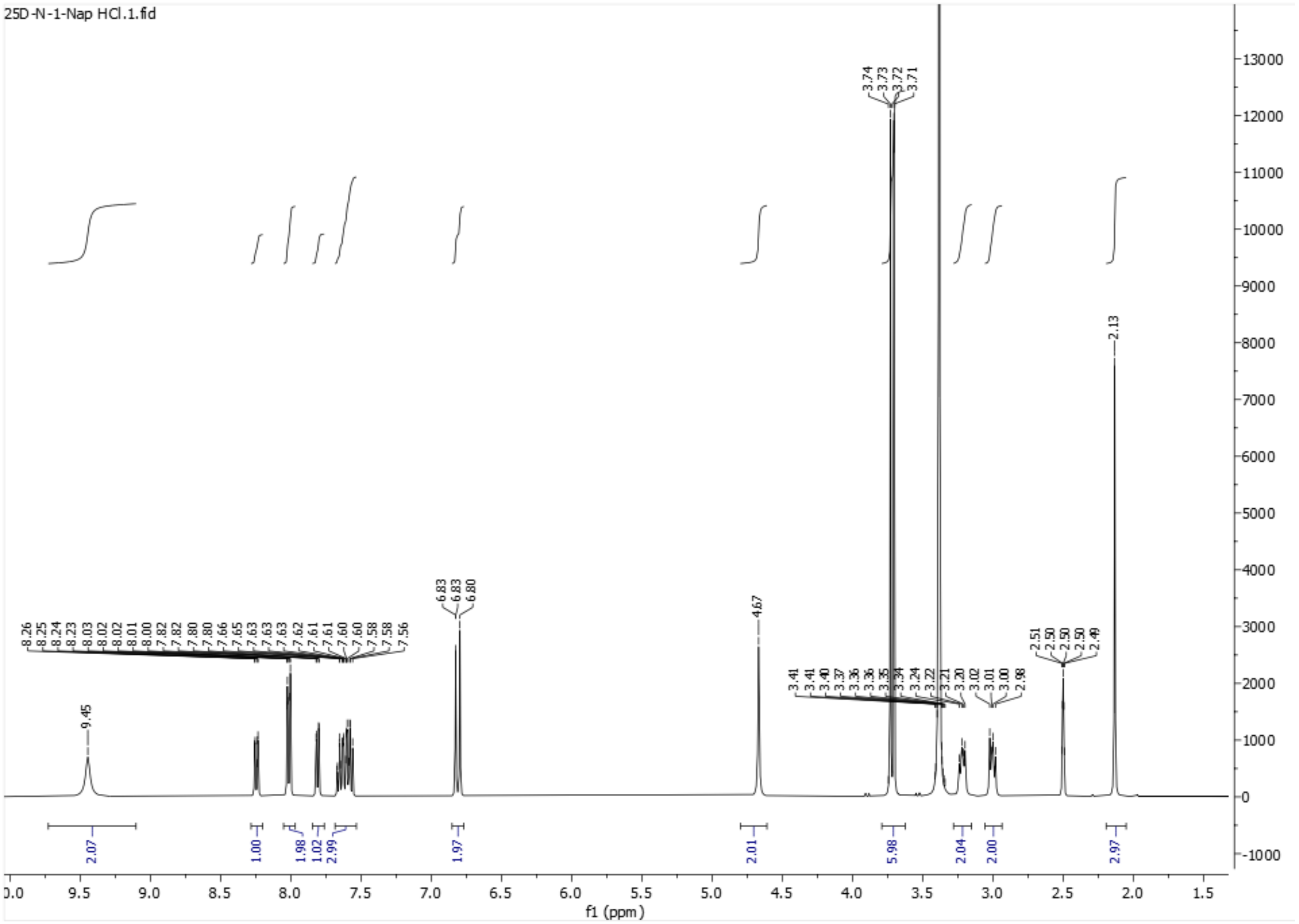

Spectra 26. **^{1}H NMR of 25D-NBPh (27) HCl**

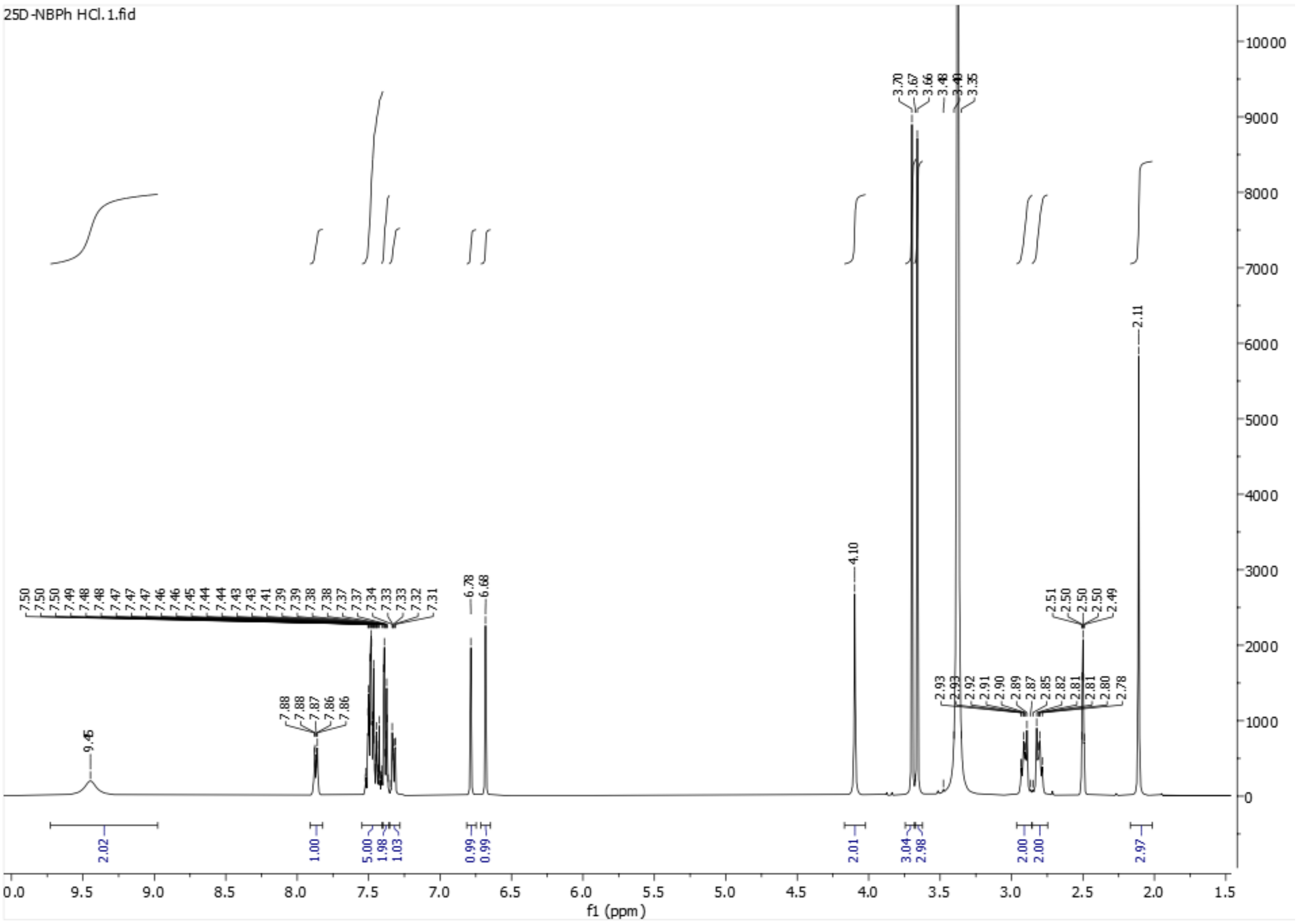

Spectra 27. ^{1}H NMR of 25O-N1-Nap (28) HCl

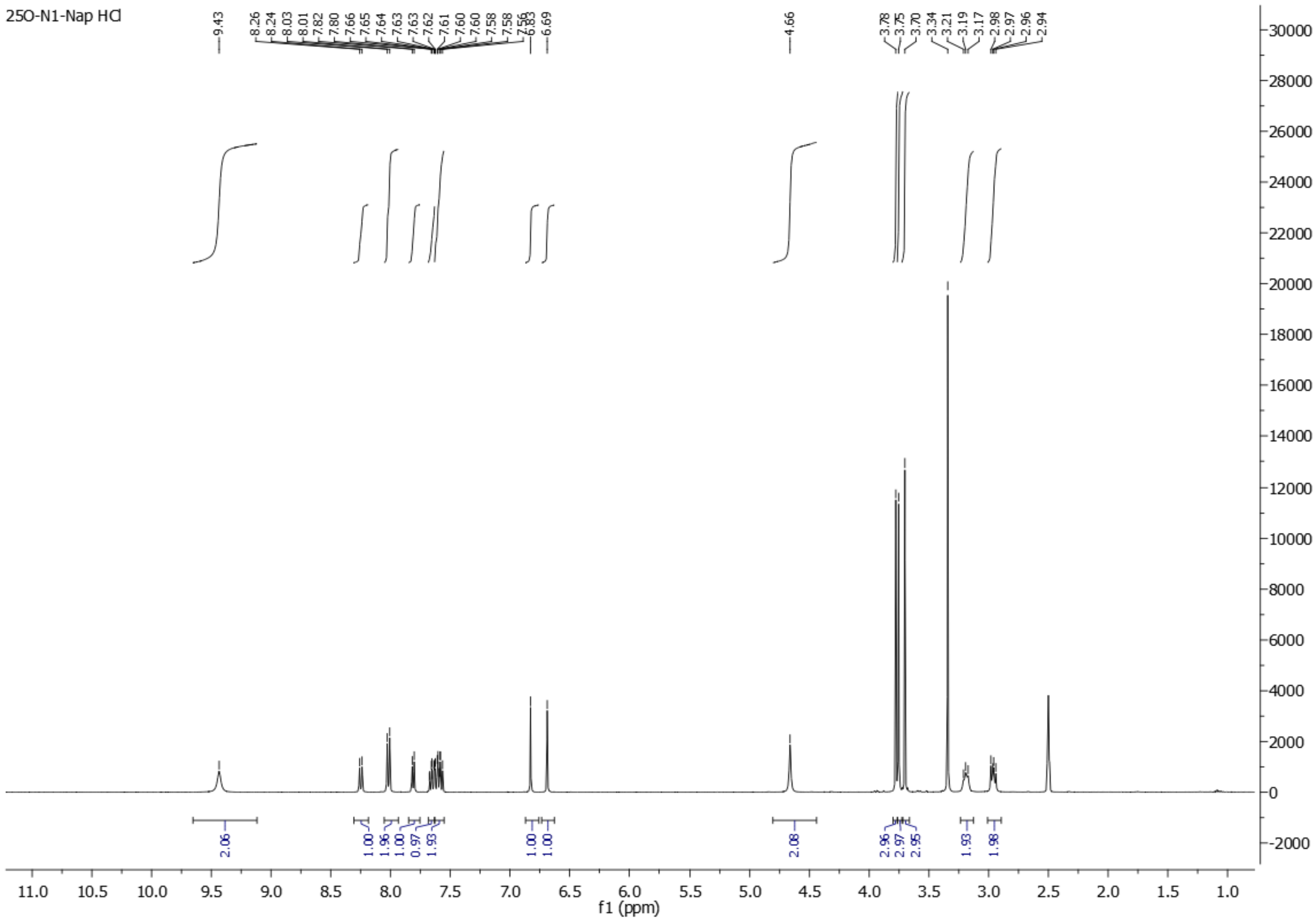

Spectra 28. ^{1}H NMR spectrum of 2C2-N1-Nap (29) HCl

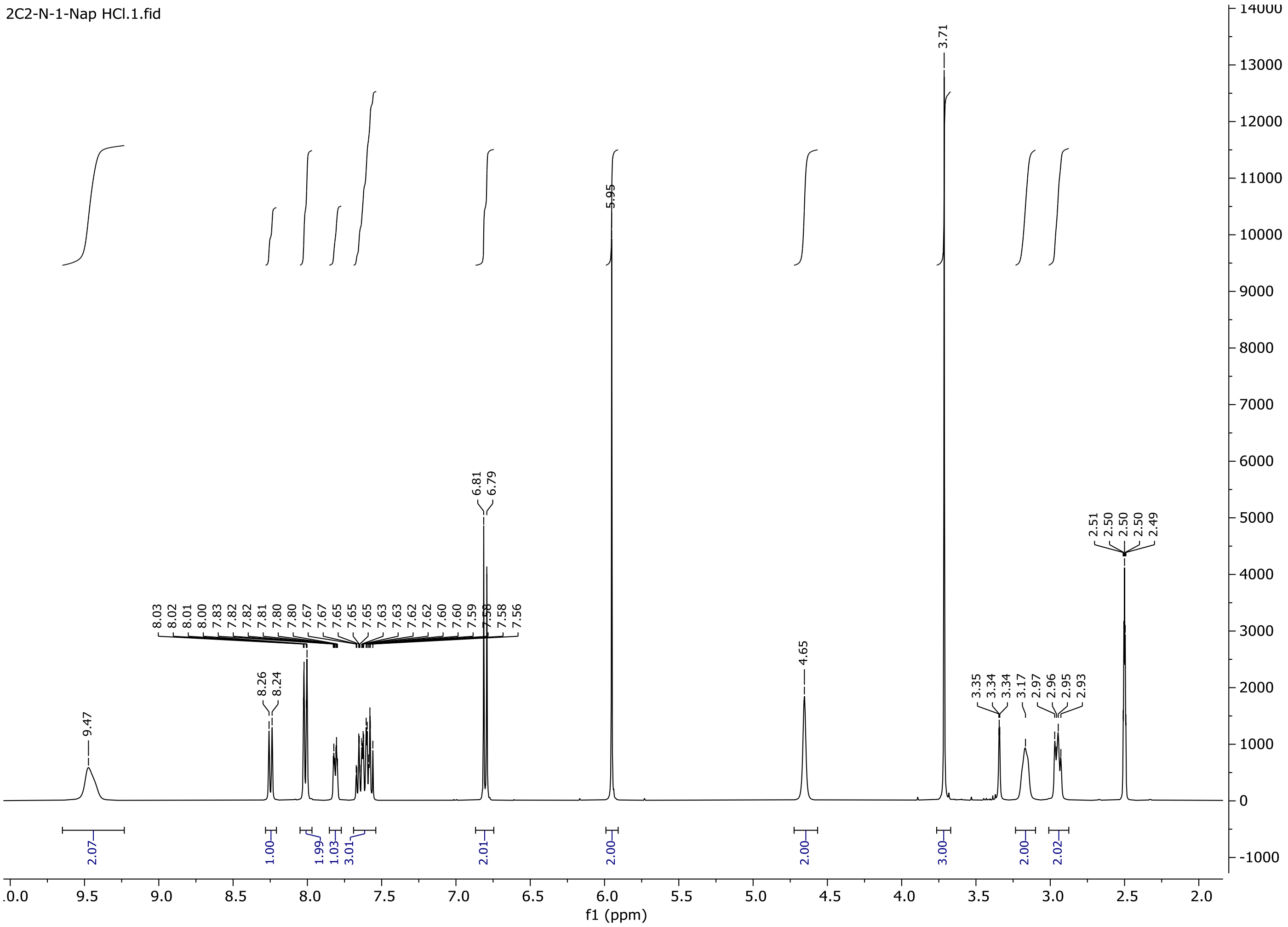

Spectra 29. ^{1}H NMR spectrum of **Ψ-DOB-N1-Nap (30) HCl**

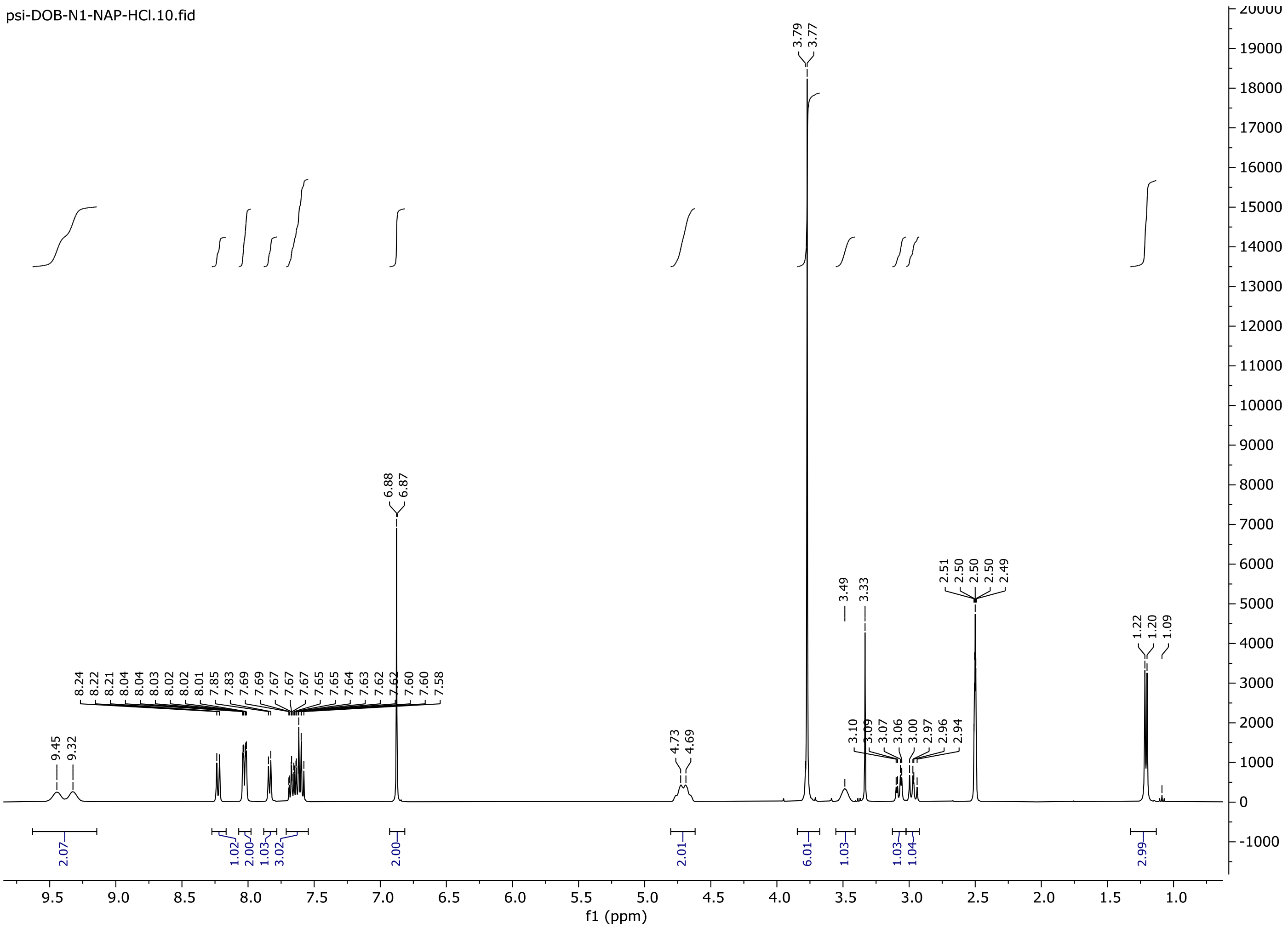

Spectra 30. ^{1}H NMR of 2C2-NBOMe (31) HCl

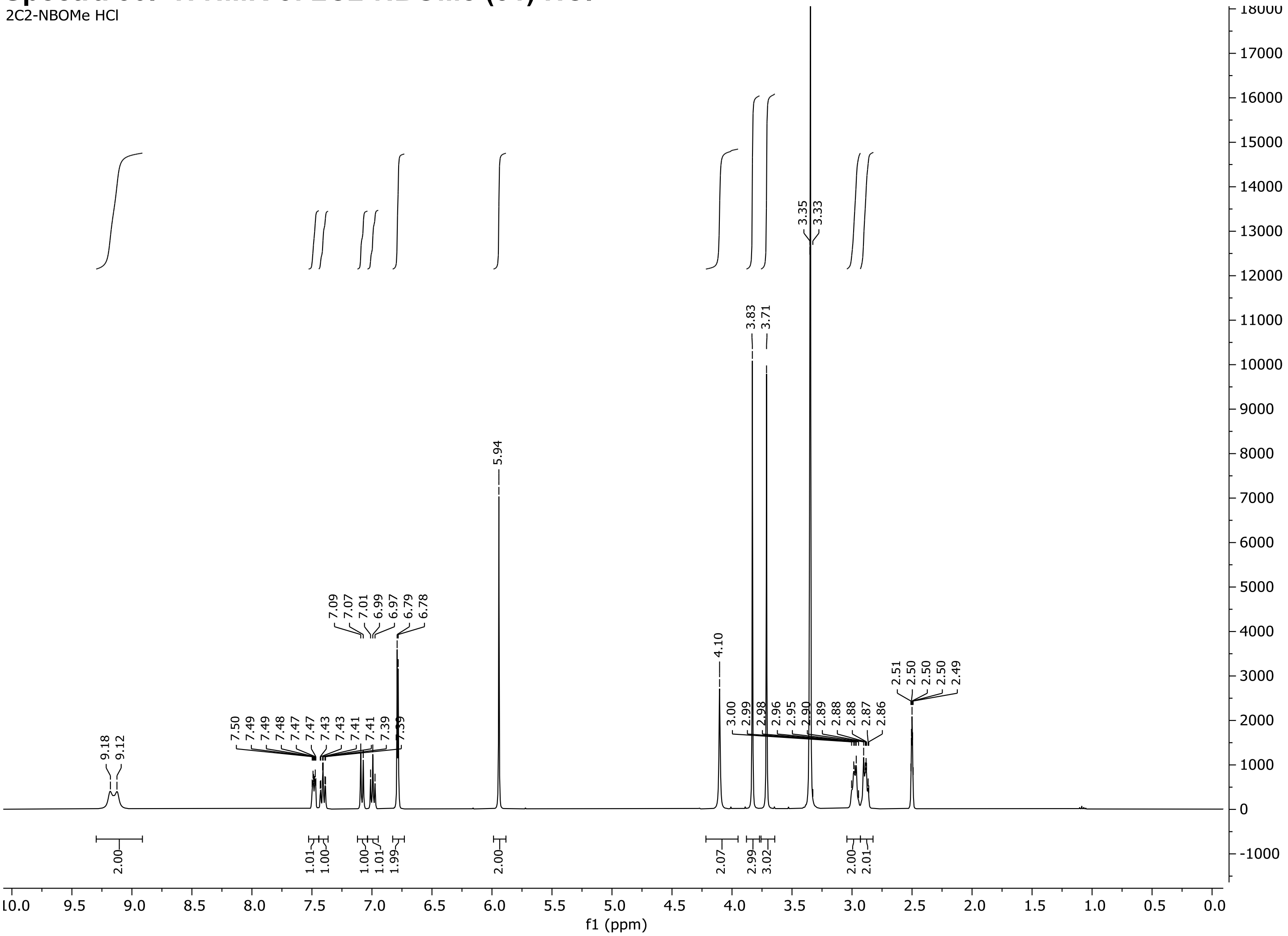

Spectra 31. ^{1}H NMR of 25O-NBOMe (32) HCl

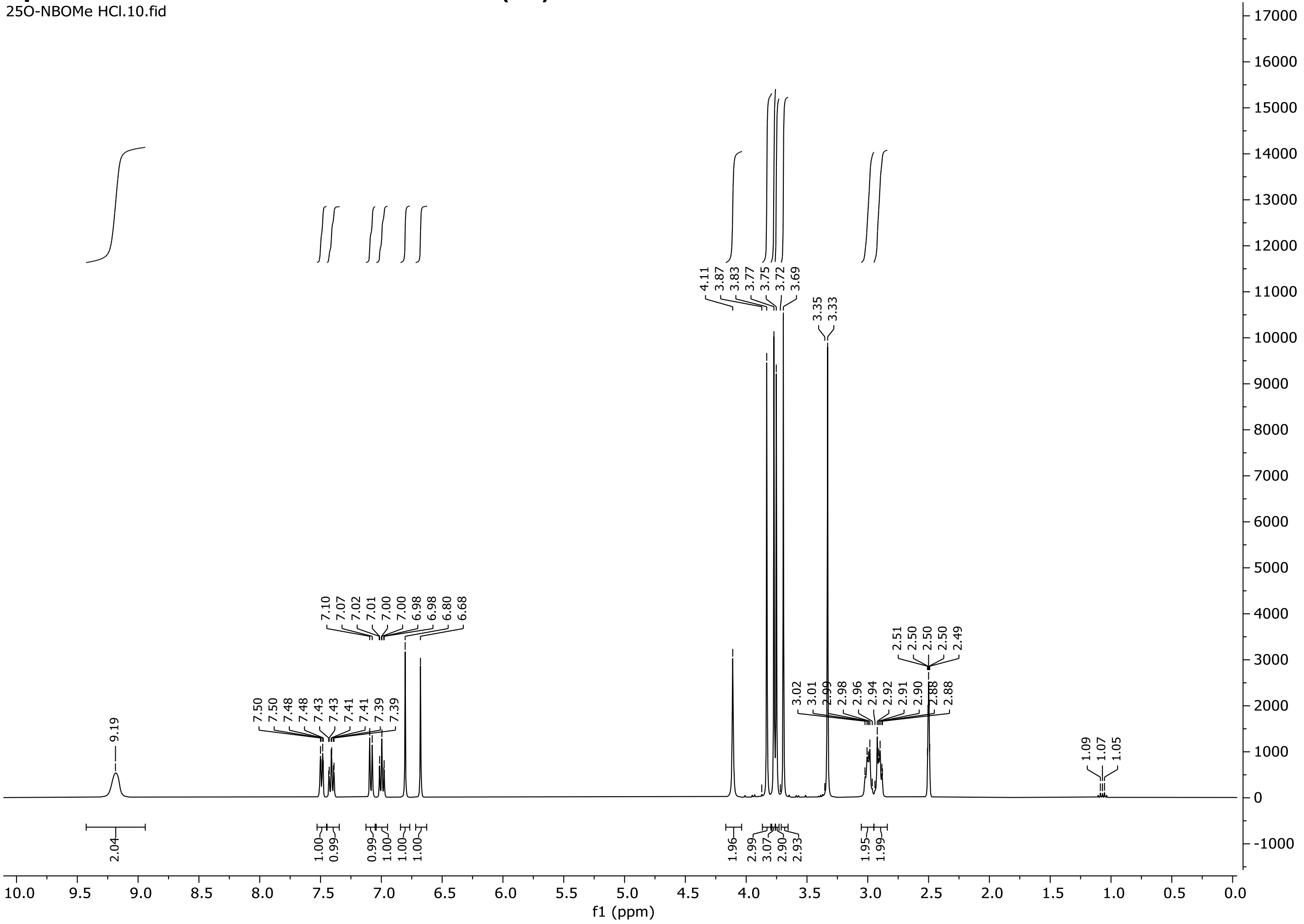

Spectra 32. ^{1}H NMR of 25O-NBcP (33) HCl

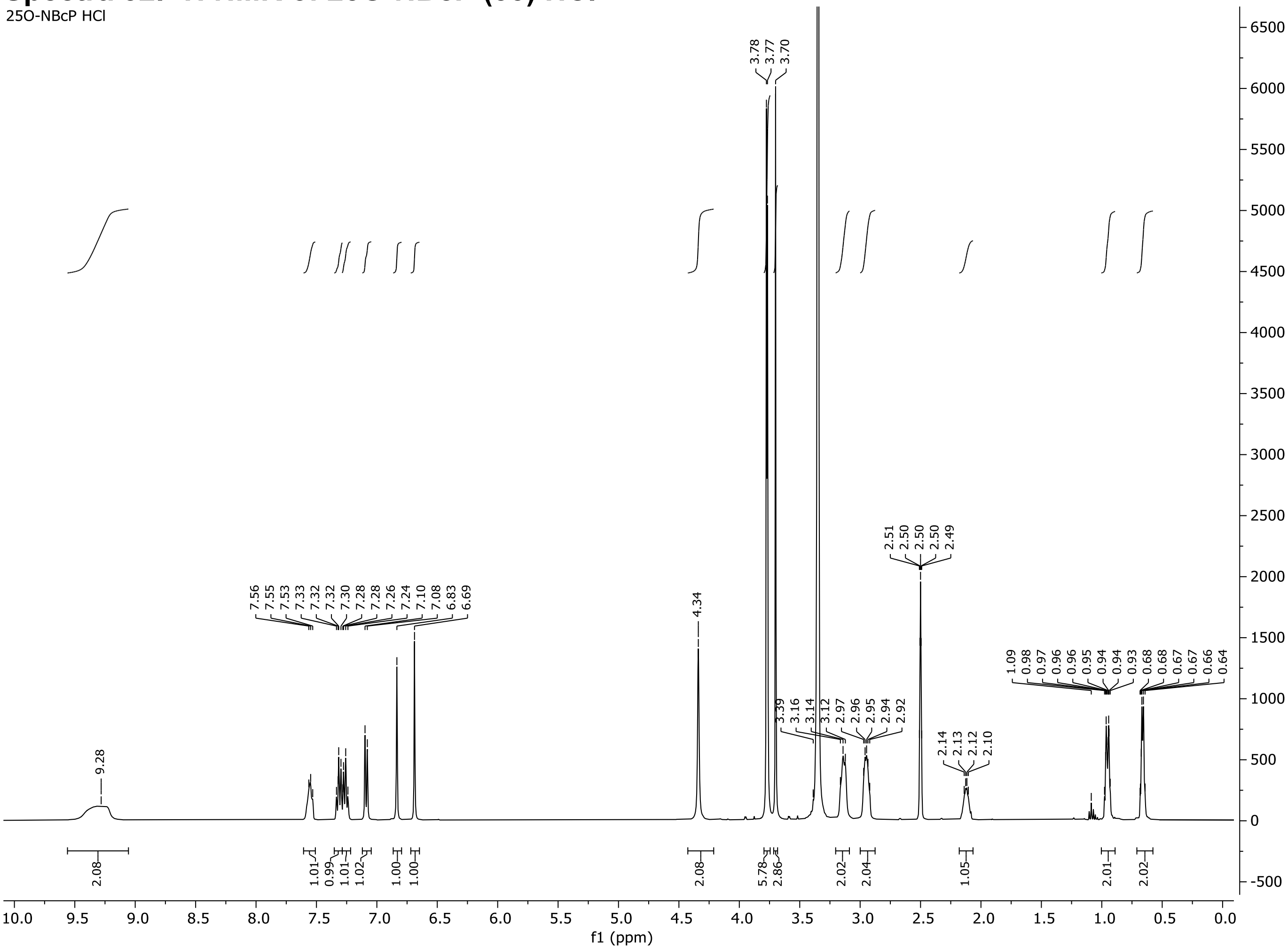

Spectra 33. ^{1}H NMR of 25O-NB-3-I (34) HCl

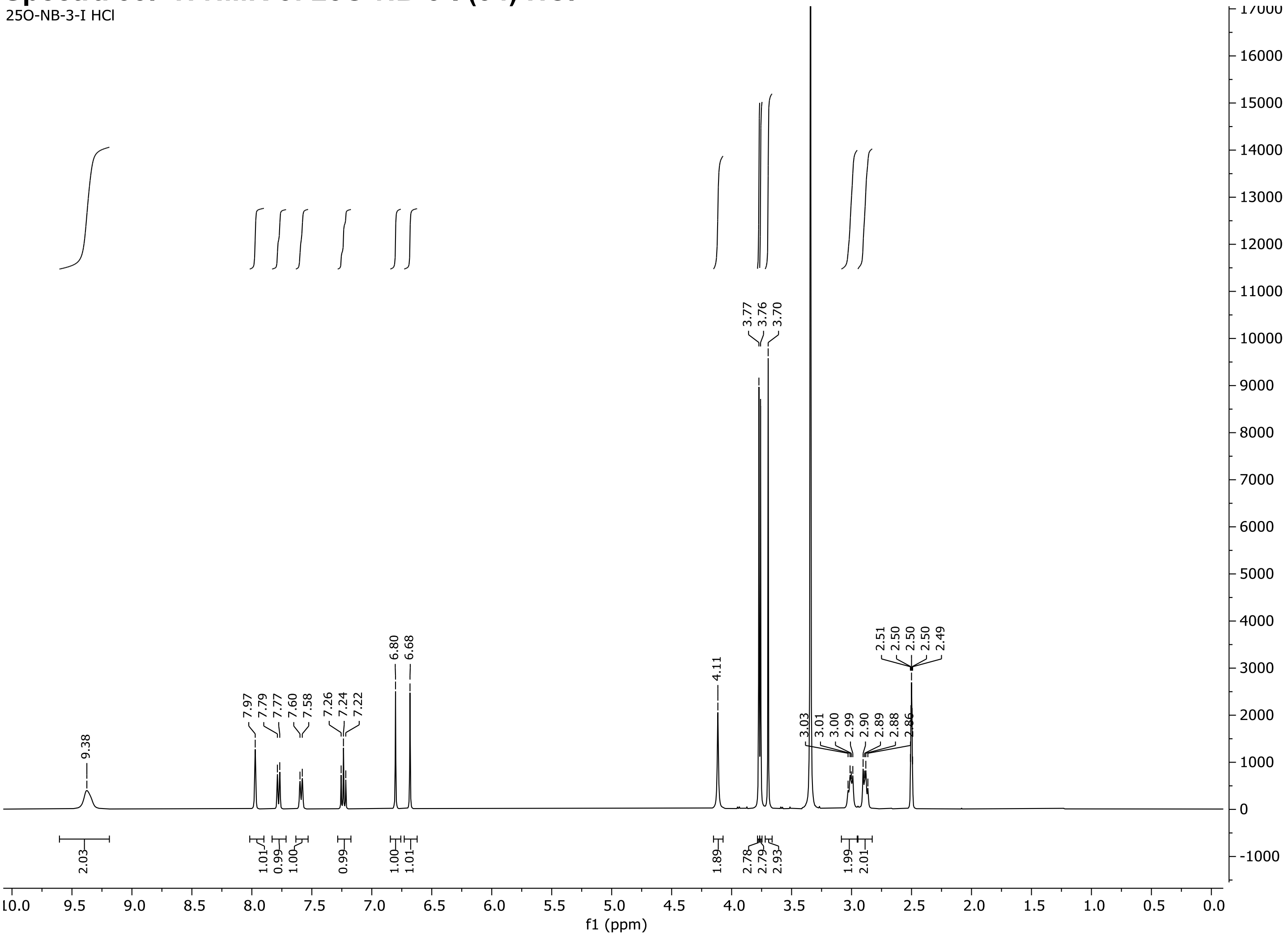

Spectra 34. ^{1}H NMR of 25O-NBPh-10'-OH (35) HCl

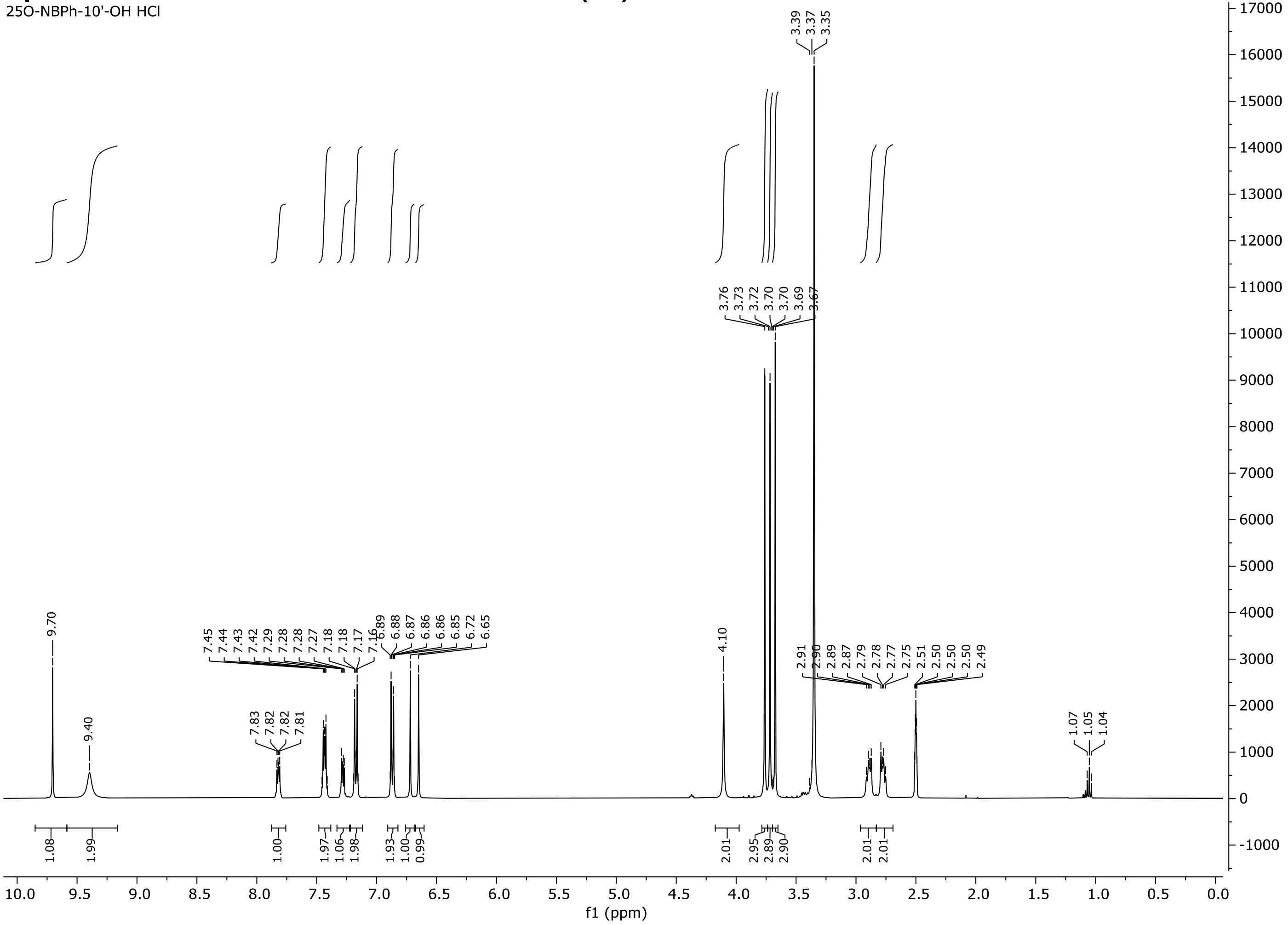

^{13}C NMR Spectra of Synthesized N-Benzyl Phenethylamines

Spectra 35. ^{13}C NMR of 25N-NB (2) HCl

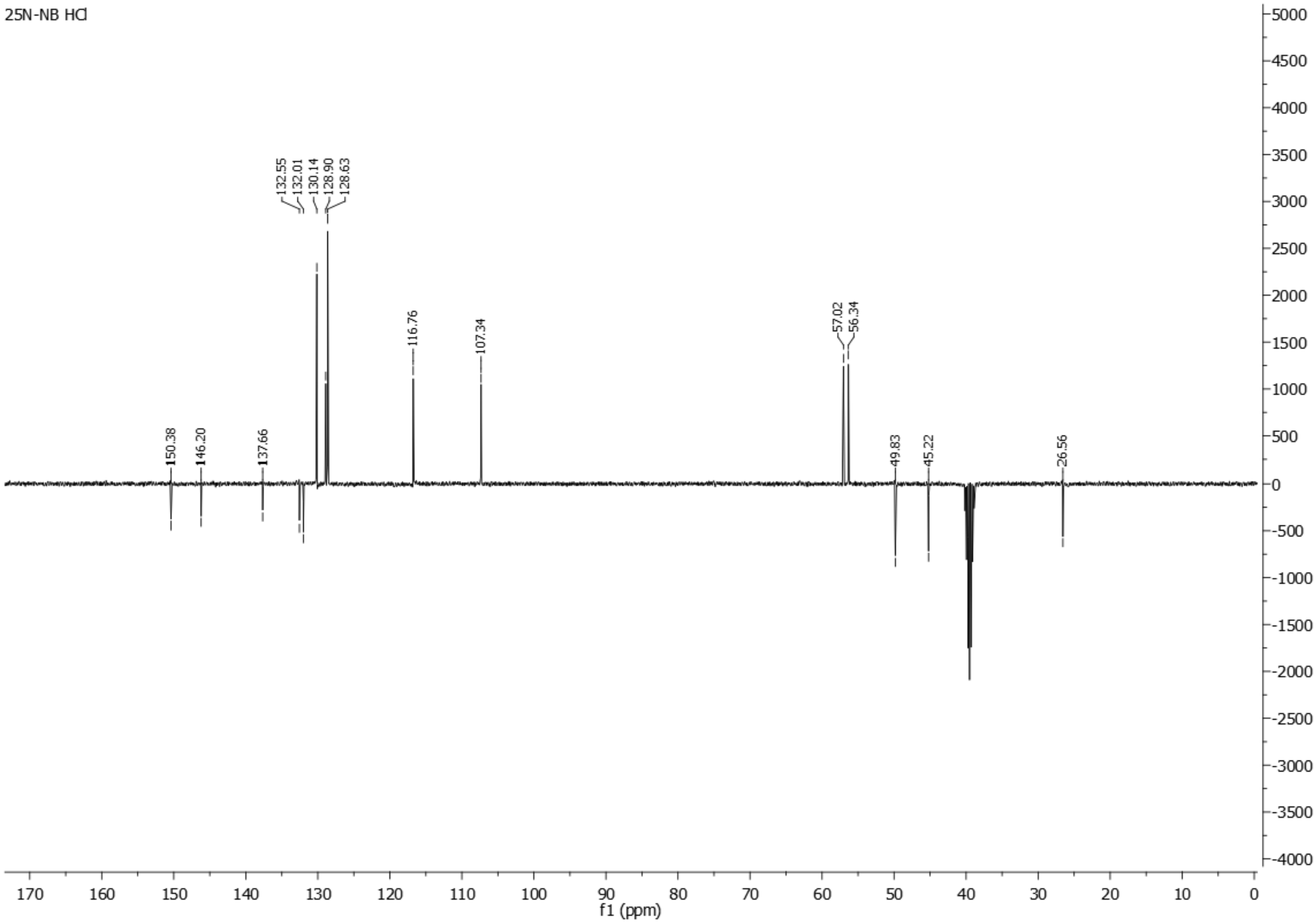

Spectra 36. ^{13}C NMR of 25N-NBOH (3) HCl

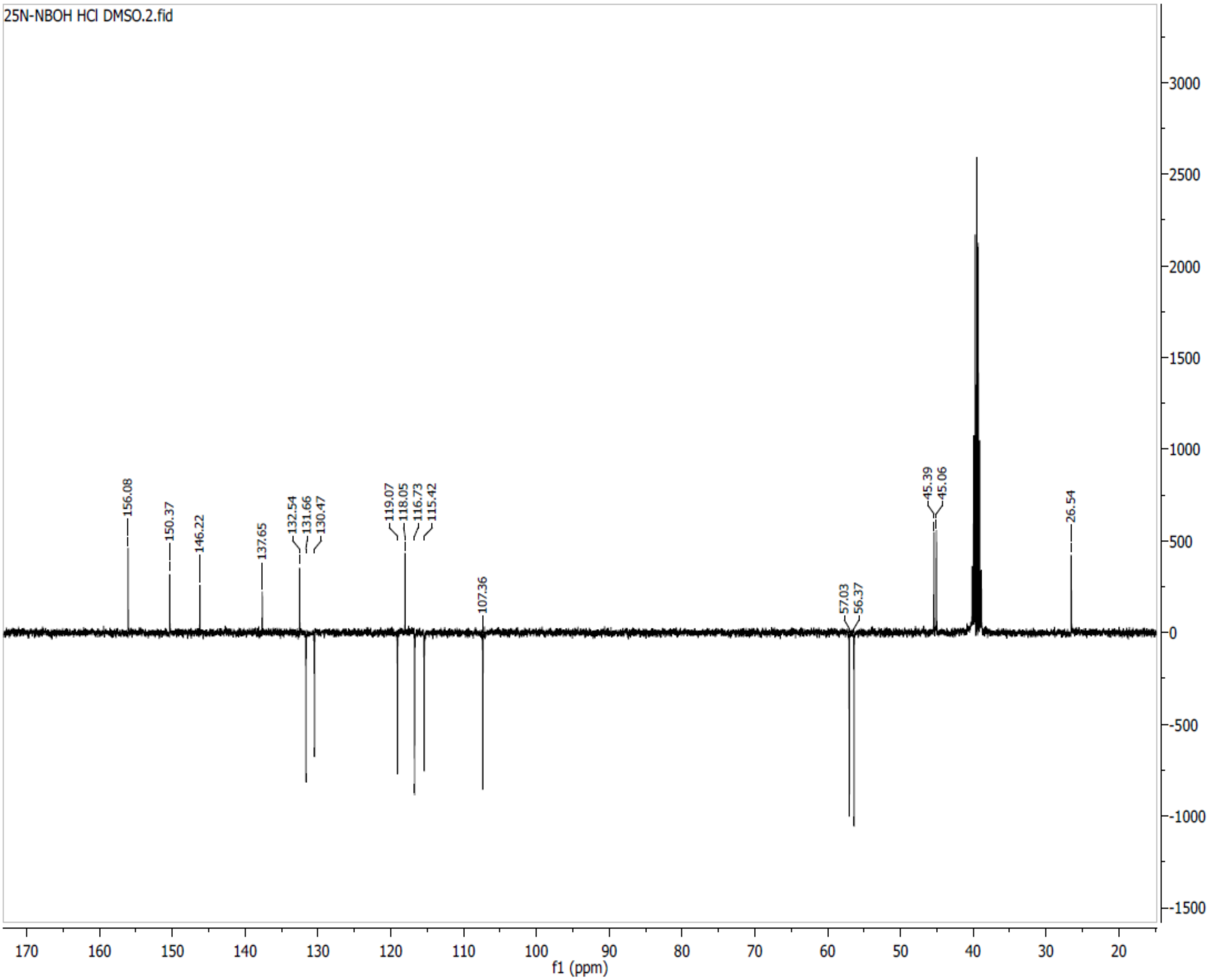

Spectra 37. ^{13}C NMR of 25N-NBOMe (4) HCl

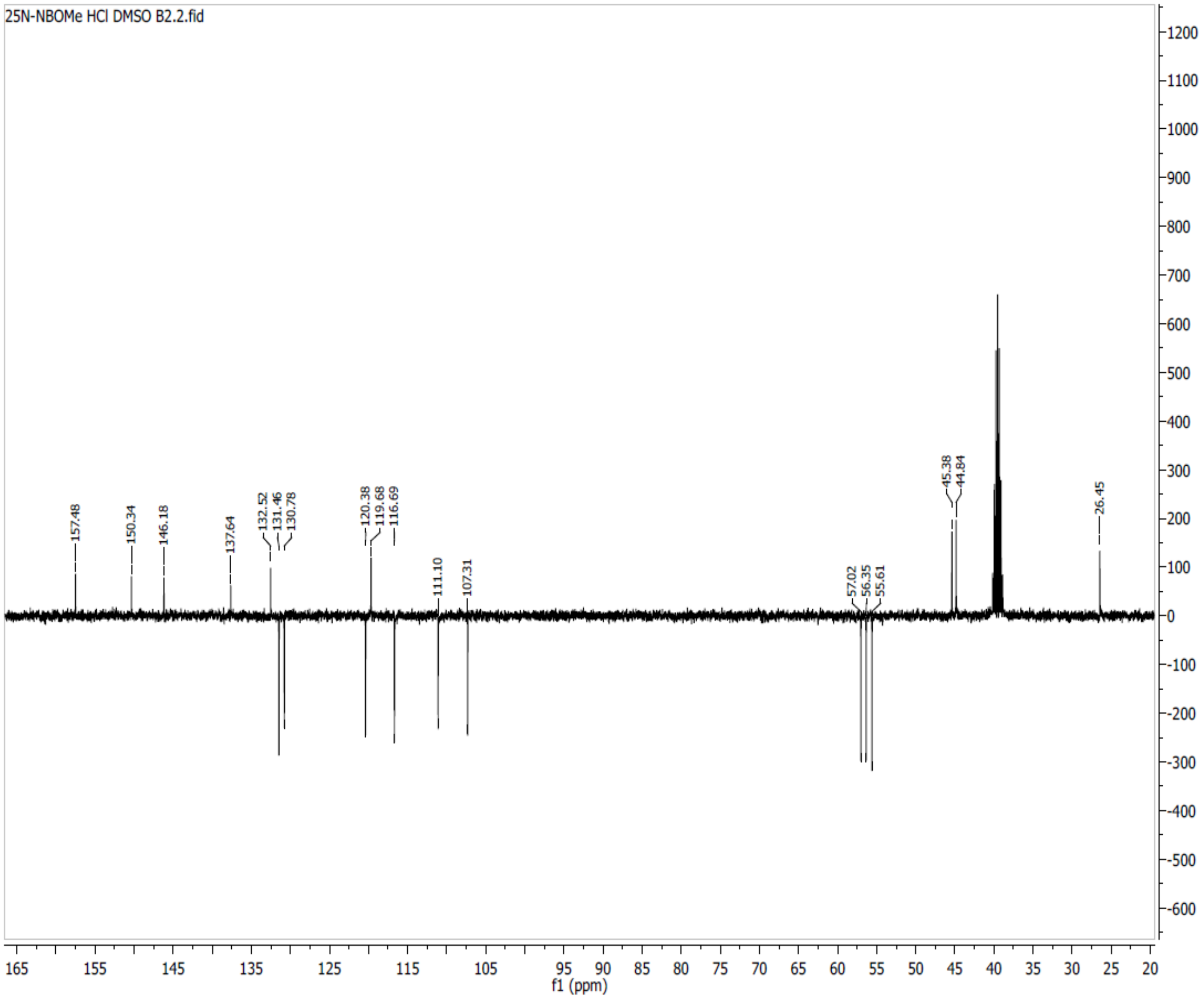

Spectra 38. ^{13}C NMR of 25N-NBOEt (5) HCl

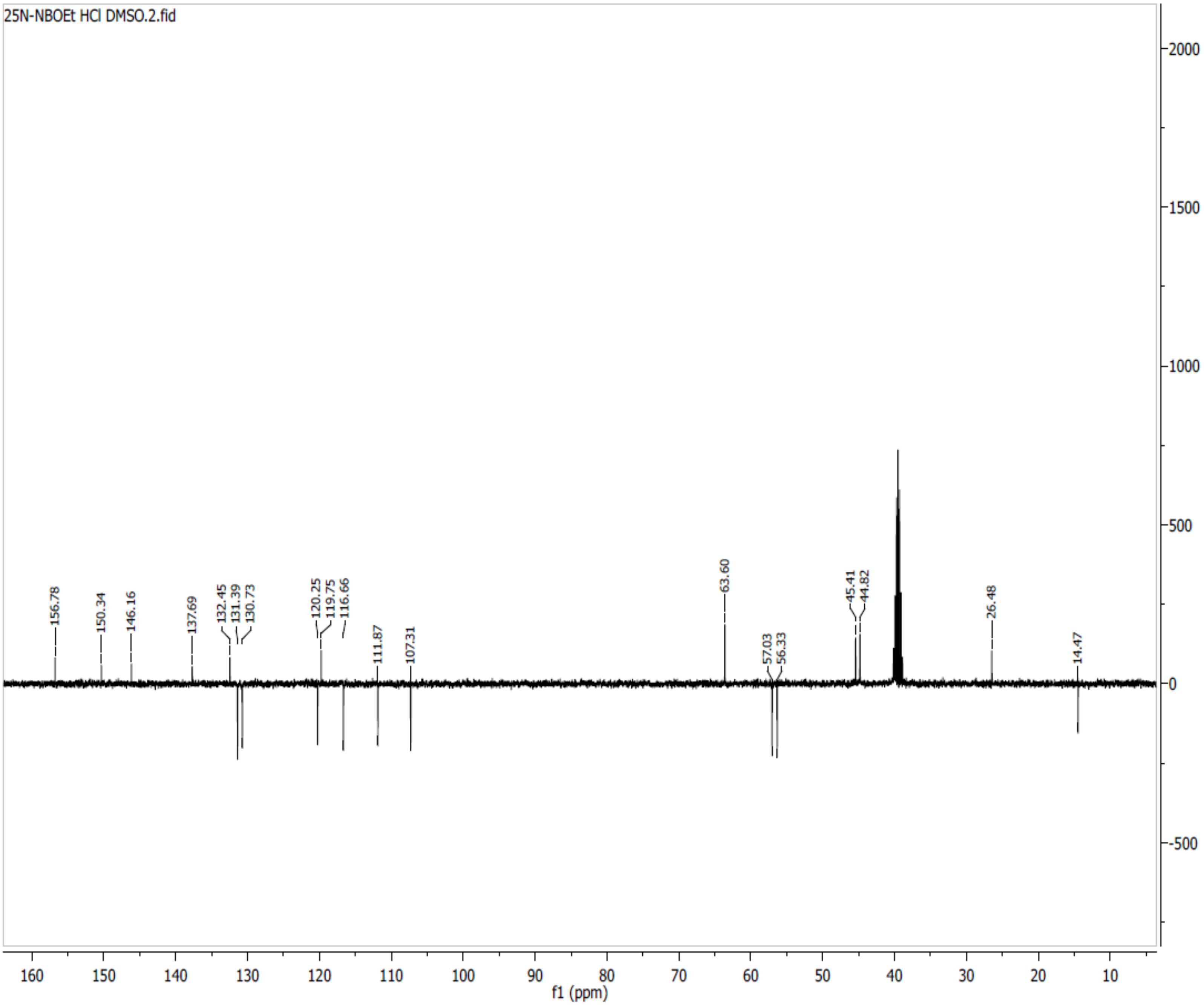

Spectra 39. ^{13}C NMR of 25N-NBMe (6) HCl

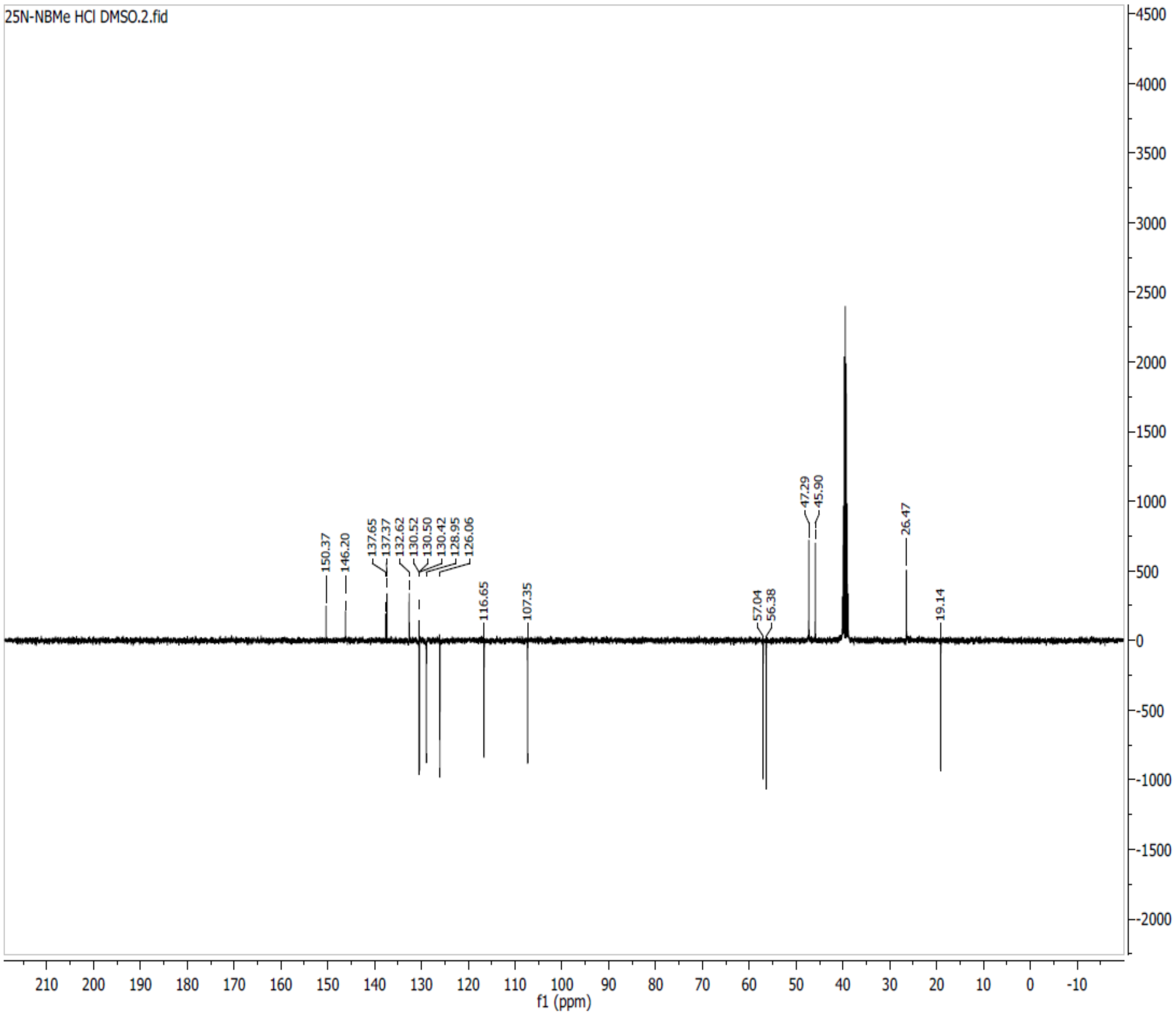

Spectra 40. ^{13}C NMR of 25N-NBF (7) HCl

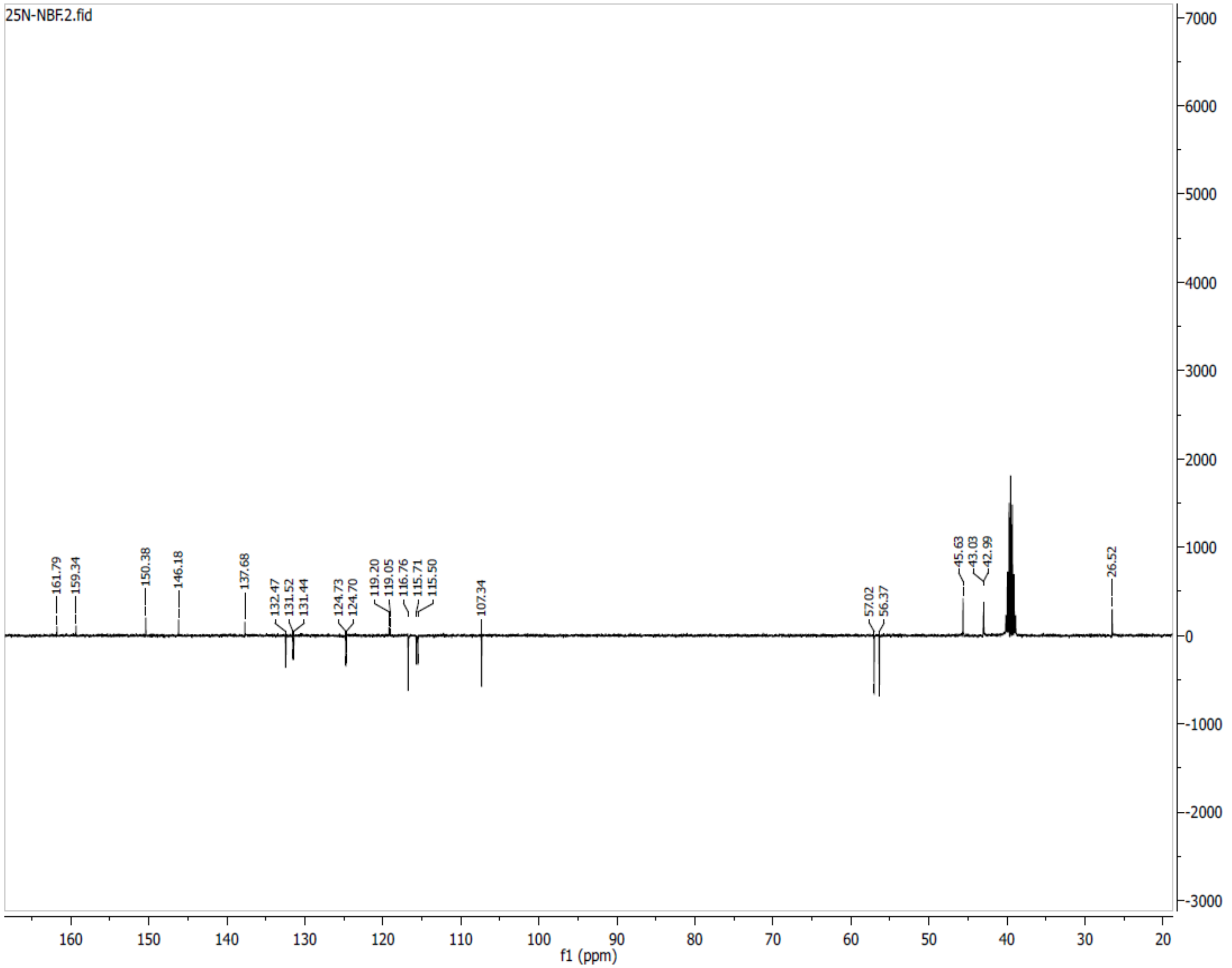

Spectra 41. ^{13}C NMR of 25N-NBCl (8) HCl

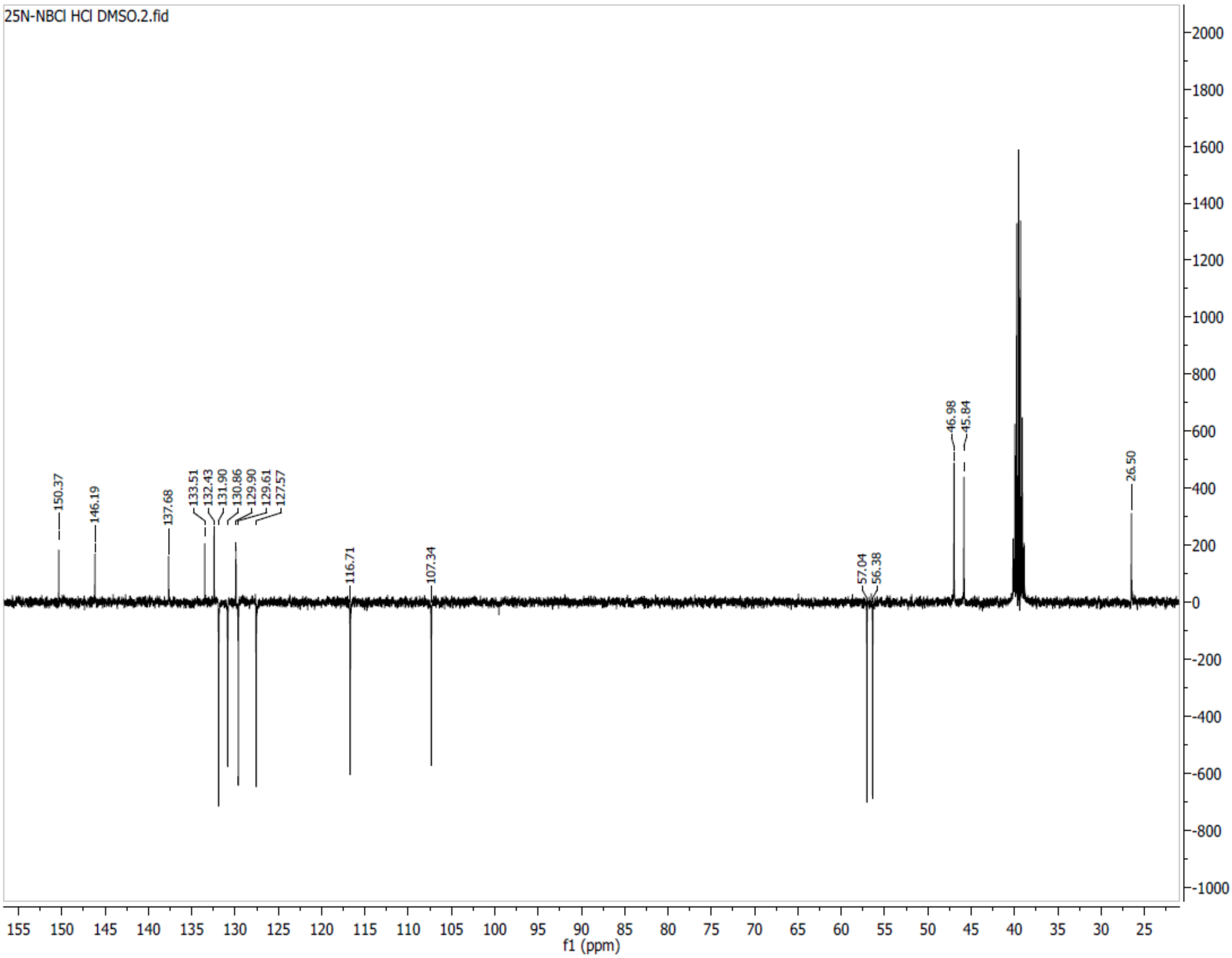

Spectra 42. ^{13}C NMR of 25N-NBBr (9) HCl

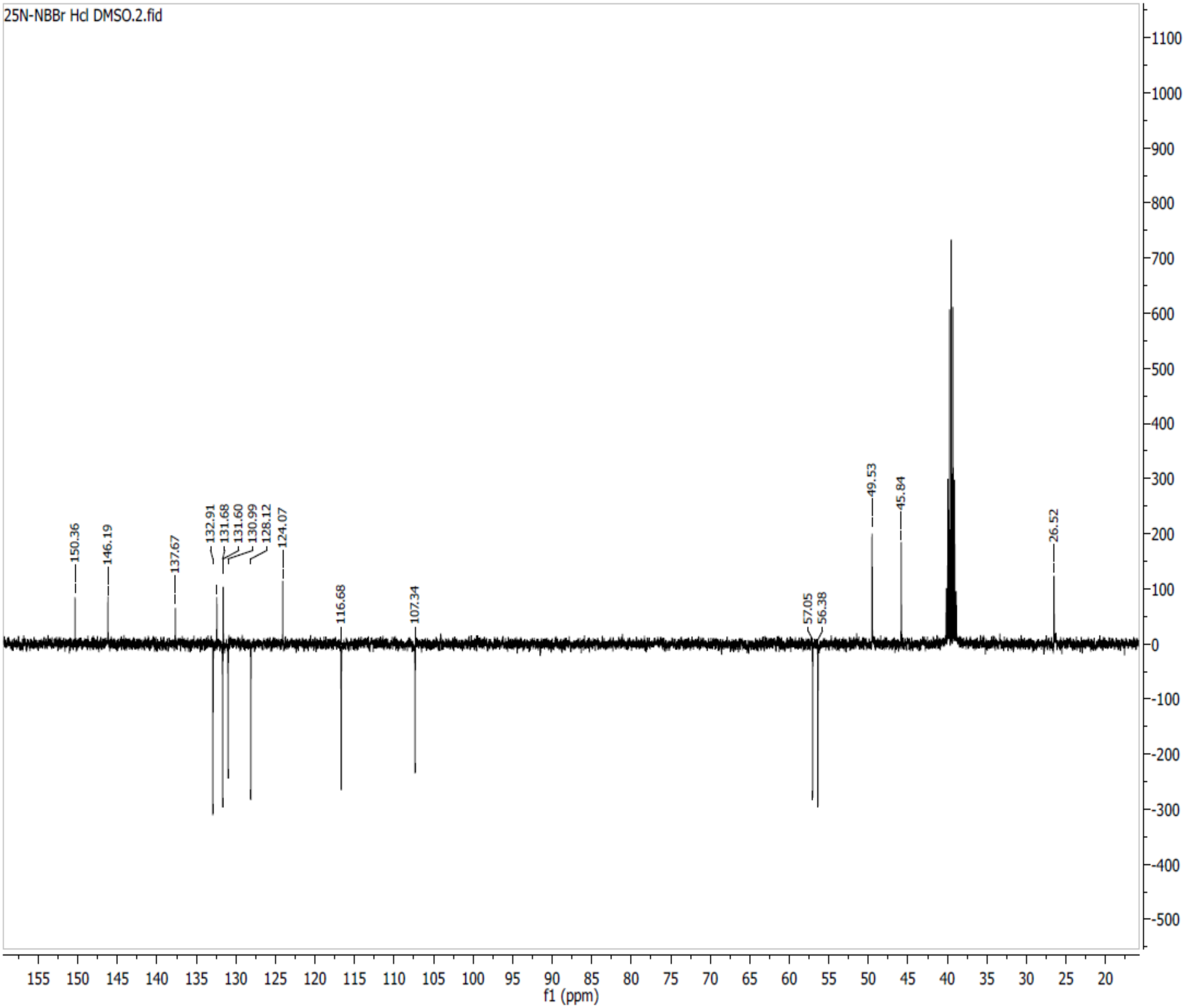

Spectra 43. ^{13}C NMR of 25N-NBI (10) HCl

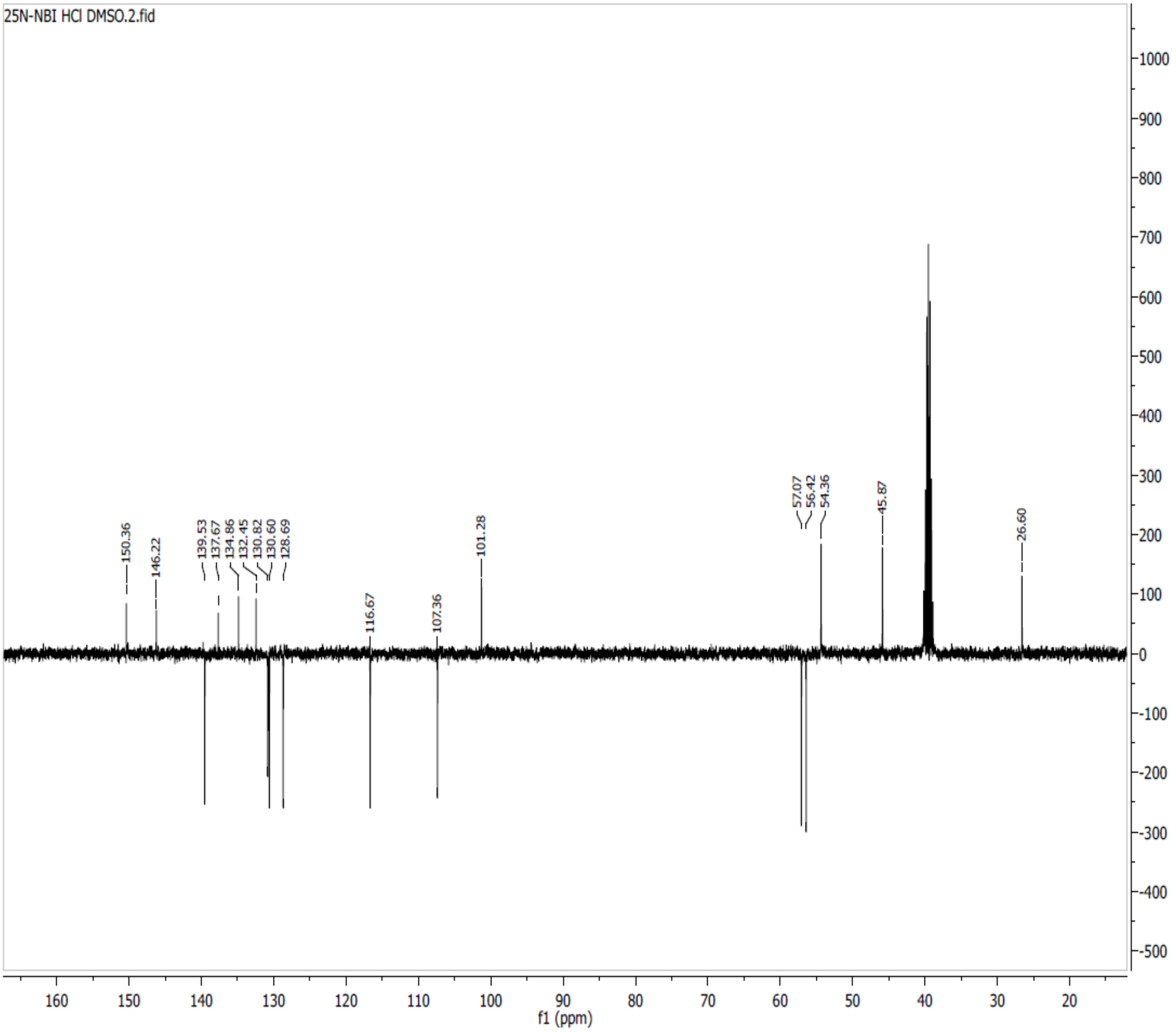

Spectra 44. ^{13}C NMR of 25N-NBOCF$_2$H (11) HCl

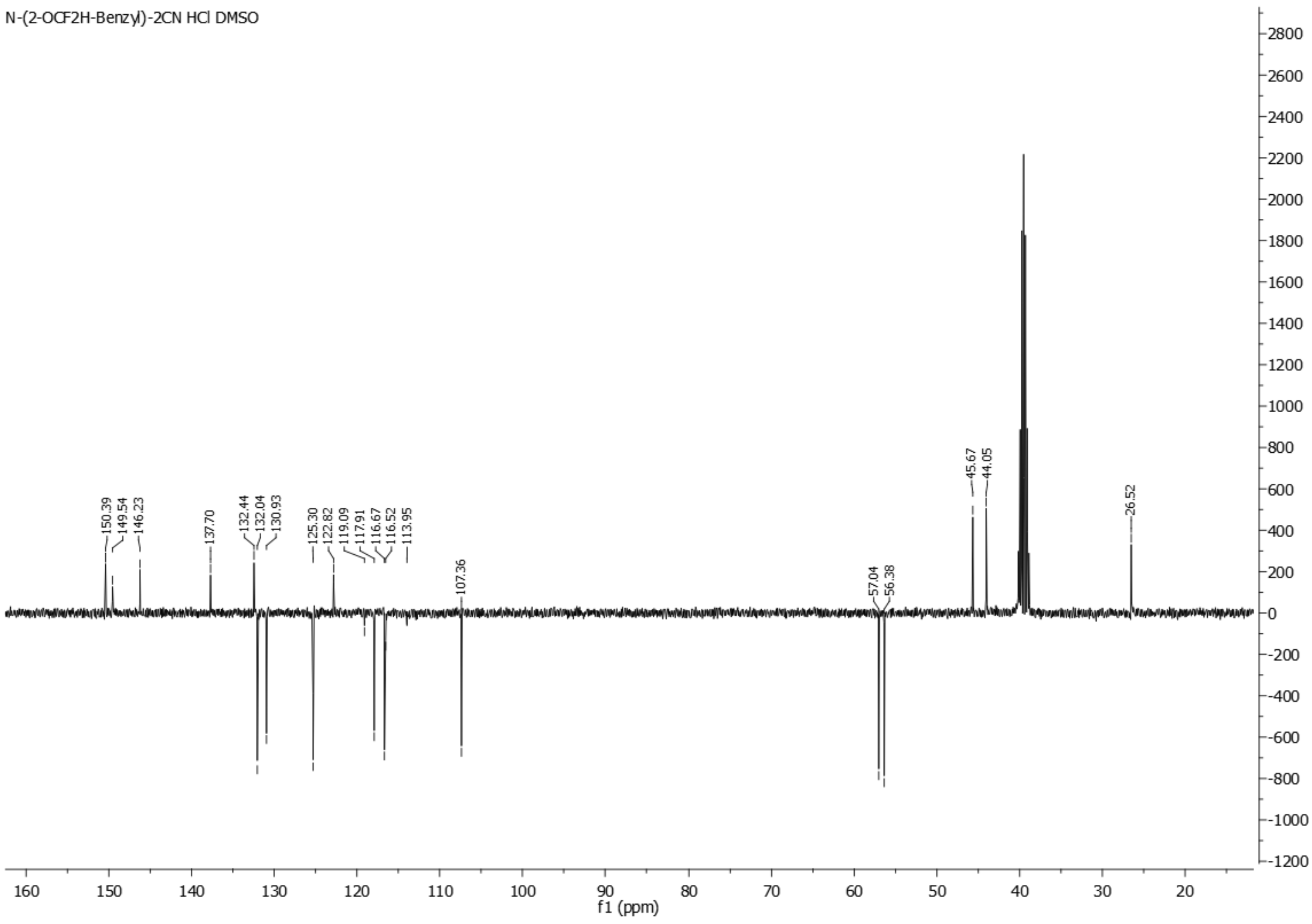

Spectra 45. ^{13}C NMR of 25N-NBOCF$_3$ (12) HCl

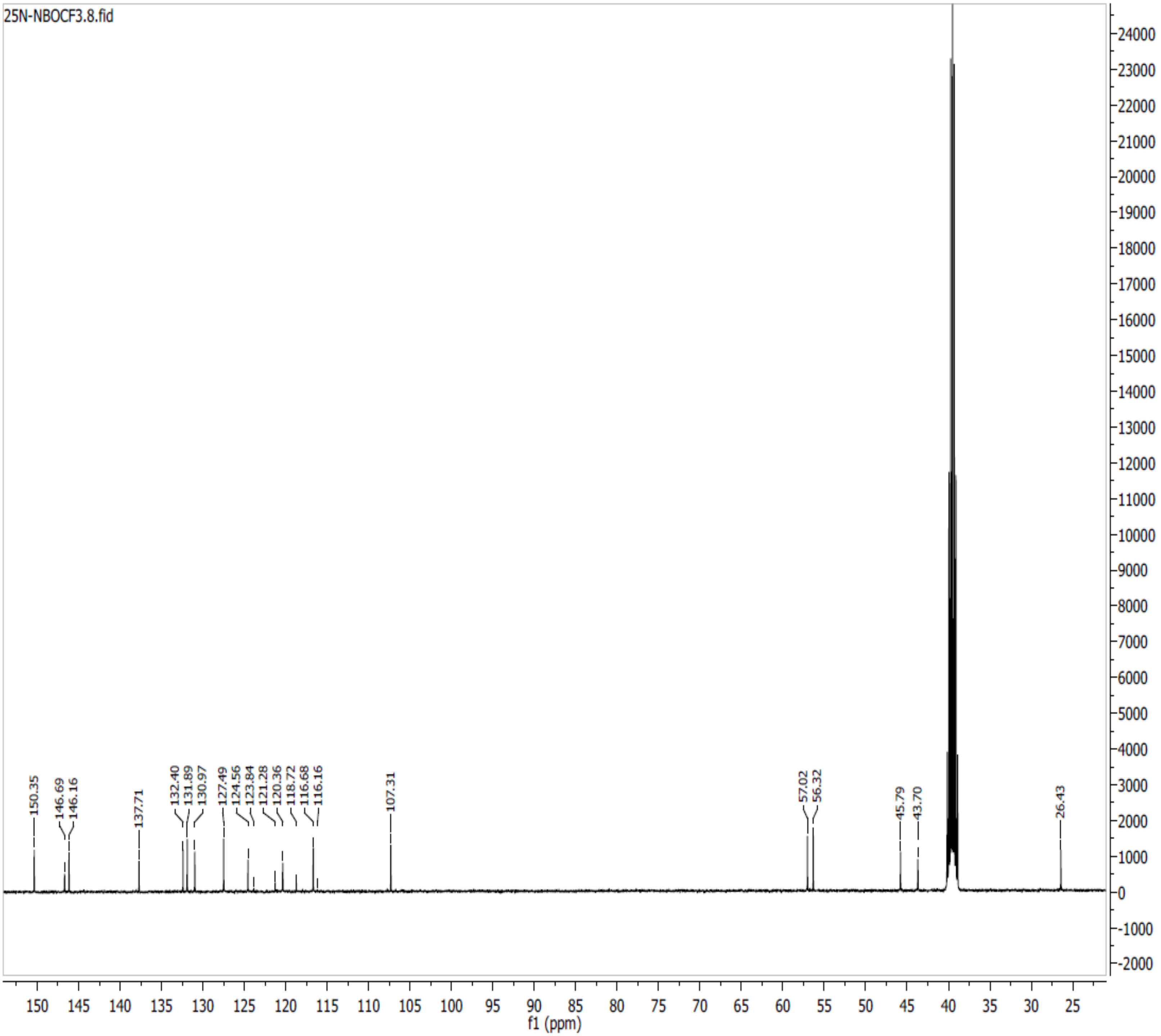

Spectra 46. ^{13}C NMR of 25N-NBMDF$_2$ (13) HCl

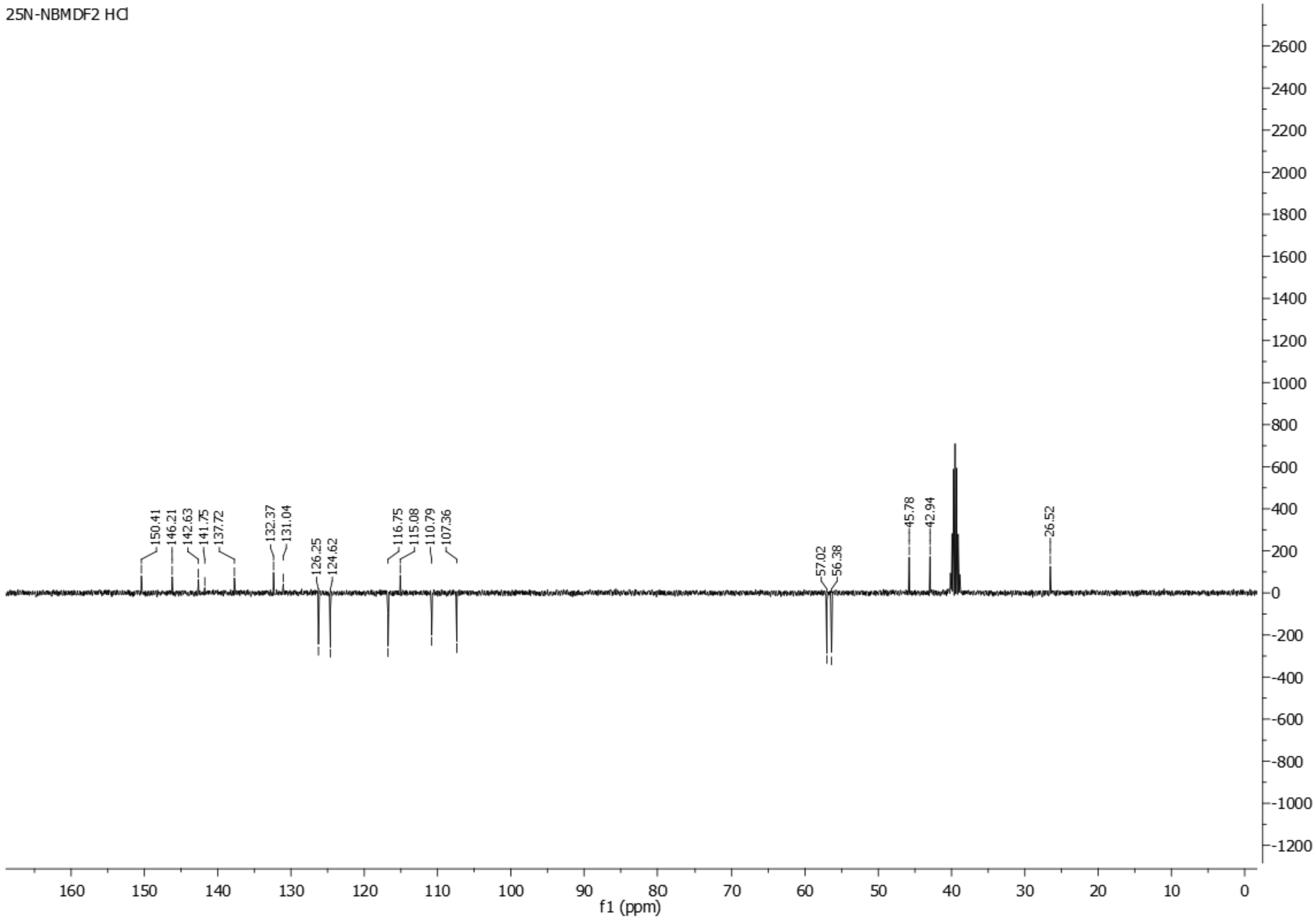

Spectra 47. ^{13}C NMR of 25N-NBCF$_3$ (14) HCl

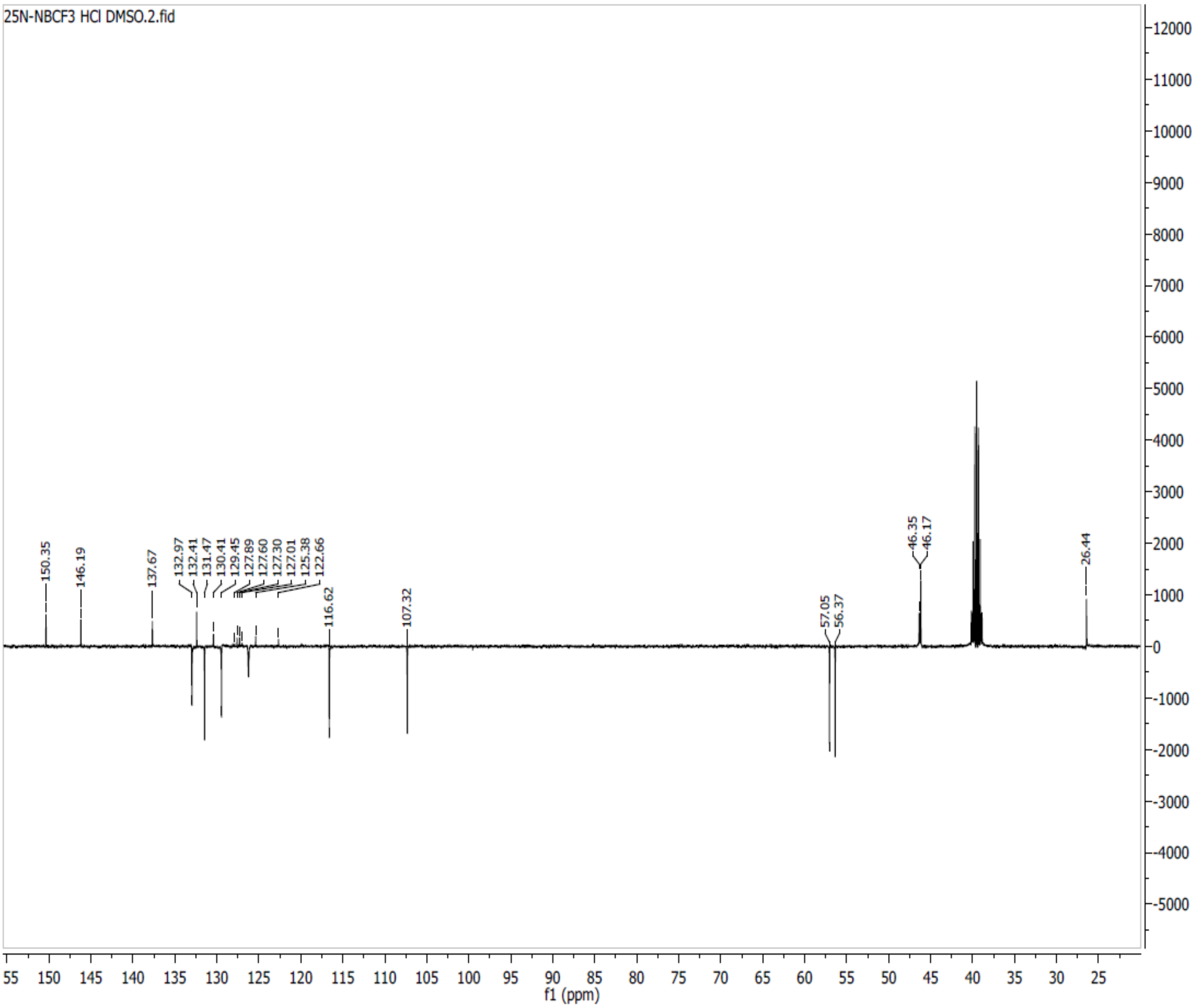

Spectra 48. ^{13}C NMR of 25N-NBNO$_2$ (15) HCl

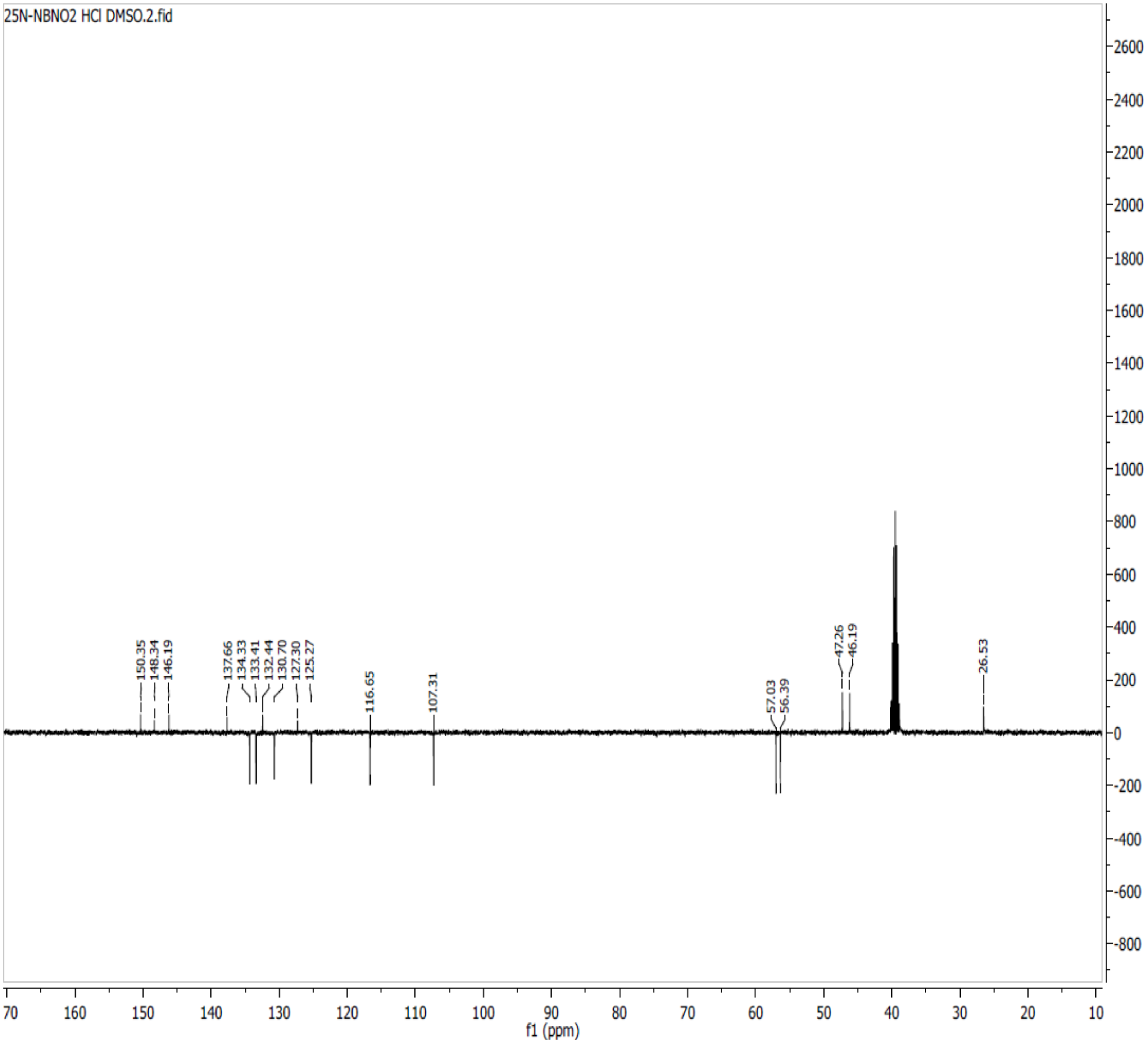

Spectra 49. ^{13}C NMR of 25N-N1-Nap (16) HCl

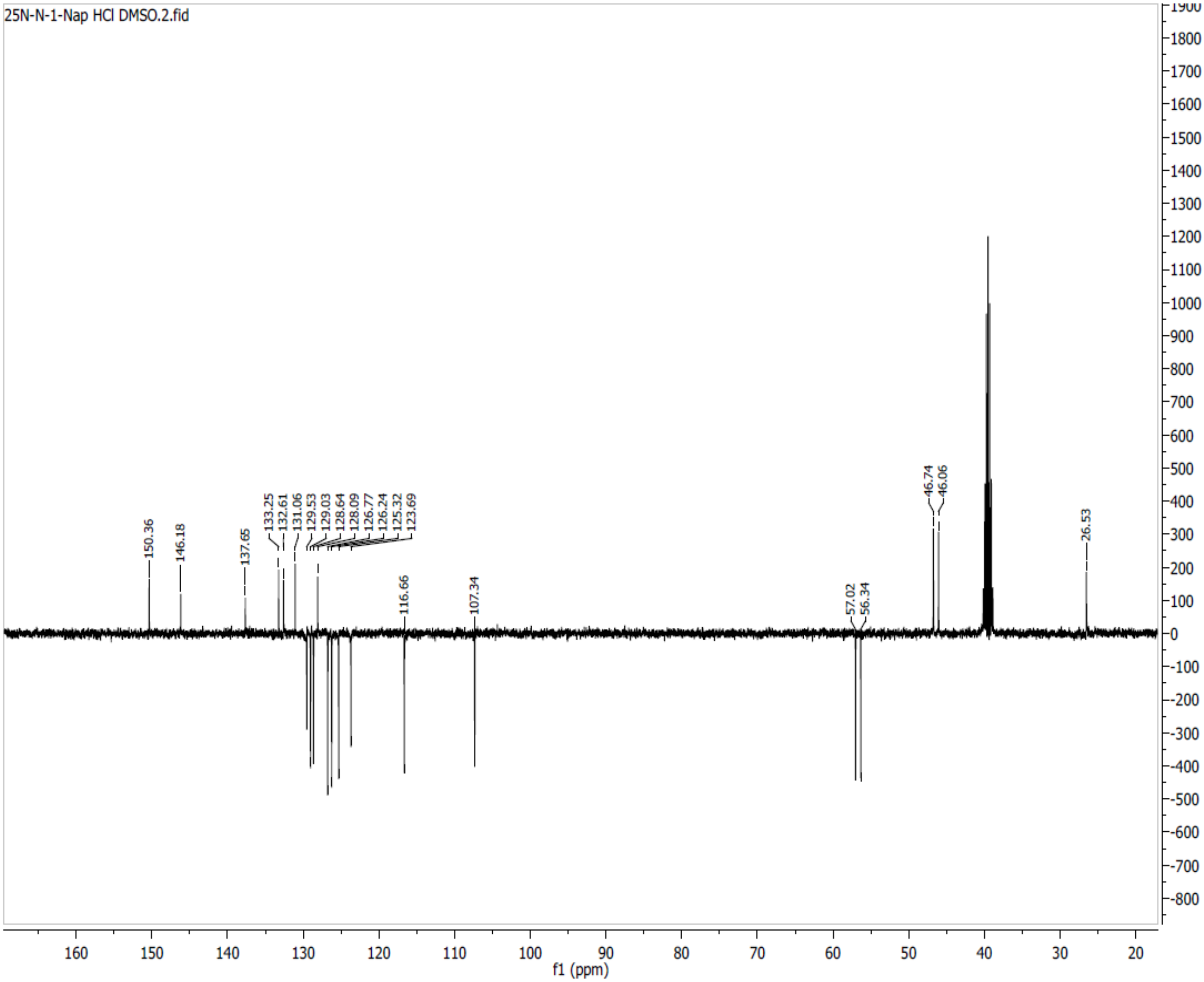

Spectra 50. ^{13}C NMR of 25N-NBPh (17) HCl

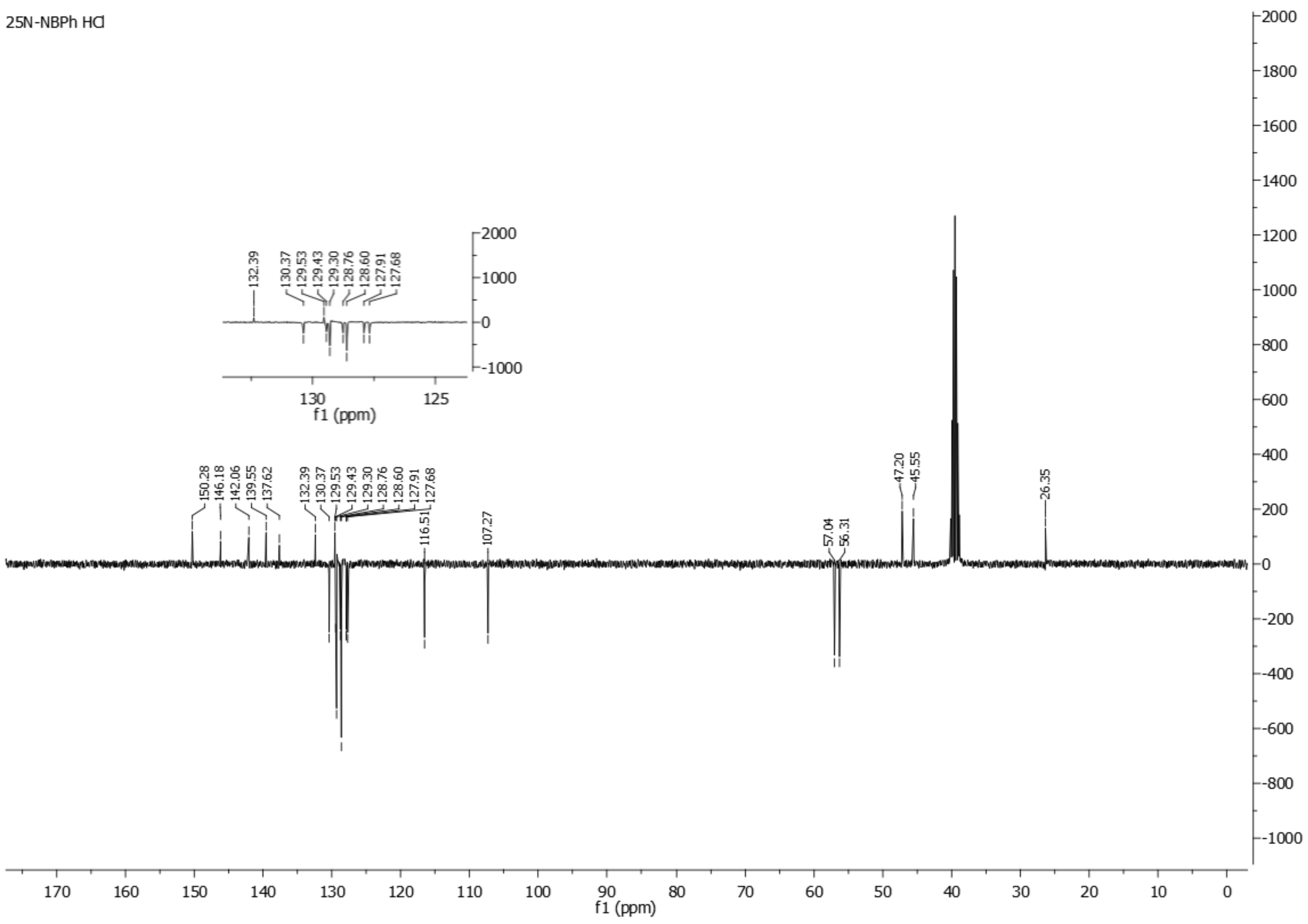

Spectra 51. ^{13}C NMR of 25N-NB-2-OH-3-Me (18) HCl

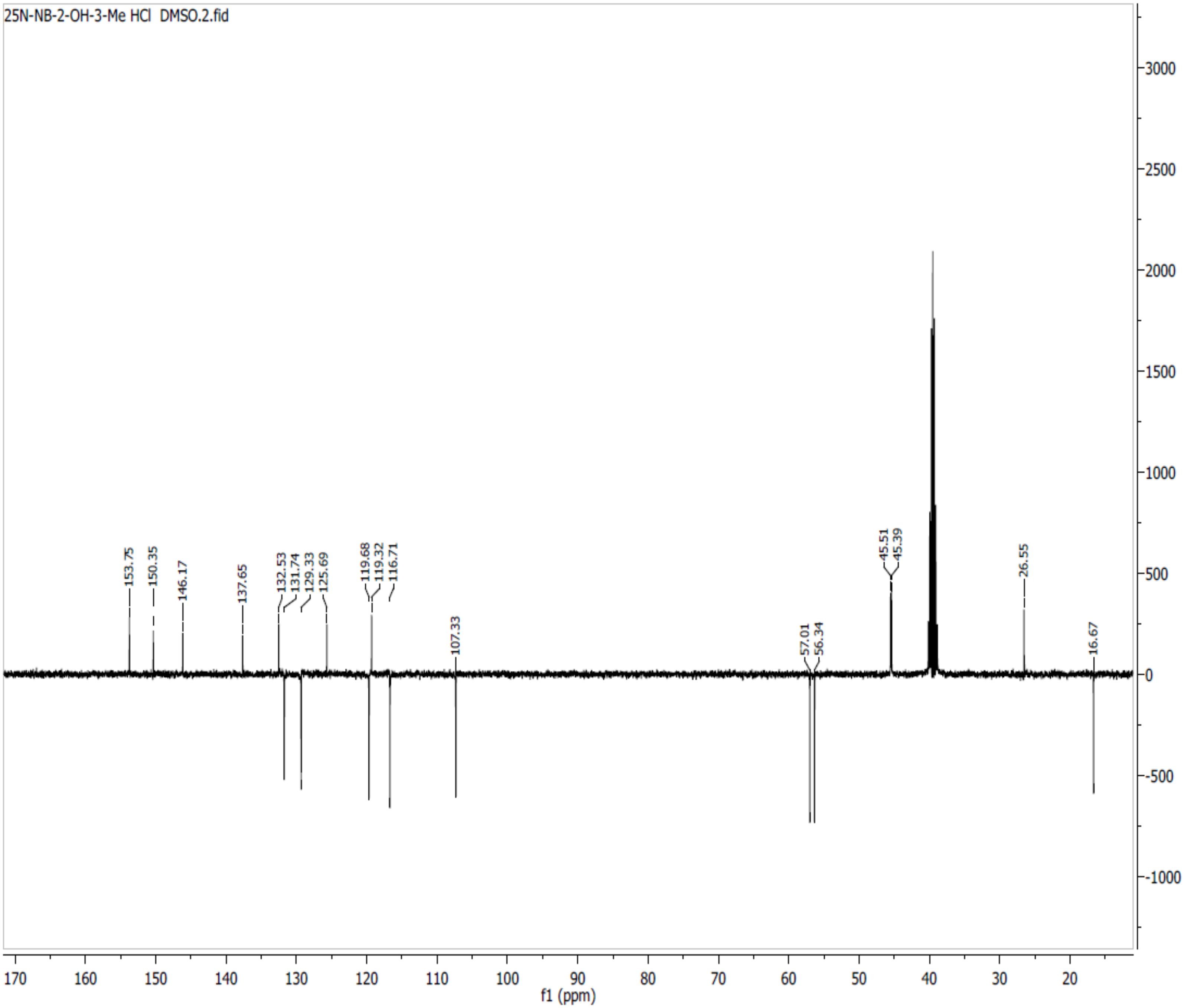

Spectra 52. ^{13}C NMR of 25N-NB-2-MeO-3-F (19) HCl

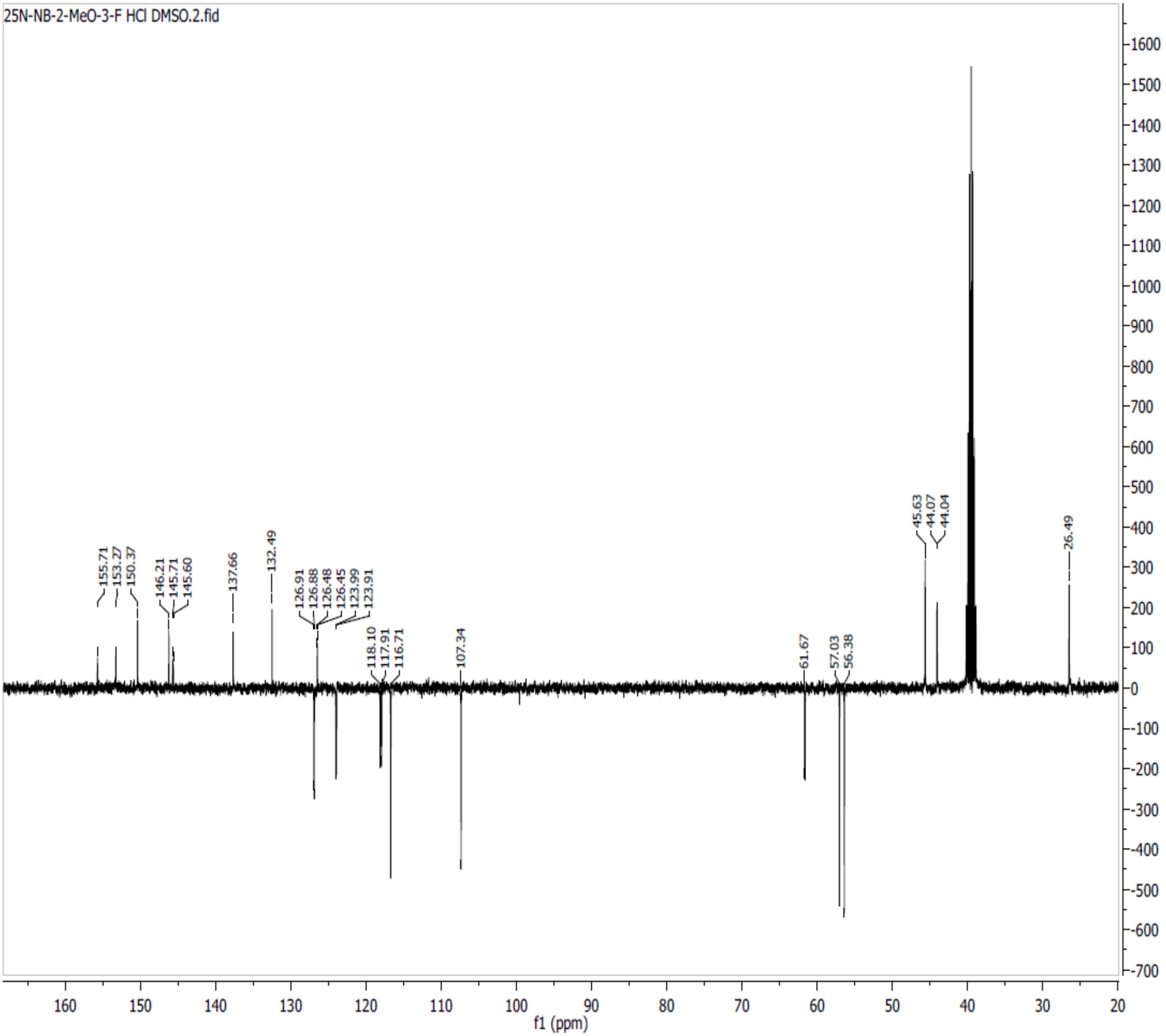

Spectra 53. ^{13}C NMR of 25N-NB-2,5-DiMeO (20) HCl

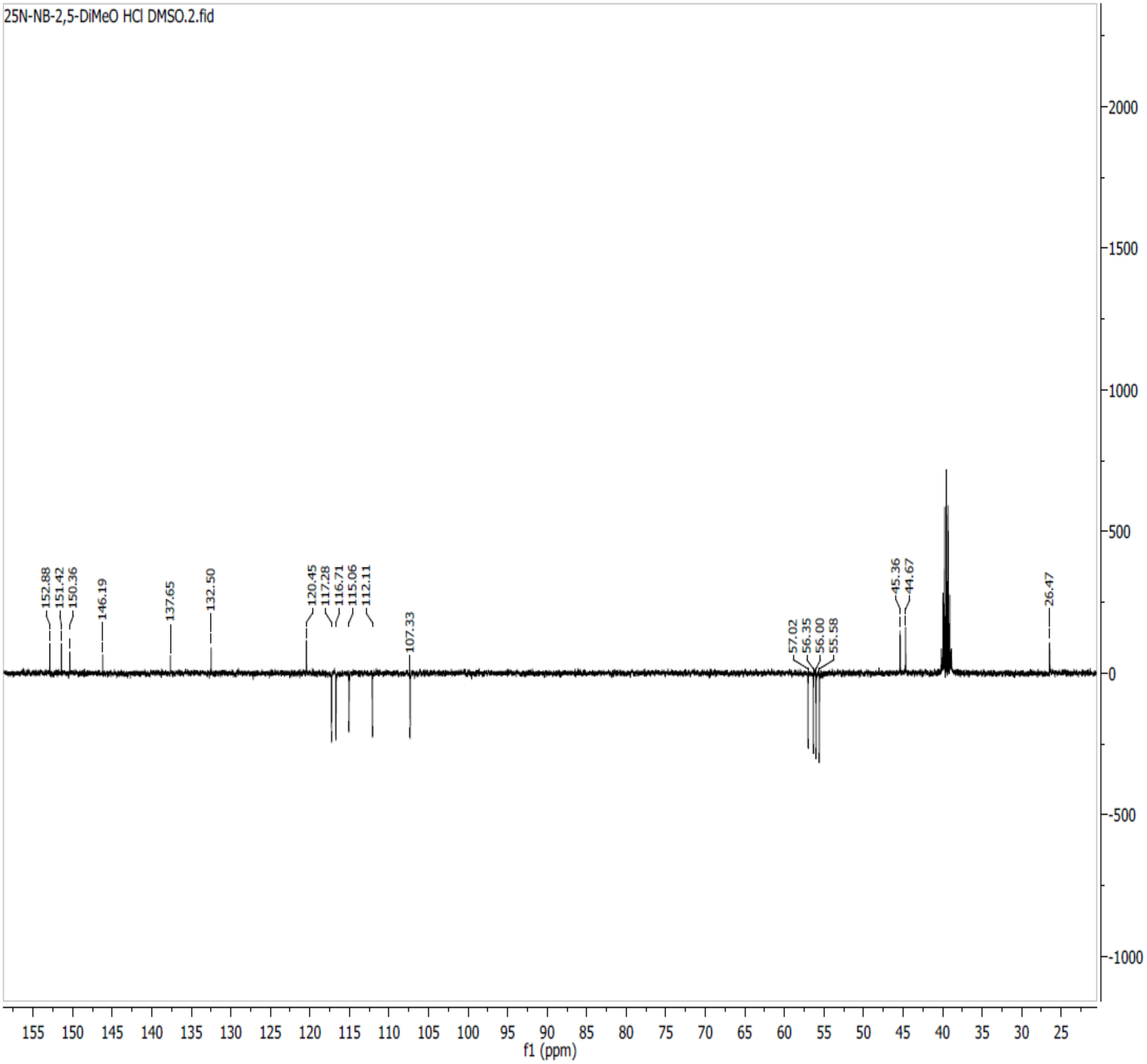

Spectra 54. ^{13}C NMR of 25N-NB-3-OH (21) HCl

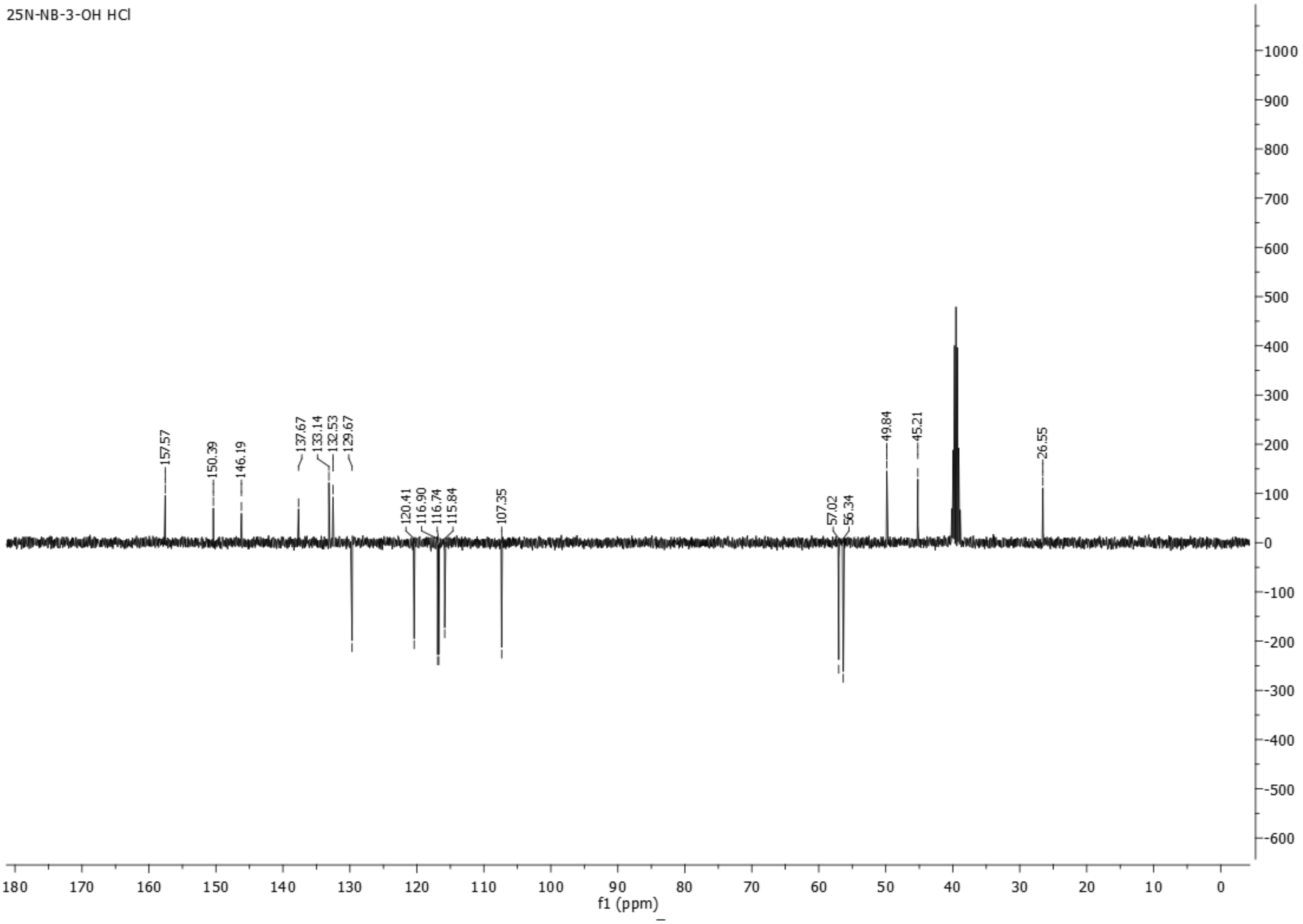

Spectra 55. ^{13}C NMR of 25N-NB-3-Me (22) HCl

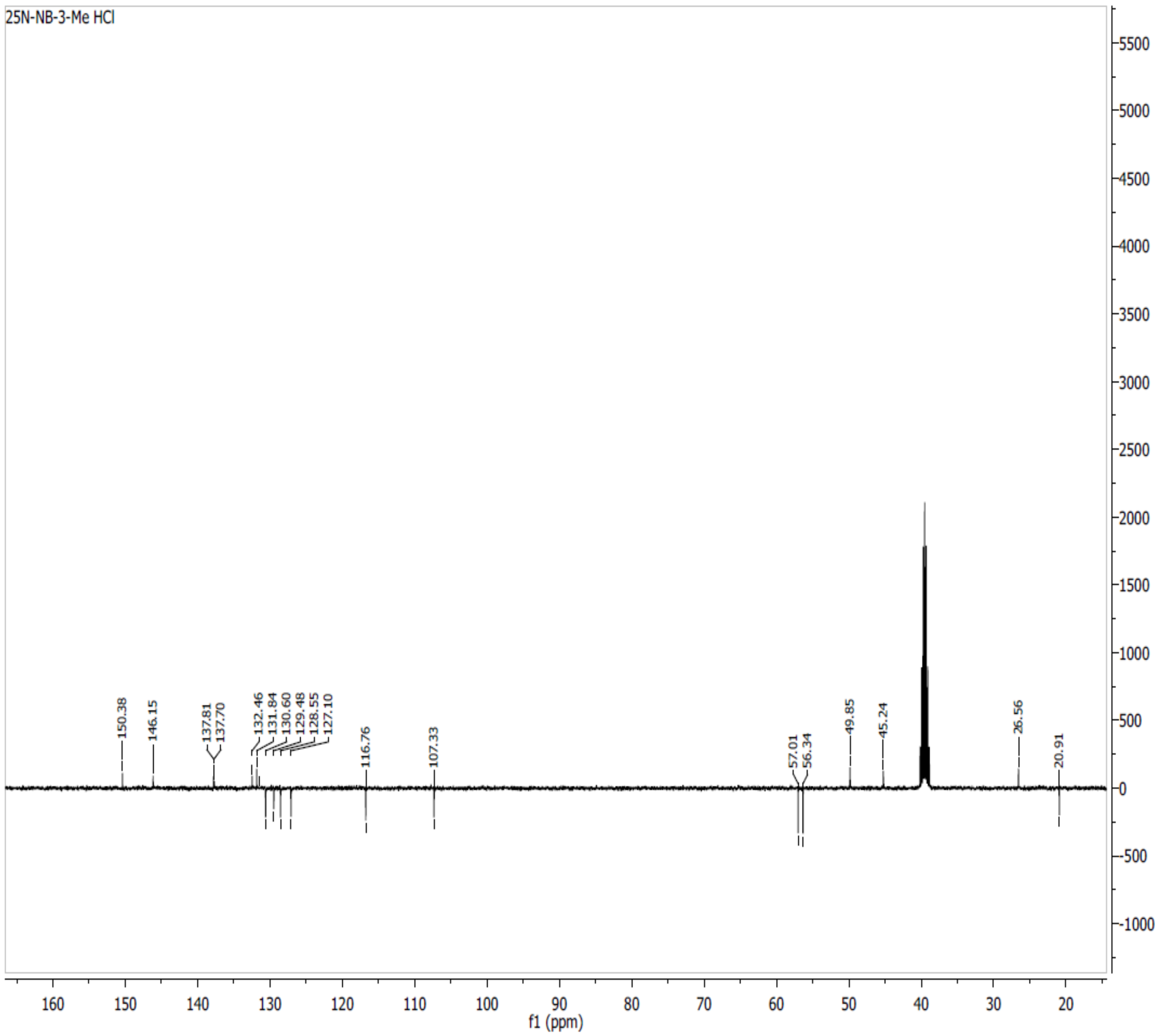

Spectra 56. ^{13}C NMR of 25N-NB-4-Me (23) HCl

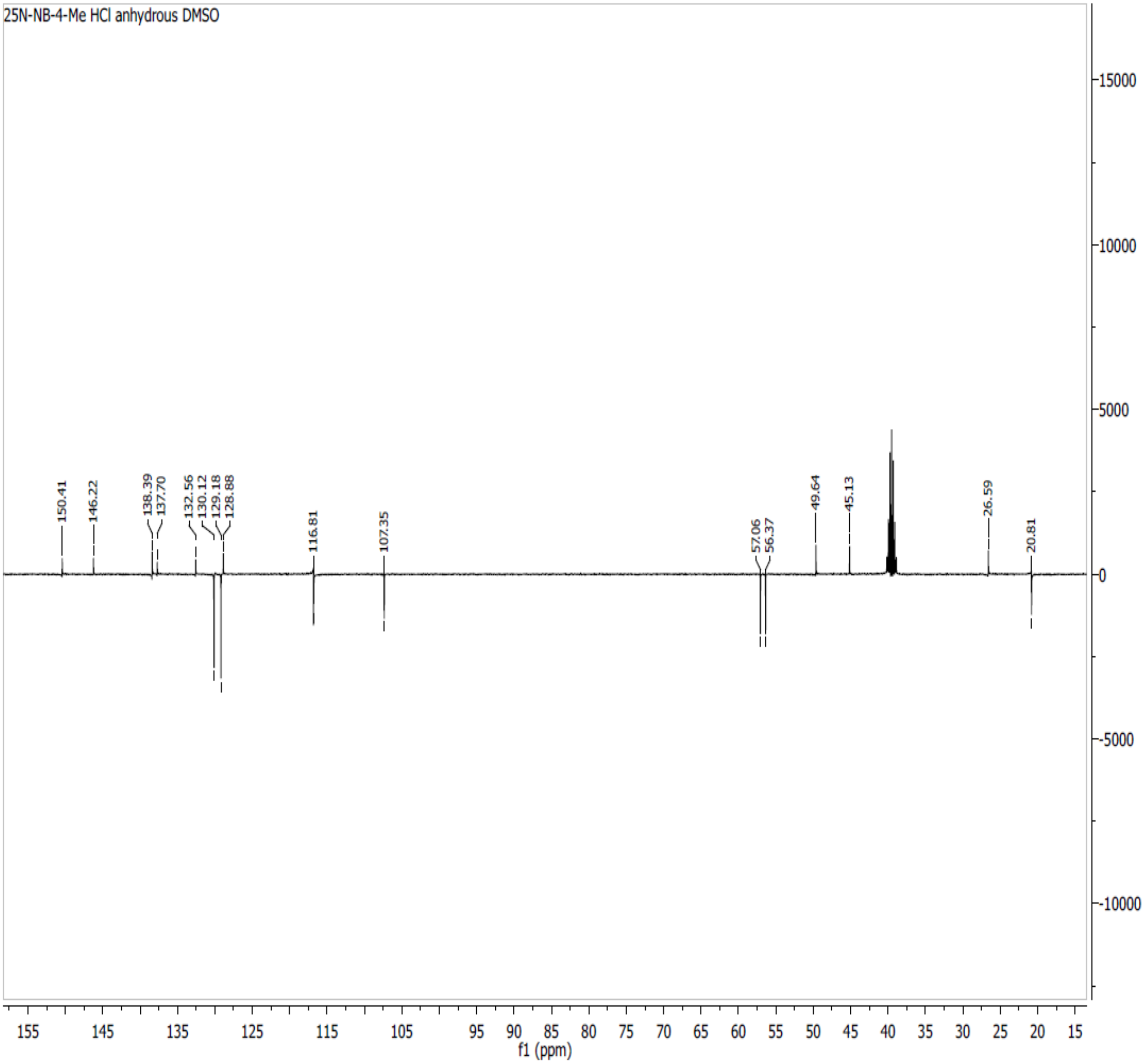

Spectra 57. ^{13}C NMR of 25N-NB-3-F (24) HCl

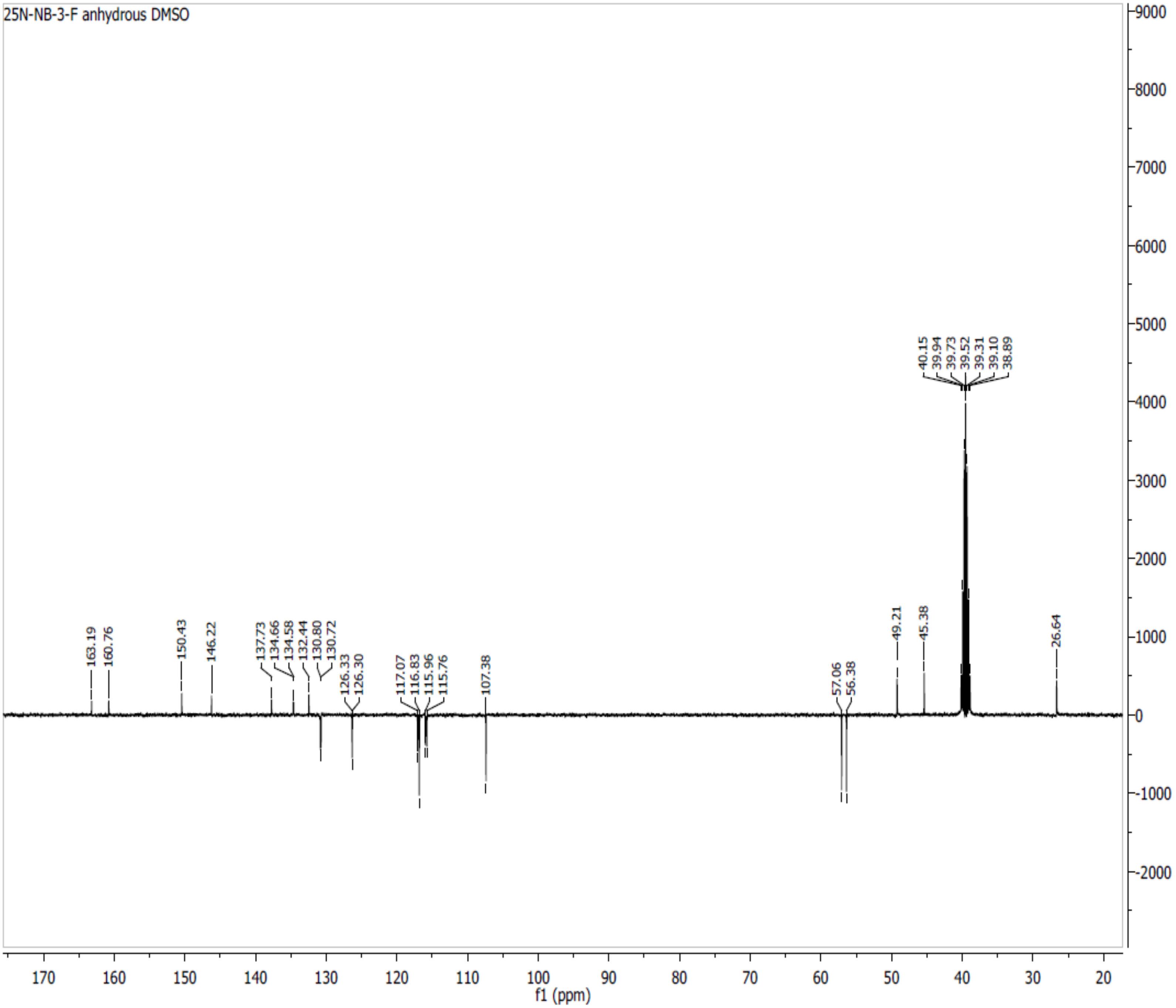

Spectra 58. ^{13}C NMR of 25N-NB-4-F (25) HCl

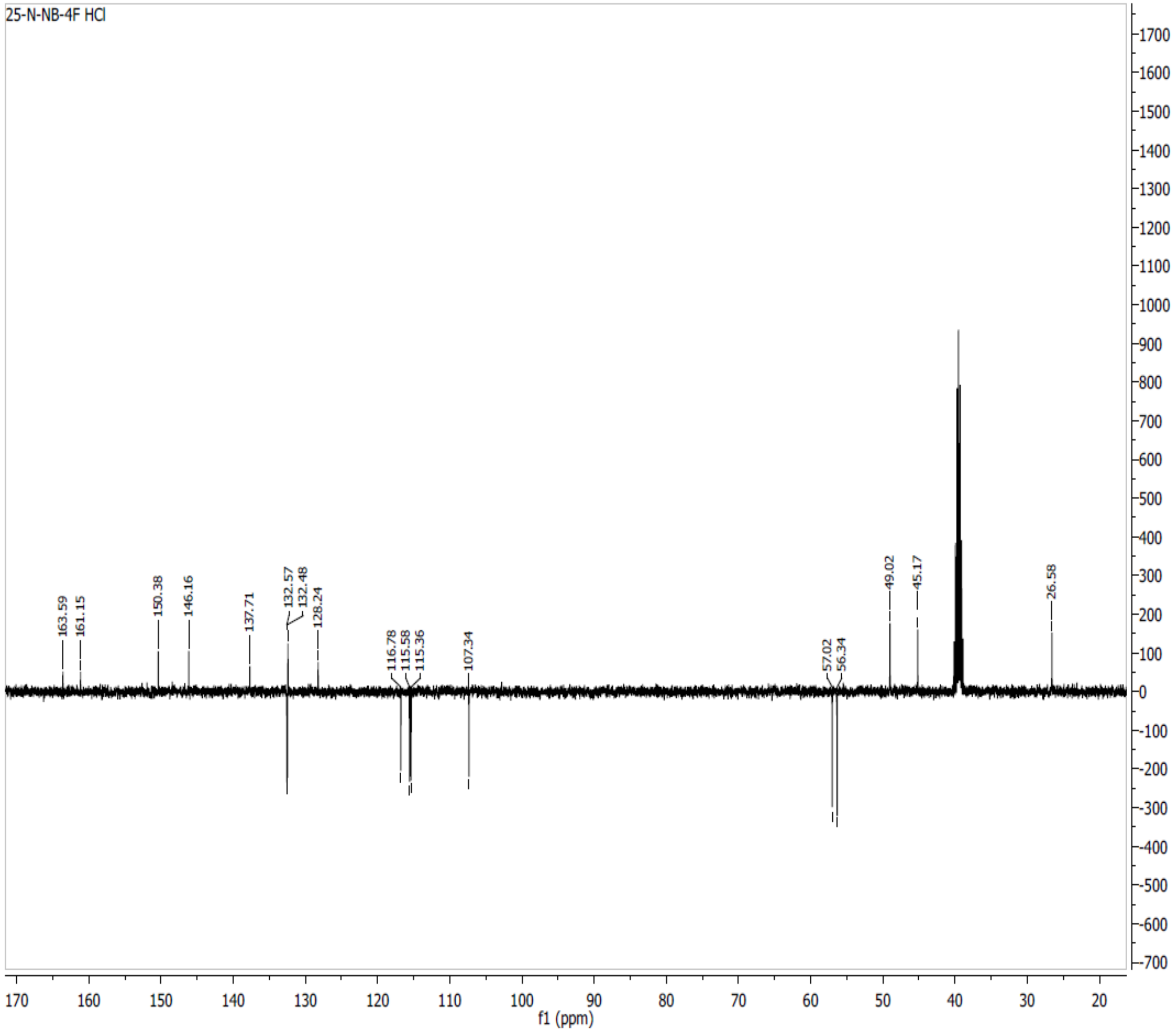

Spectra 59. ^{13}C NMR of 25D-N1-Nap (26) HCl

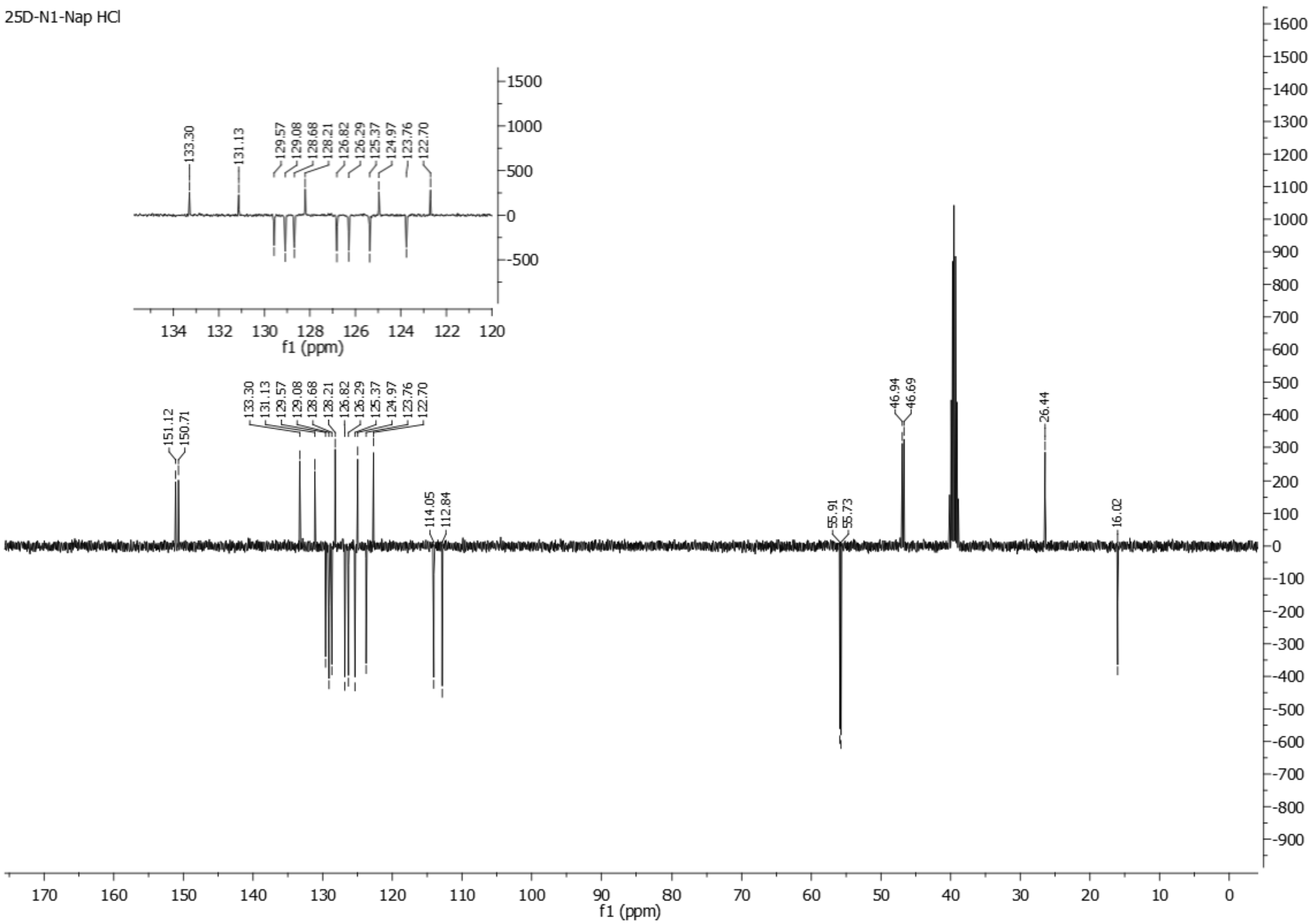

Spectra 60. ^{13}C NMR of 25D-NBPh (27) HCl

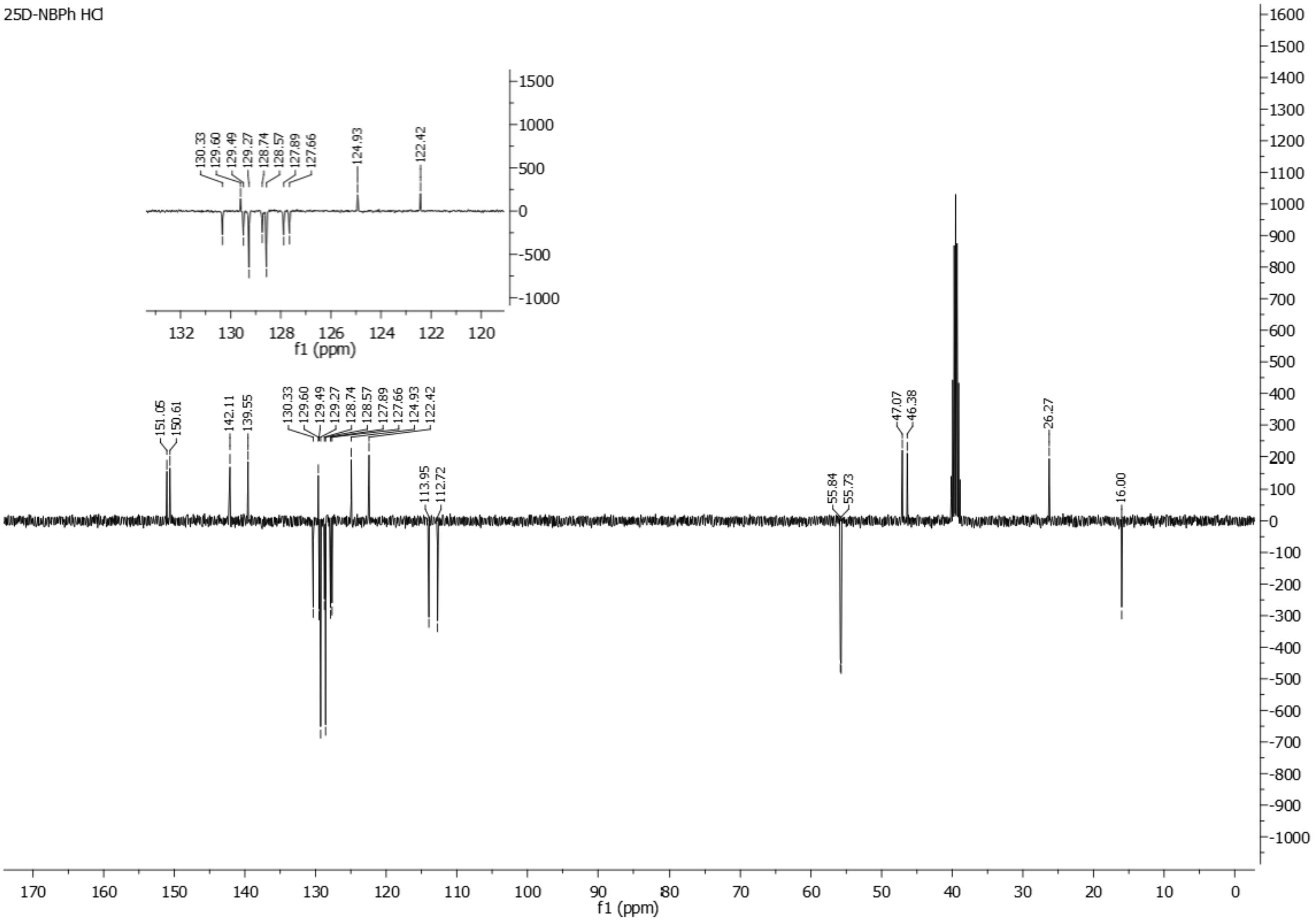

Spectra 61. ^{13}C NMR of 25O-N1-Nap (28) HCl

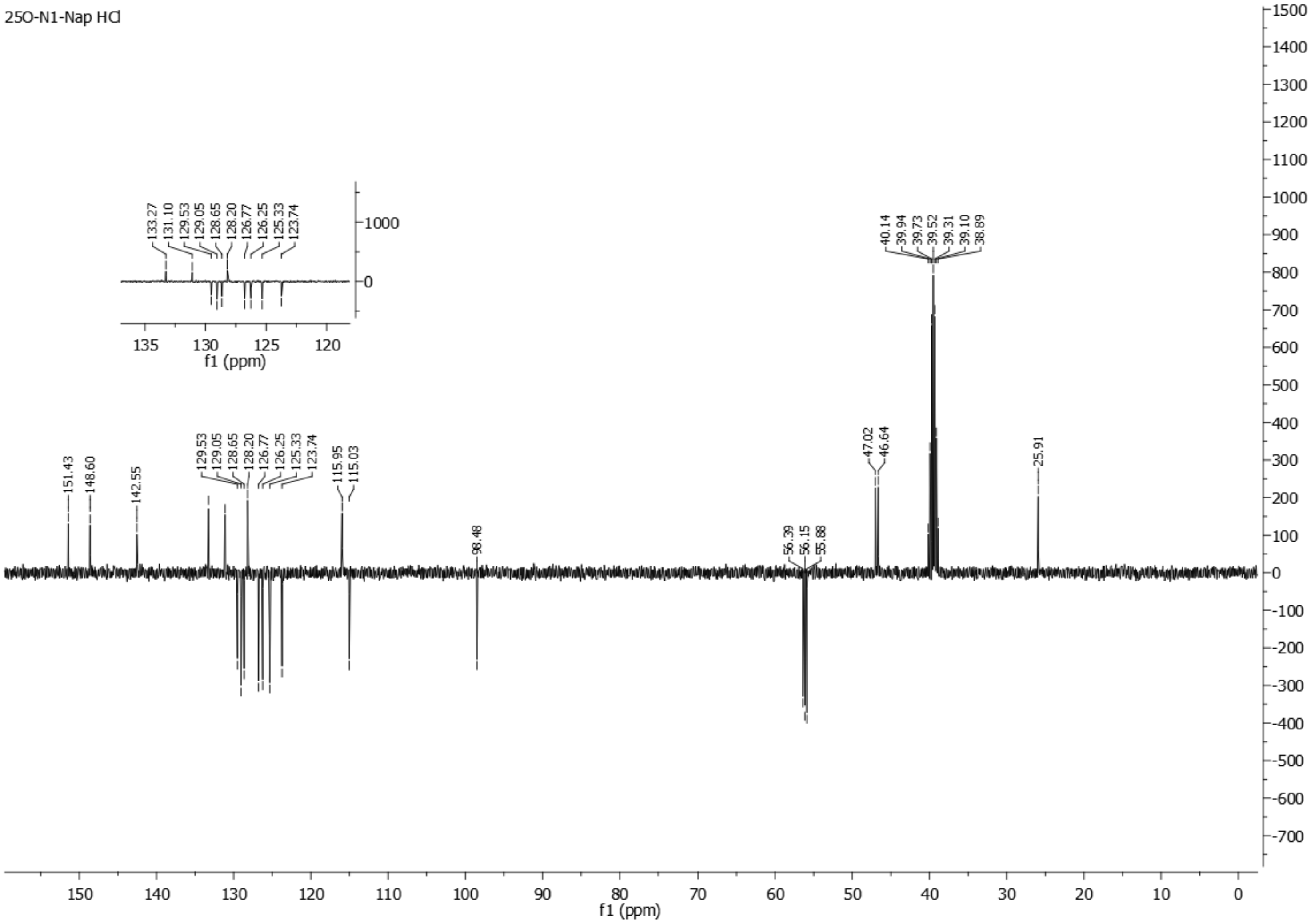

Spectra 62. ^{13}C NMR spectrum of **2C2-N1-Nap (29) HCl**

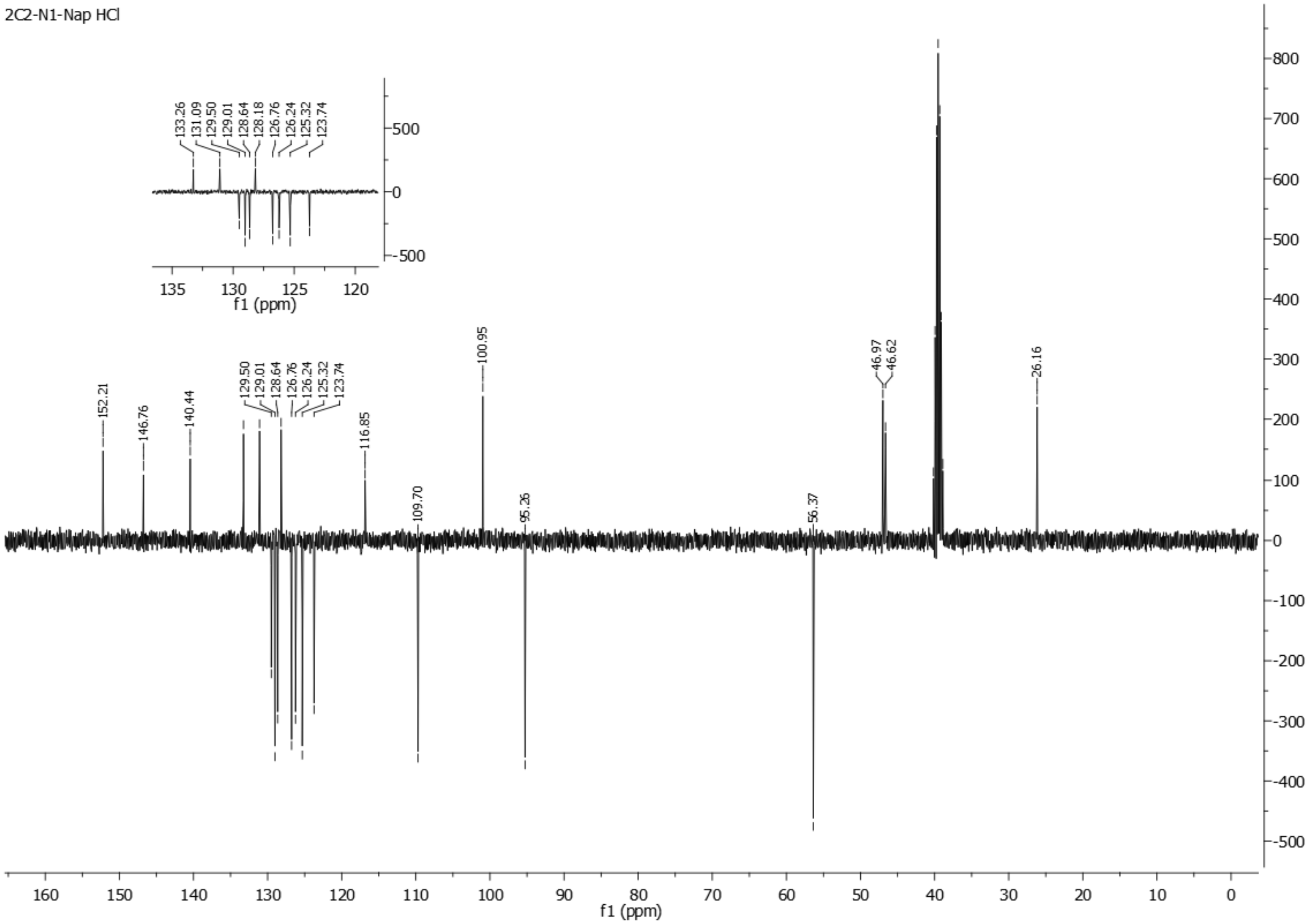

Spectra 63. ^{13}C NMR spectrum of Ψ-DOB-N1-Nap (30) HCl

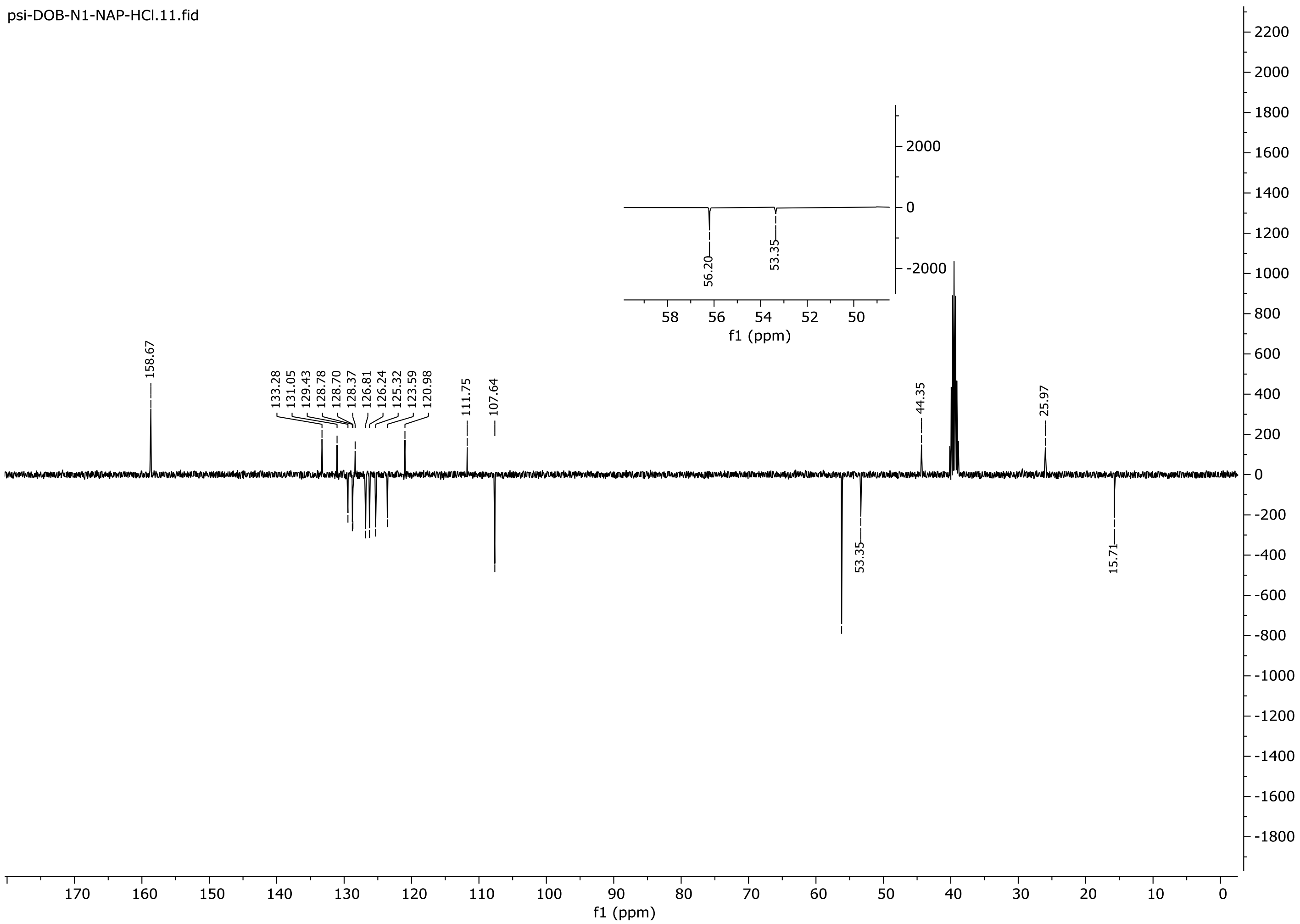

Spectra 64. ^{13}C NMR of 2C2-NBOMe (31) HCl

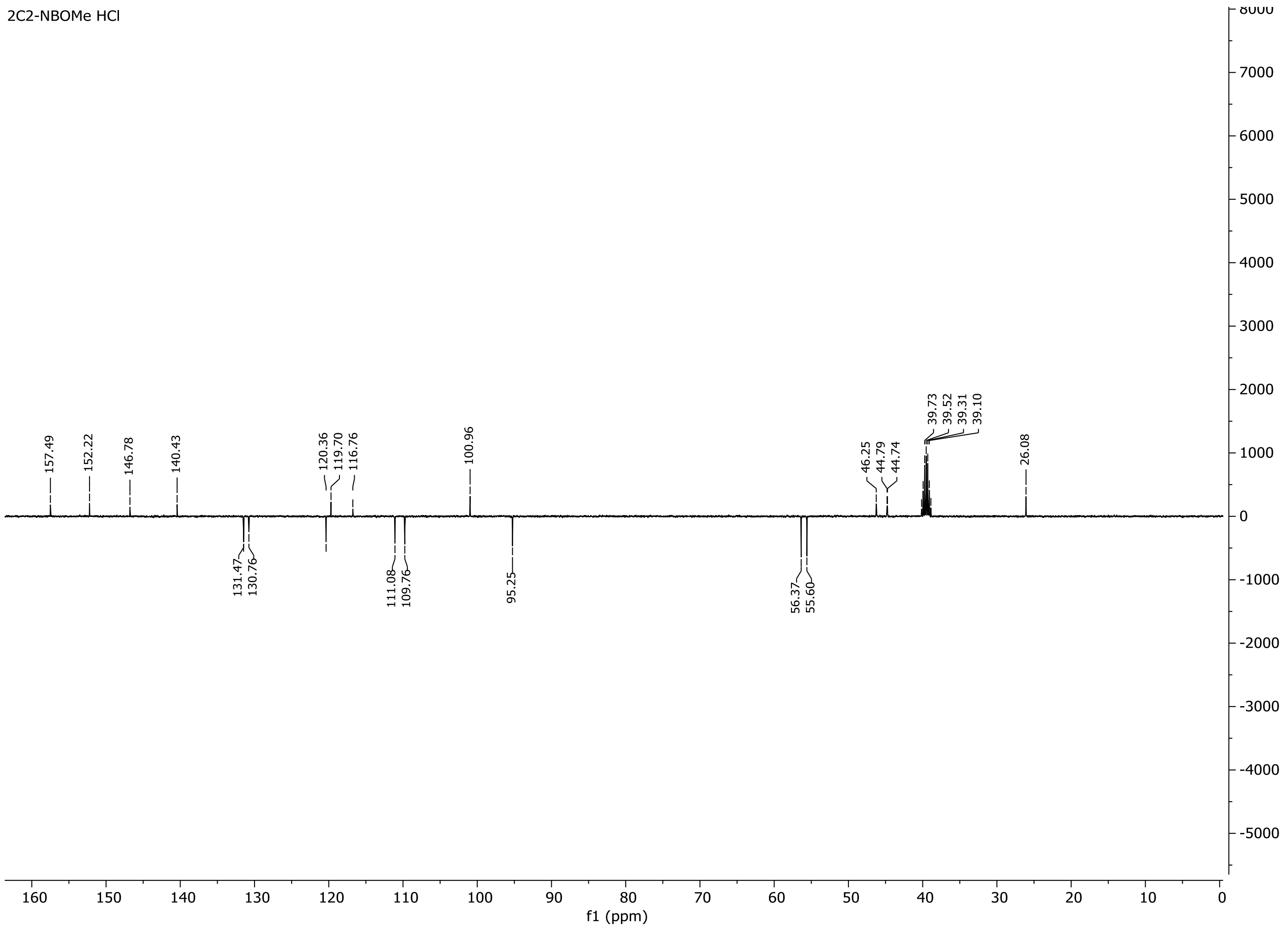

Spectra 65. ^{13}C NMR of 25O-NBOMe (32) HCl

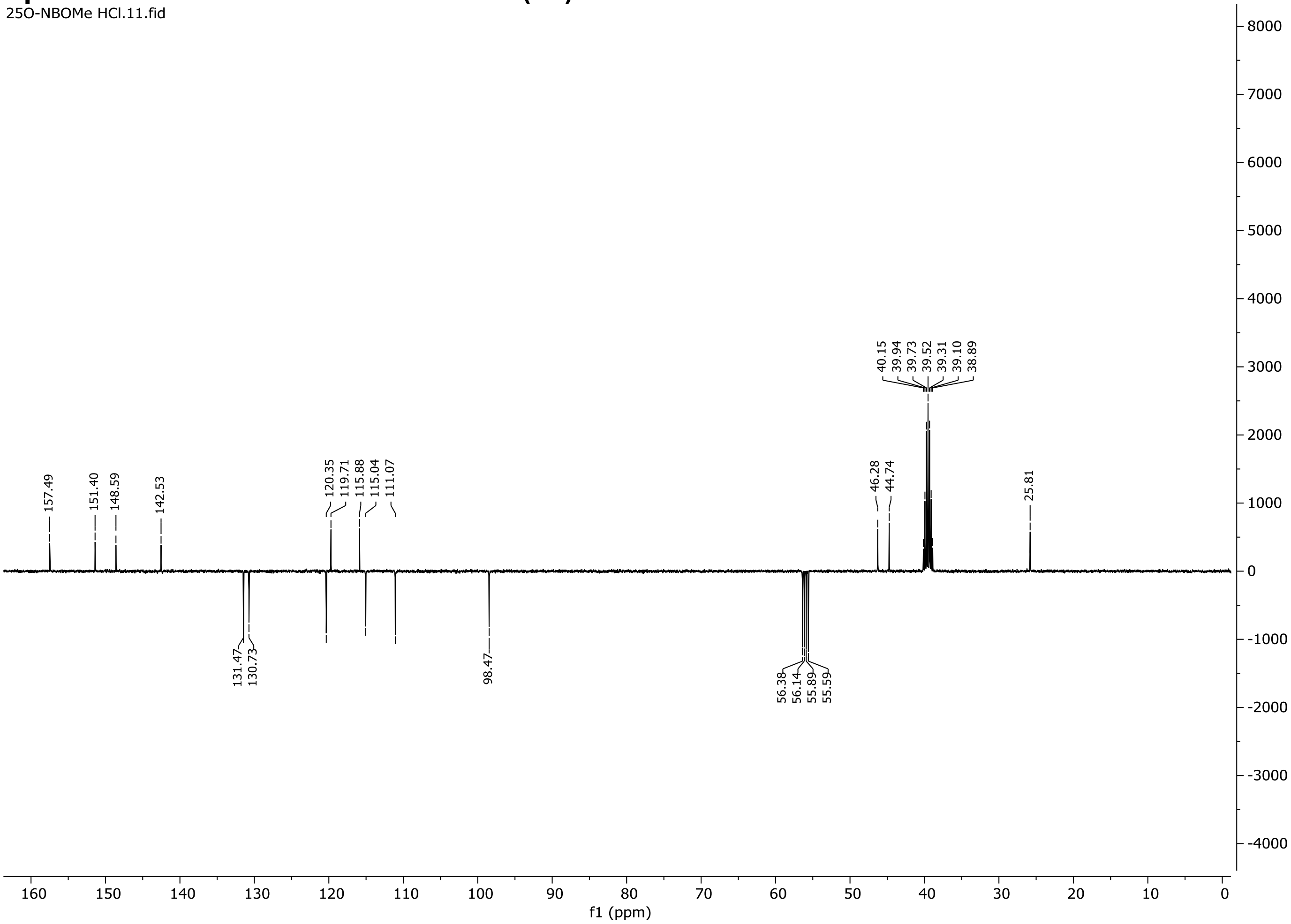

Spectra 66. ^{13}C NMR of 25O-NBcP (33) HCl

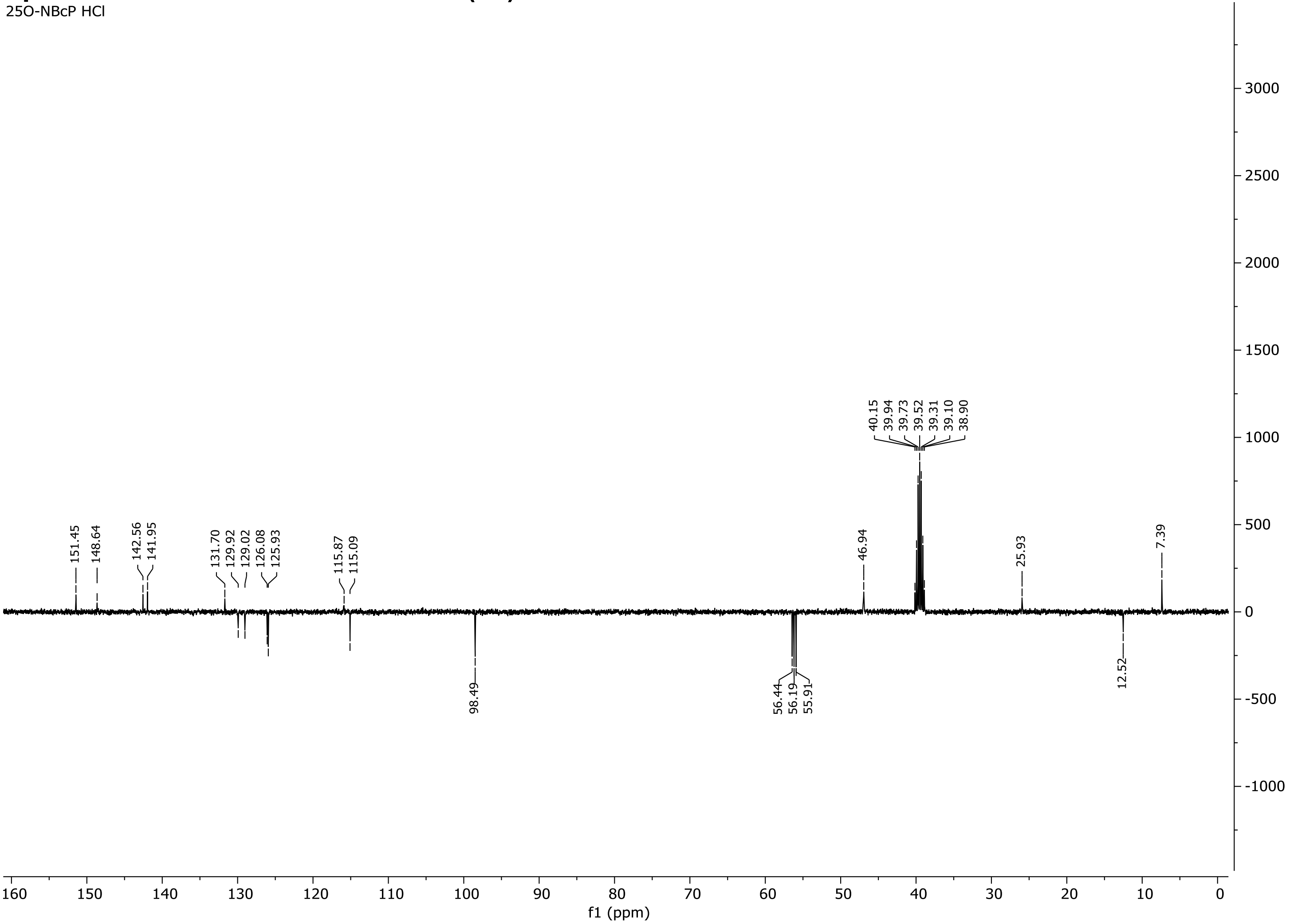

Spectra 67. ^{13}C NMR of 25O-NB-3-I (34) HCl

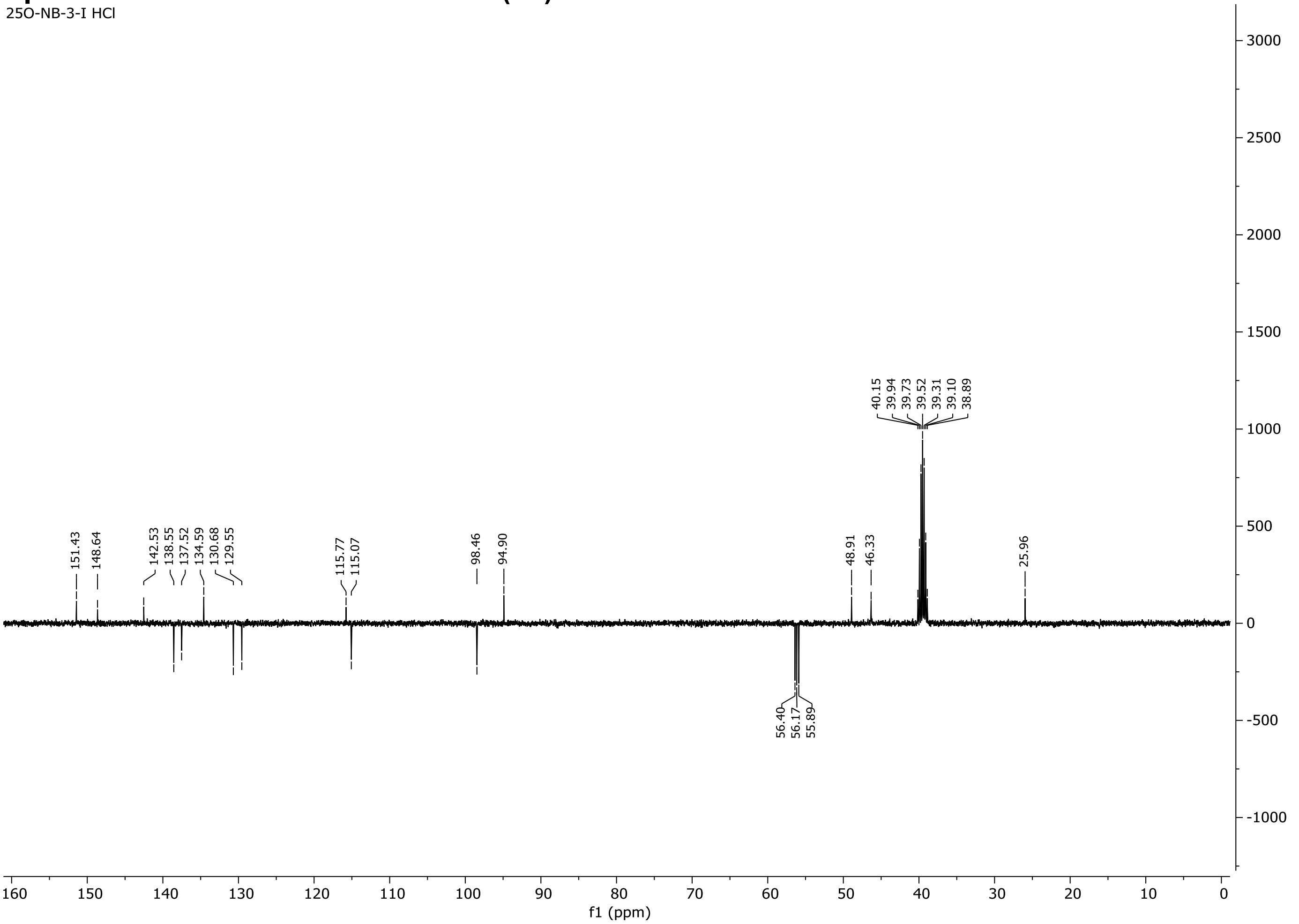

Spectra 68. ^{13}C NMR of 25O-NBPh-10'-OH (35) HCl

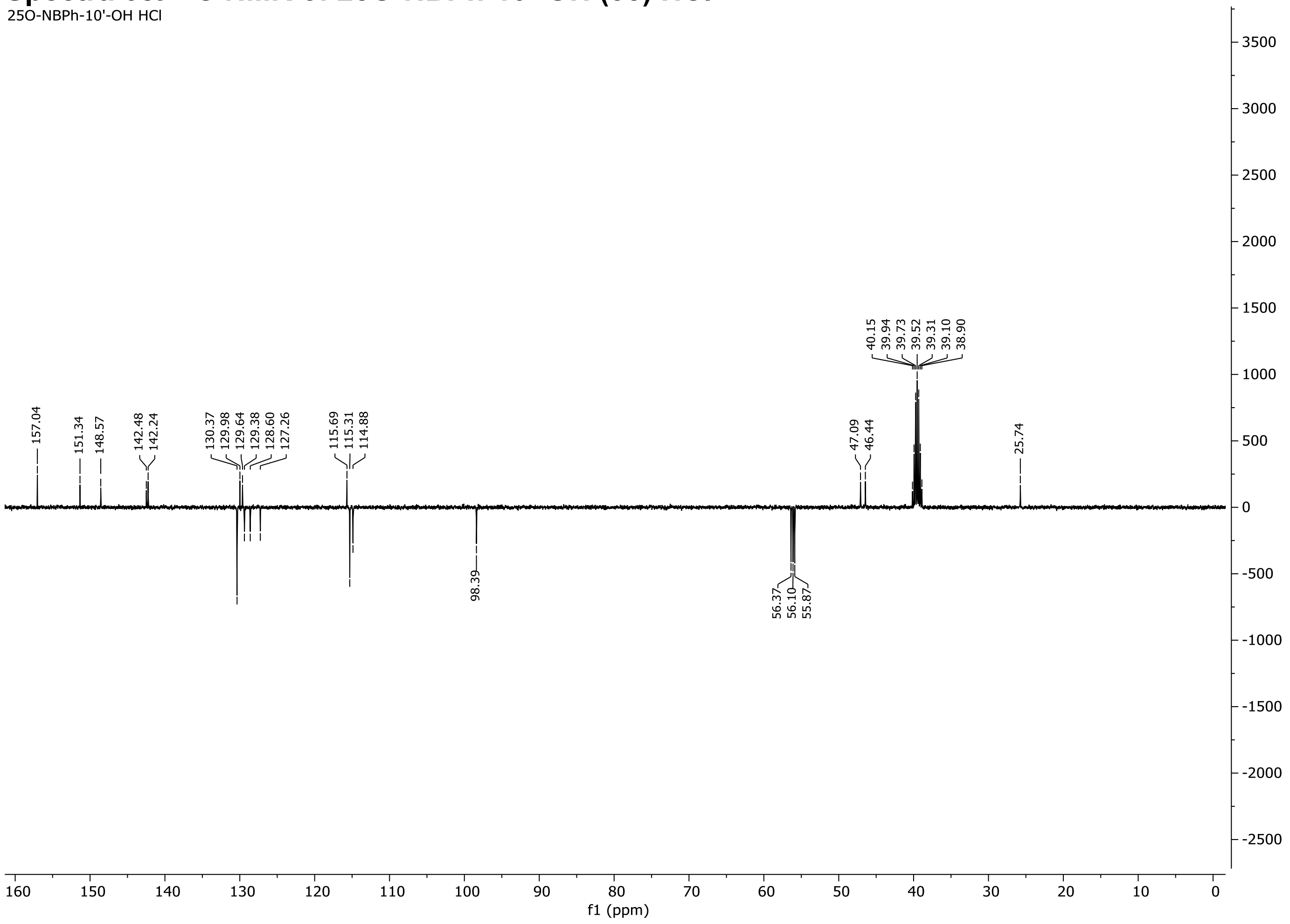

Spectra 69. 19F NMR of 25N-NBF (7) HCl

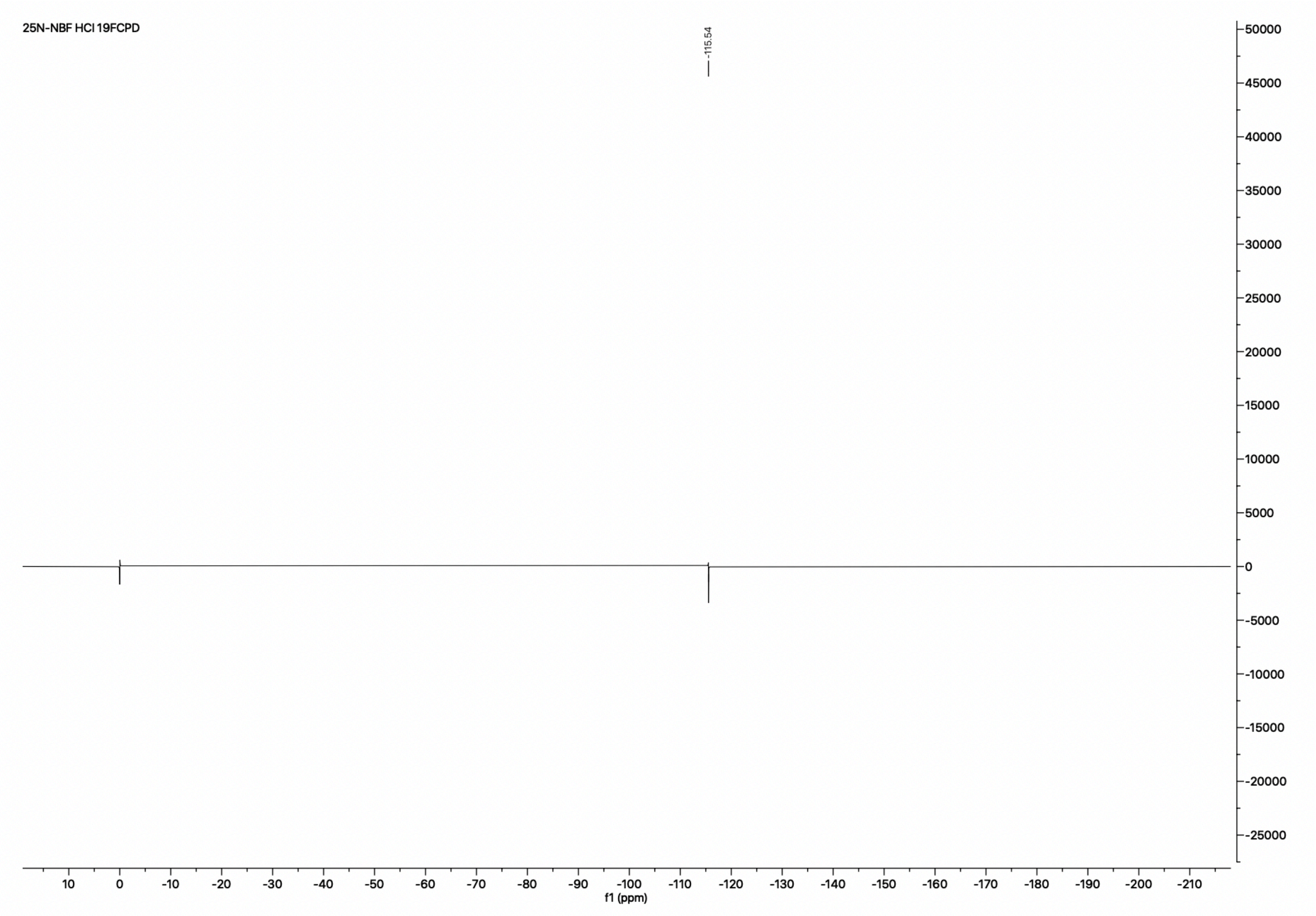

Spectra 70. ^{19}F NMR of 25N-NBOCF$_2$H (11) HCl

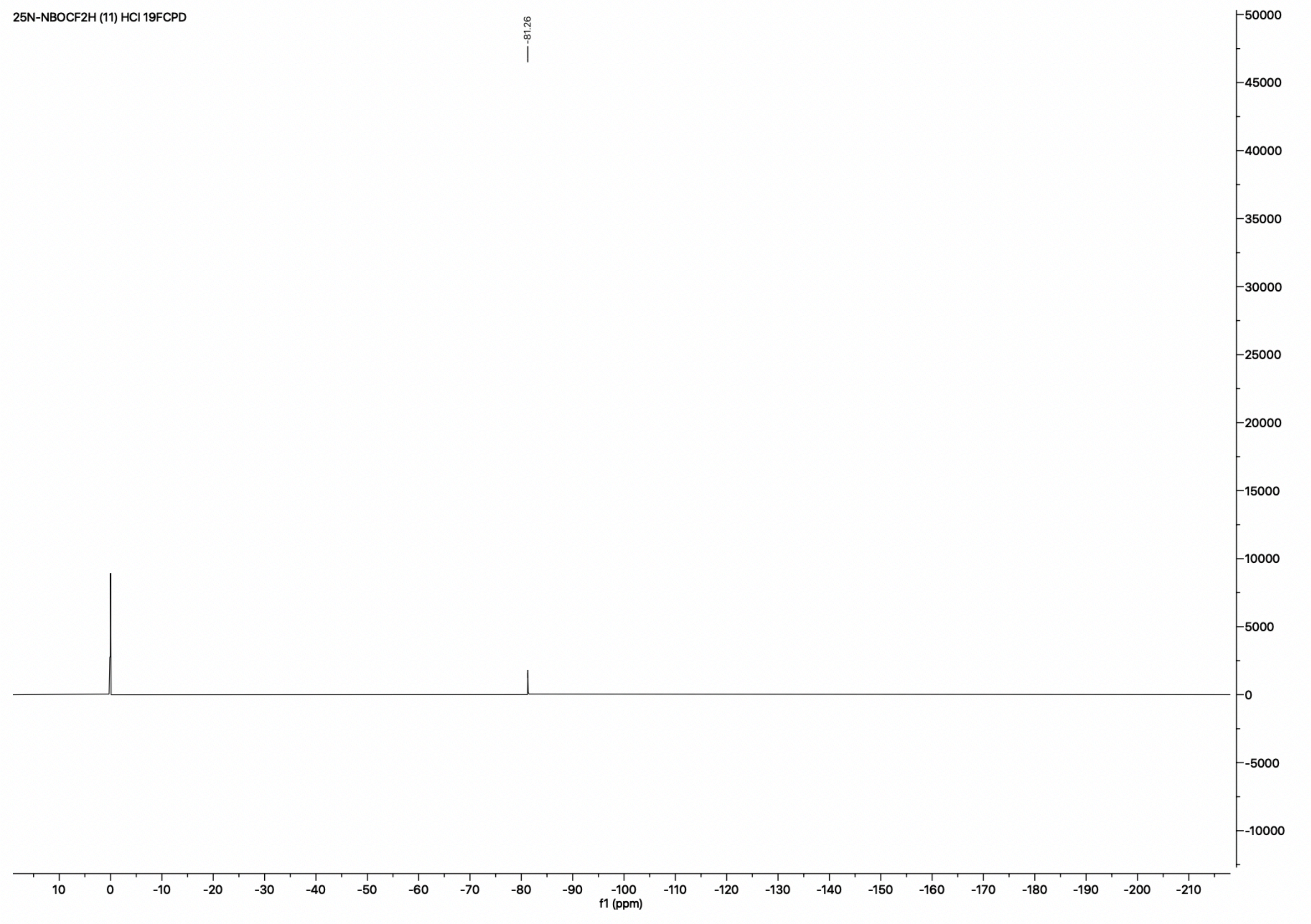

Spectra 71. ^{19}F NMR of 25N-NBOCF$_3$ (12) HCl

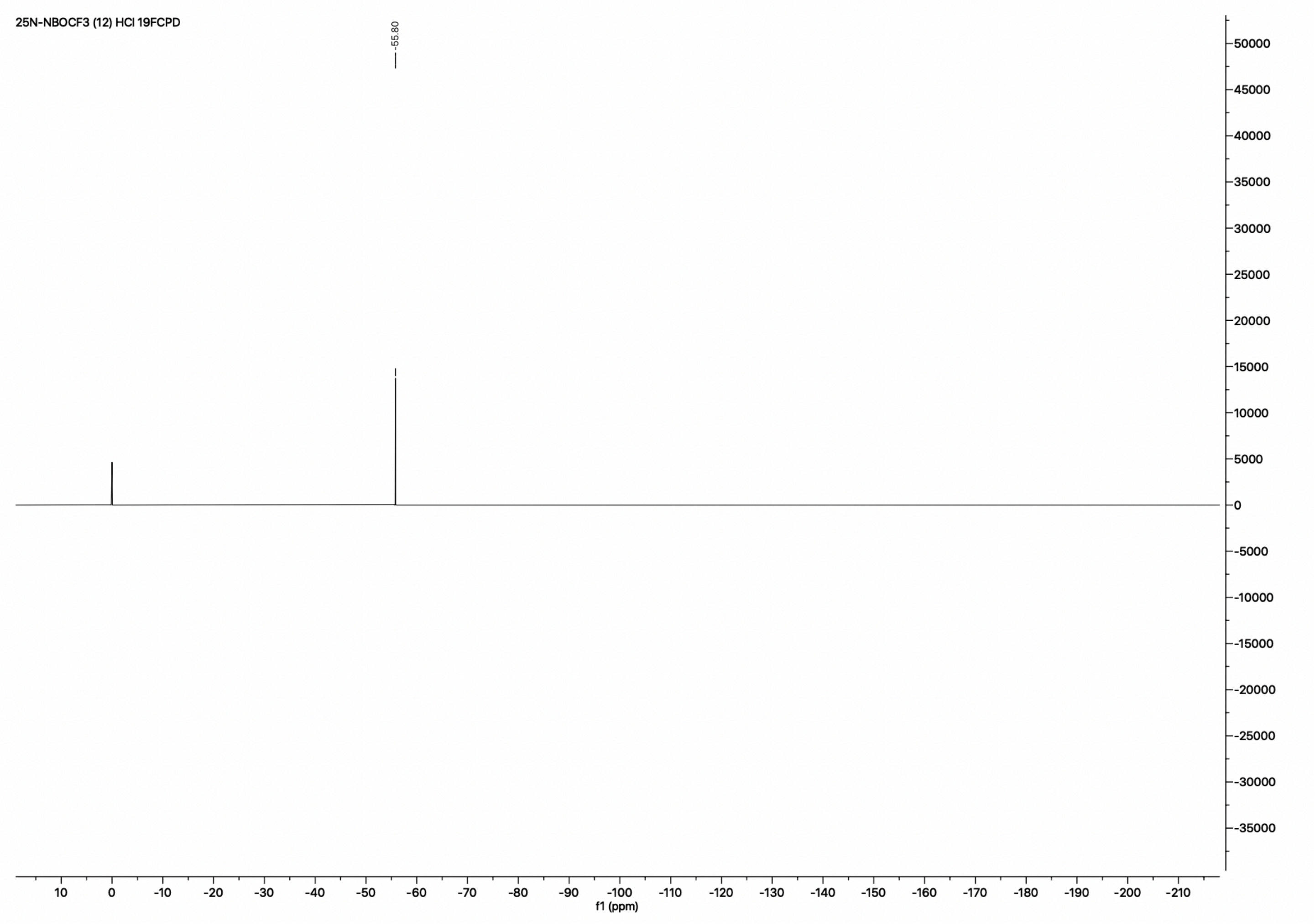

Spectra 72. ^{19}F NMR of 25N-NBMDF$_2$ (13) HCl

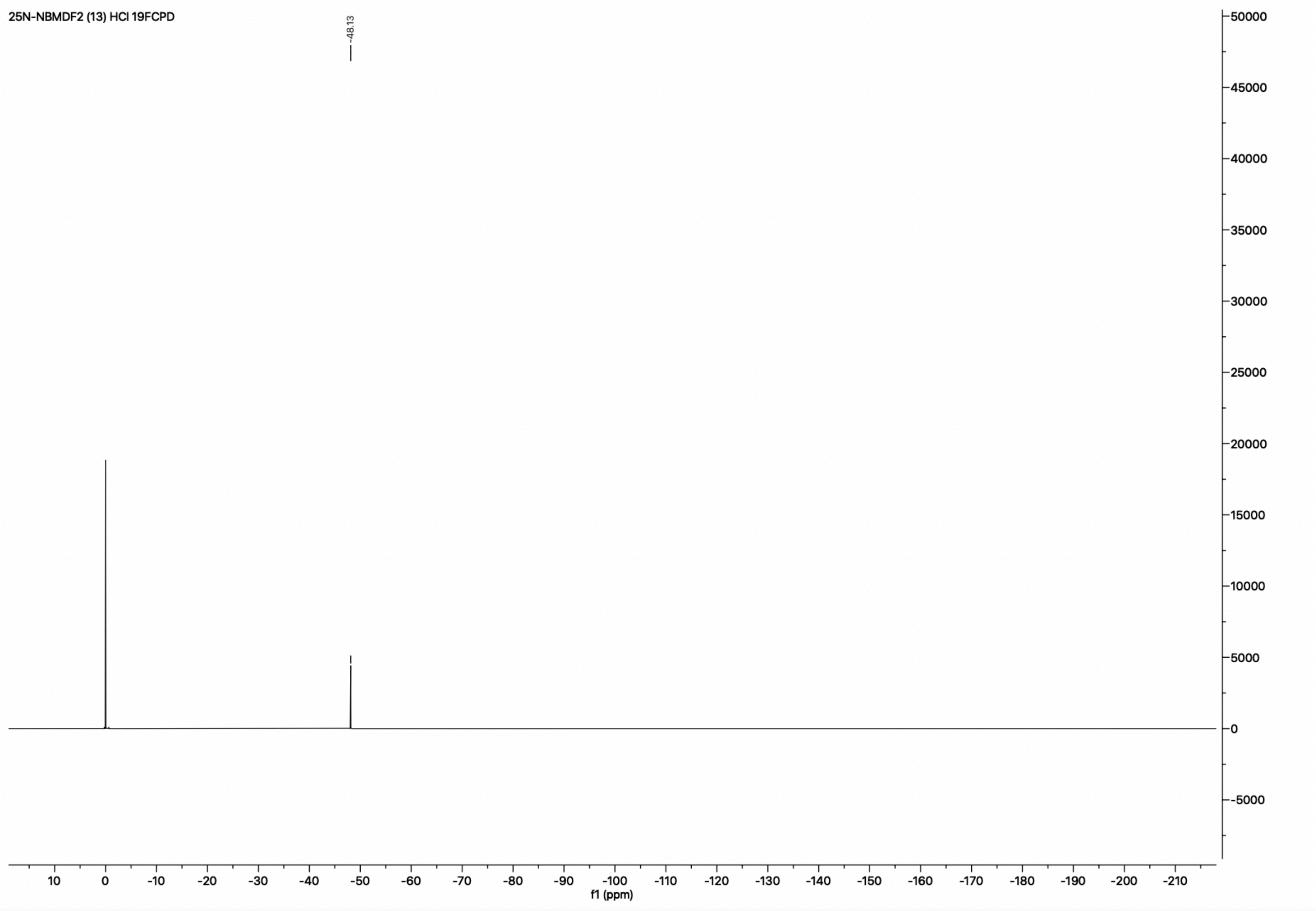

Spectra 73. ^{19}F NMR of 25N-NBCF$_3$ (14) HCl

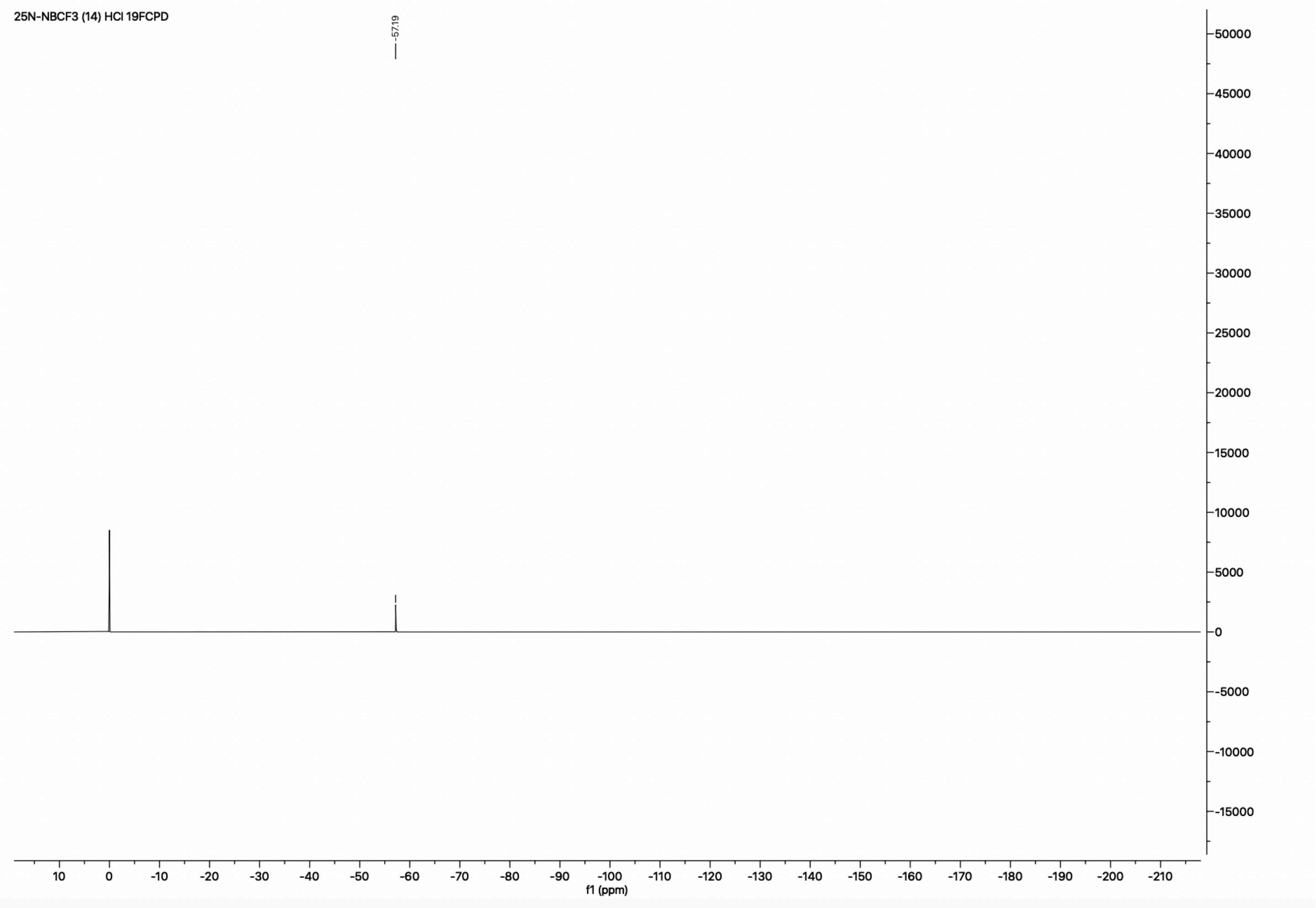

Spectra 74. ^{19}F NMR of 25N-NB-2-MeO-3-F (19) HCl

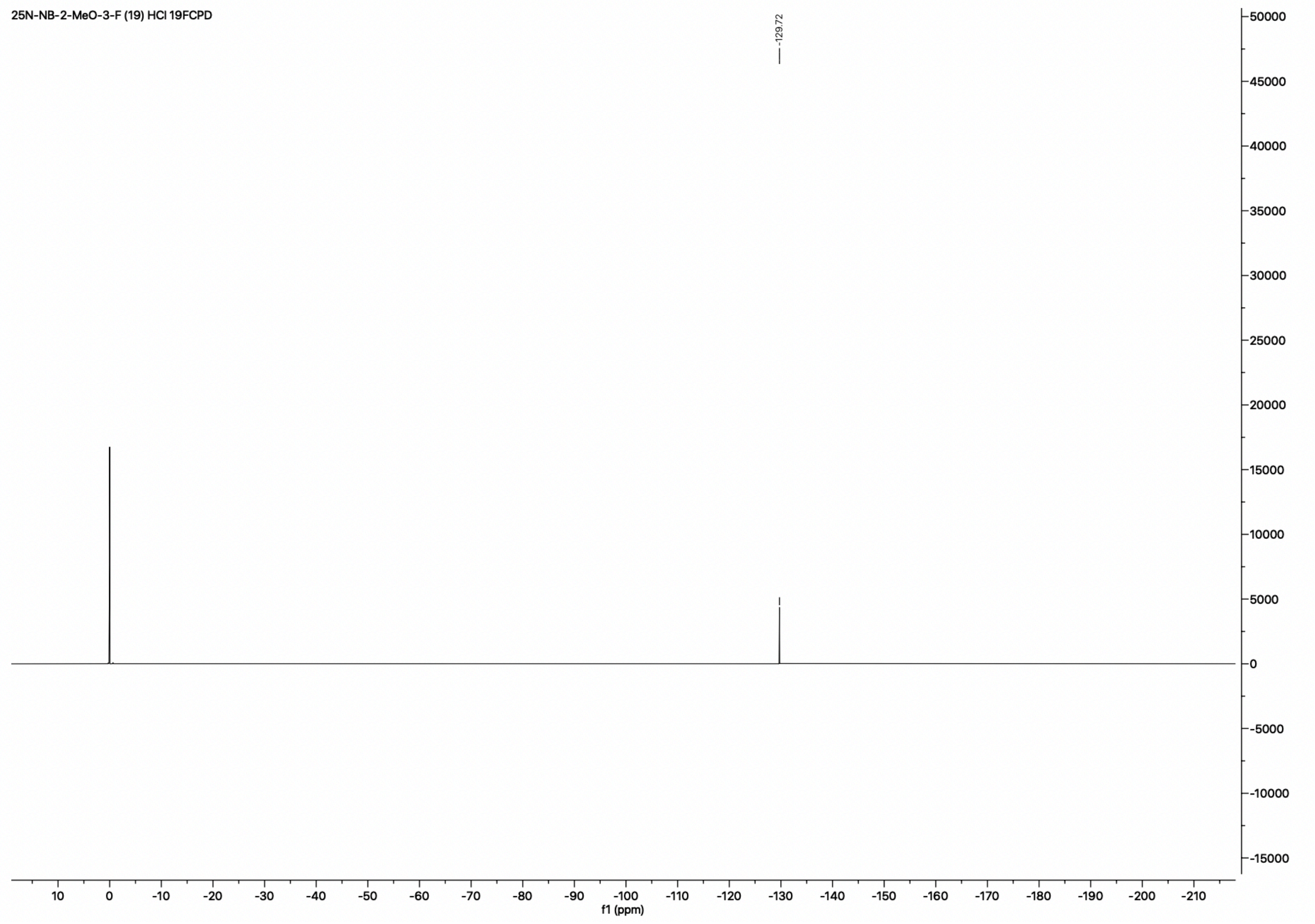

Spectra 75. ^{19}F NMR of 25N-NB-3-F (24) HCl

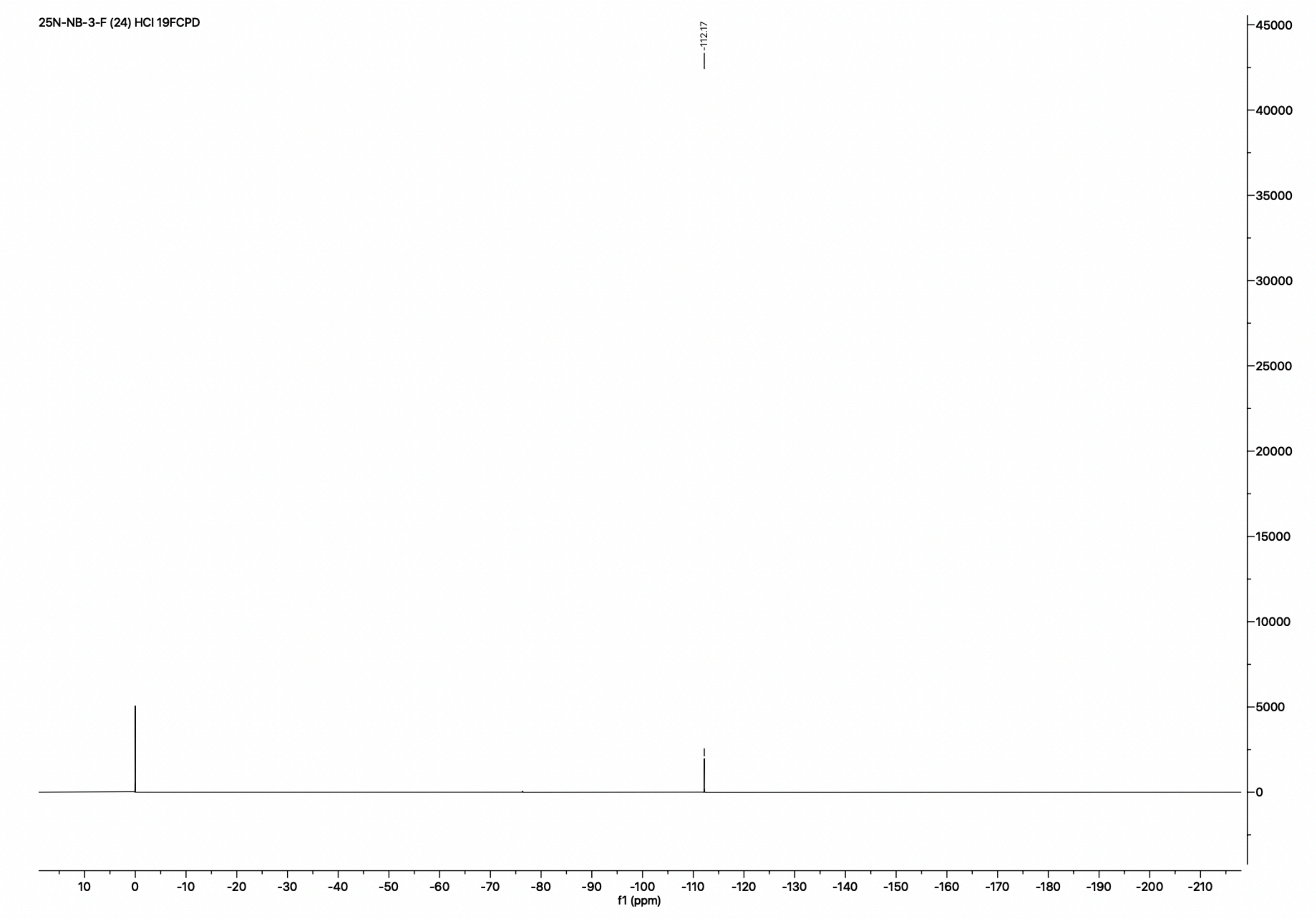

Spectra 76. ^{19}F NMR of 25N-NB-4-F (25) HCl

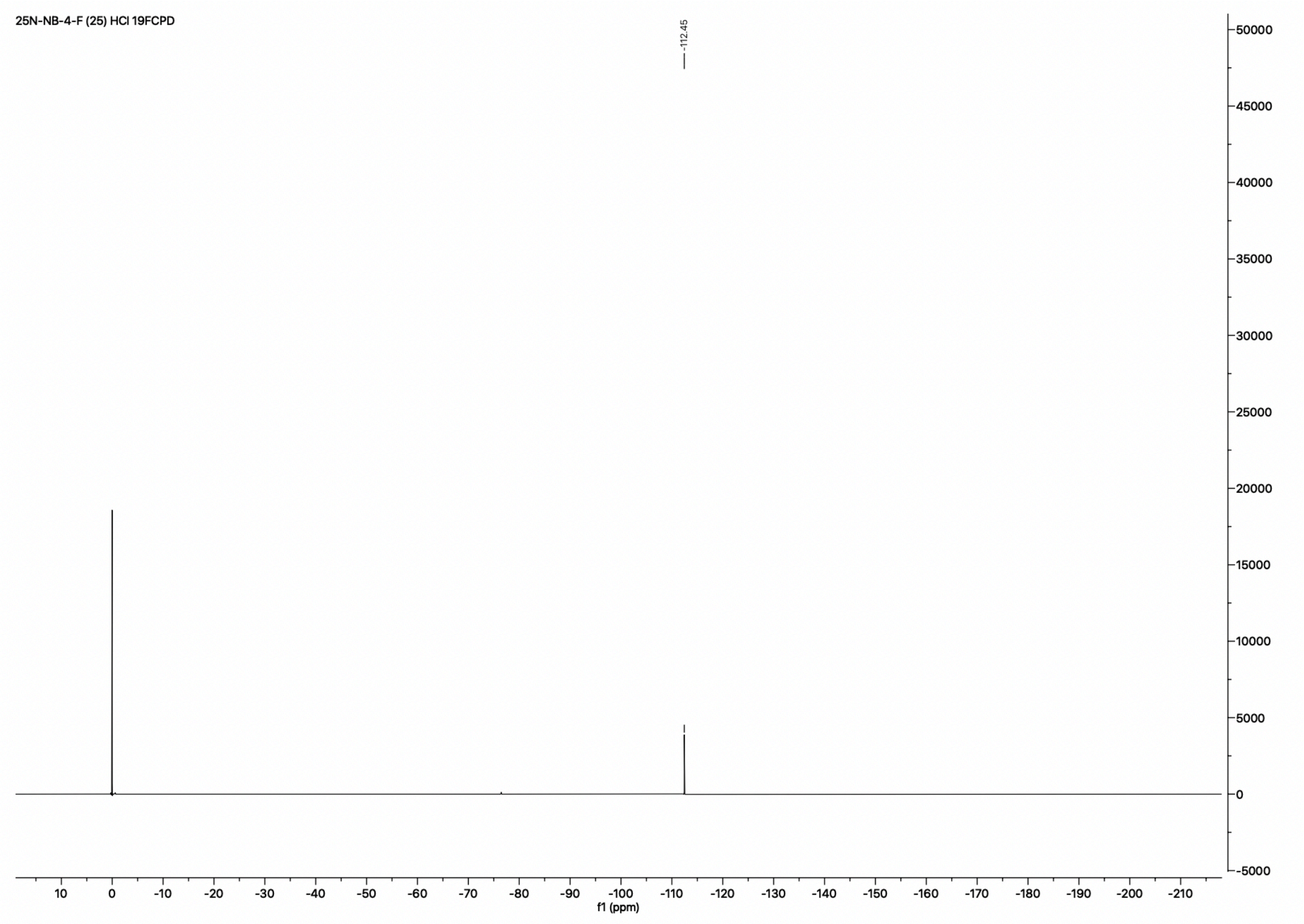

Supplement: Supplementary file 5 — Supplementary Dataset 2 [file 41467_2023_44016_MOESM5_ESM.pdf]
