# Supplementary material for: Identification of 5-HT2A receptor signaling pathways associated with psychedelic potential
Source: Nat Commun. 2023 Dec 15;14:8221. doi: 10.1038/s41467-023-44016-1 (PMC10724237; doi:10.1038/s41467-023-44016-1)

HPLC Traces of Final *N*-Benzyl Phenethylamines. HPLC experiments performed on HCl salts at 1 mg/mL.

HPLC Trace of 25N-NB (2). Purity (Peak area): 99.5%; Wavelength: 220 nm.

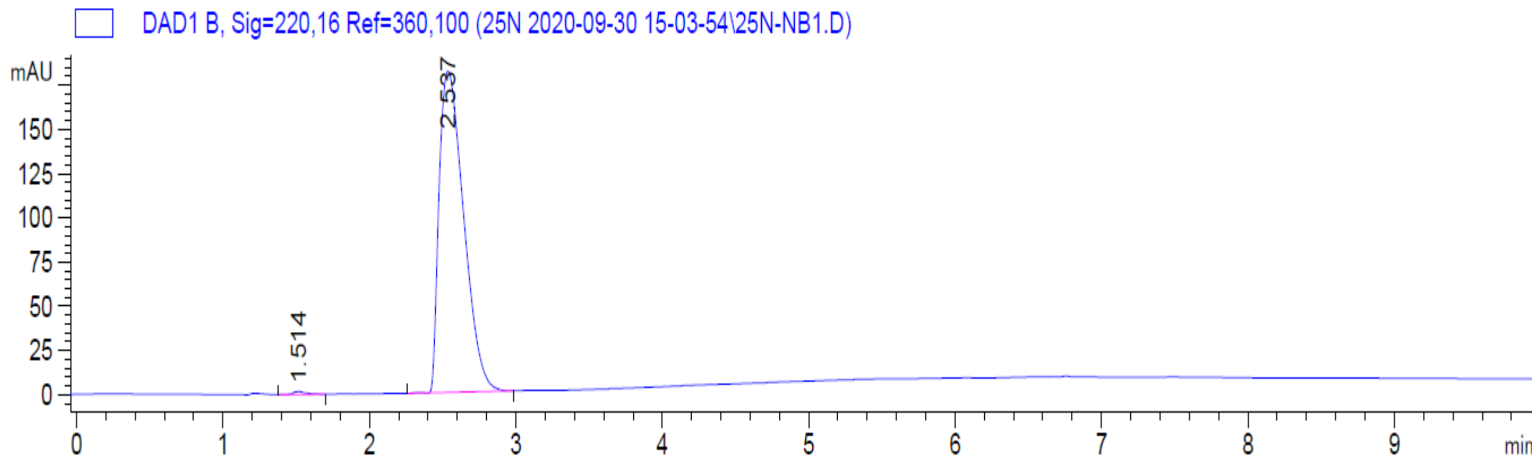

HPLC Trace of 25N-NBOH (3). Purity (Peak area): 97.4%; Wavelength: 220 nm.

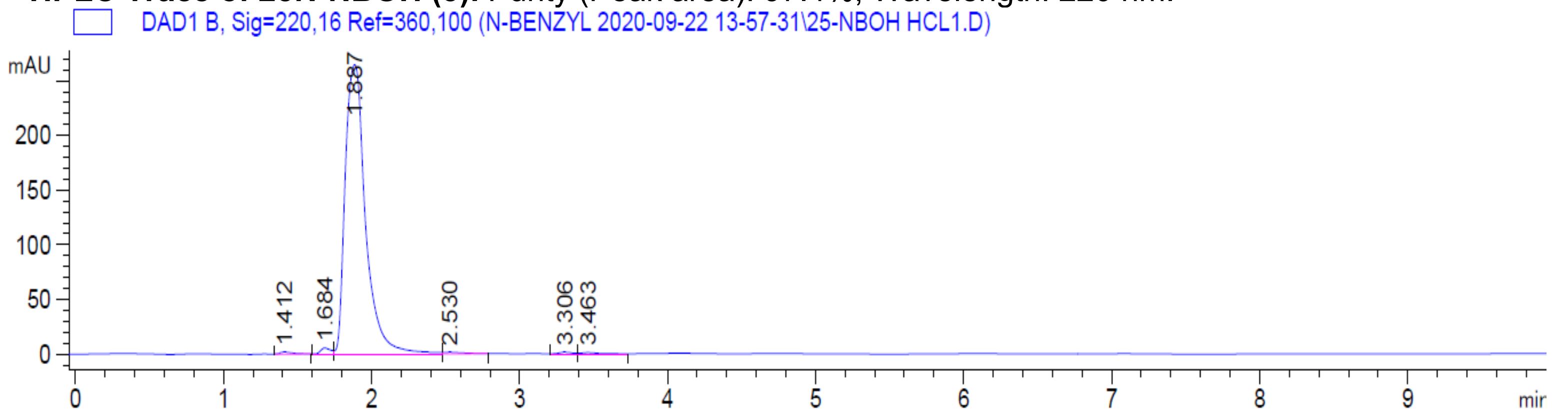

HPLC Trace of 25N-NBOMe (4). Purity (Peak area): 98.7%; Wavelength: 220 nm.

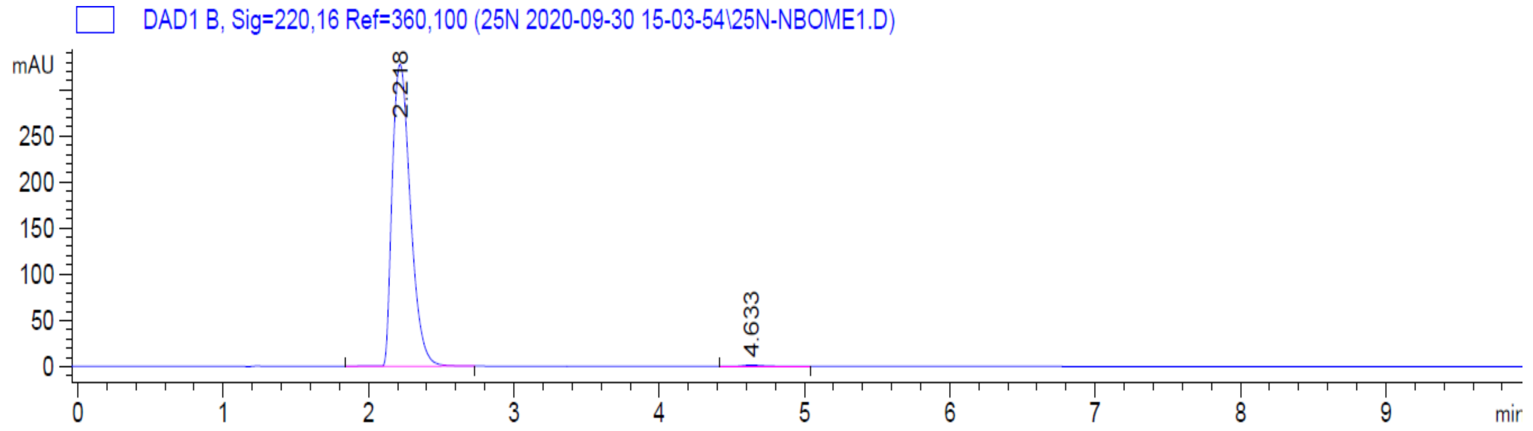

HPLC Trace of 25N-NBOEt (5). Purity (Peak area): 98.8%; Wavelength: 220 nm.

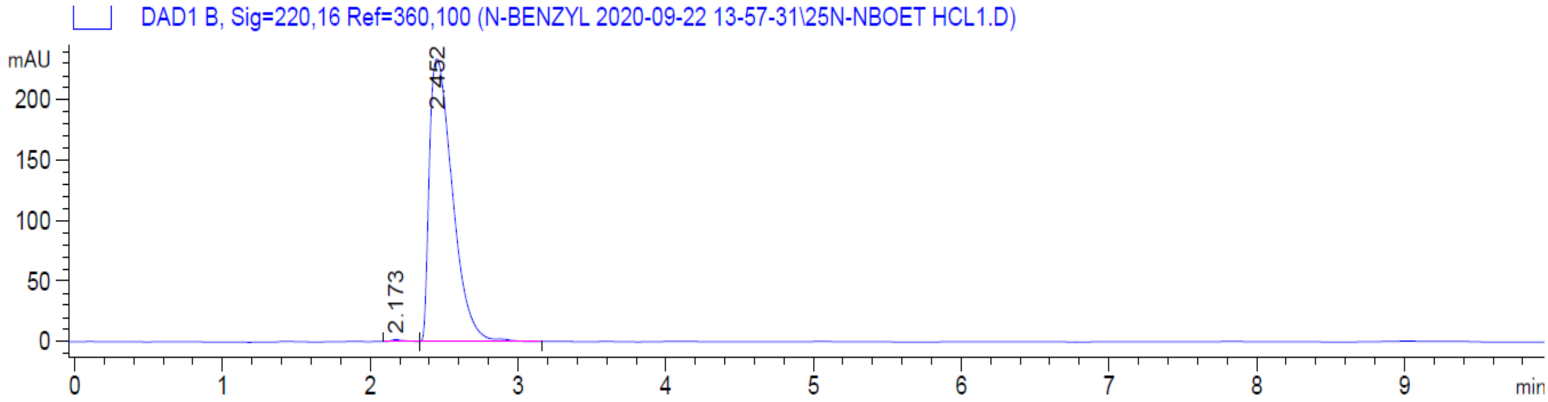

HPLC Trace of 25N-NBMe (6). Purity (Peak area): 98.4%; Wavelength: 220 nm.

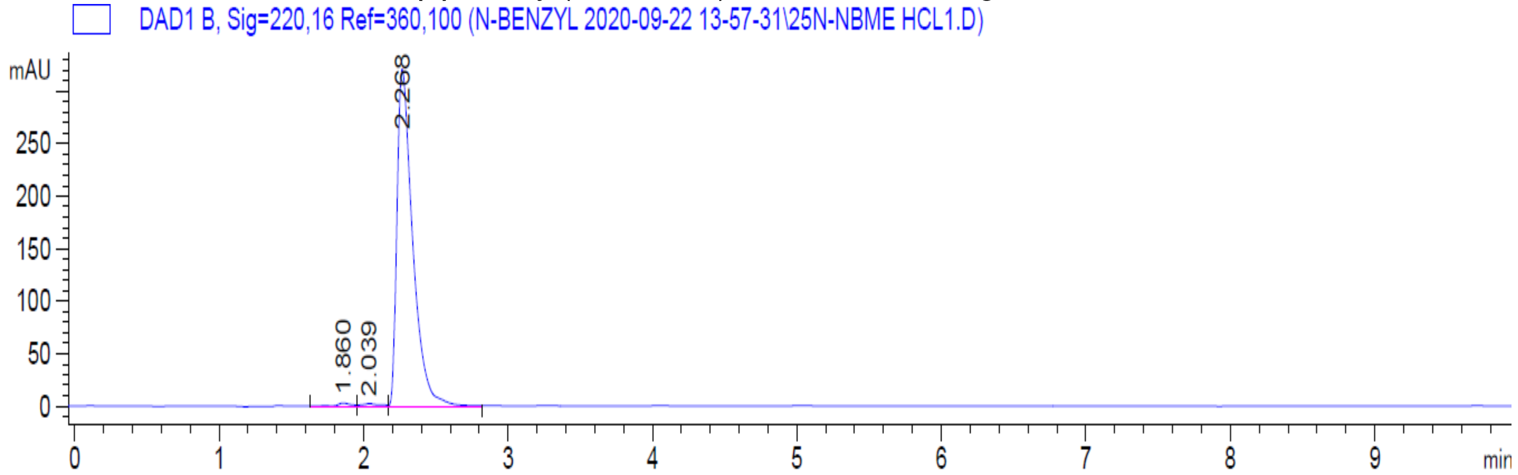

HPLC Trace of 25N-NBF (7). Purity (Peak area): 100%; Wavelength: 220 nm.

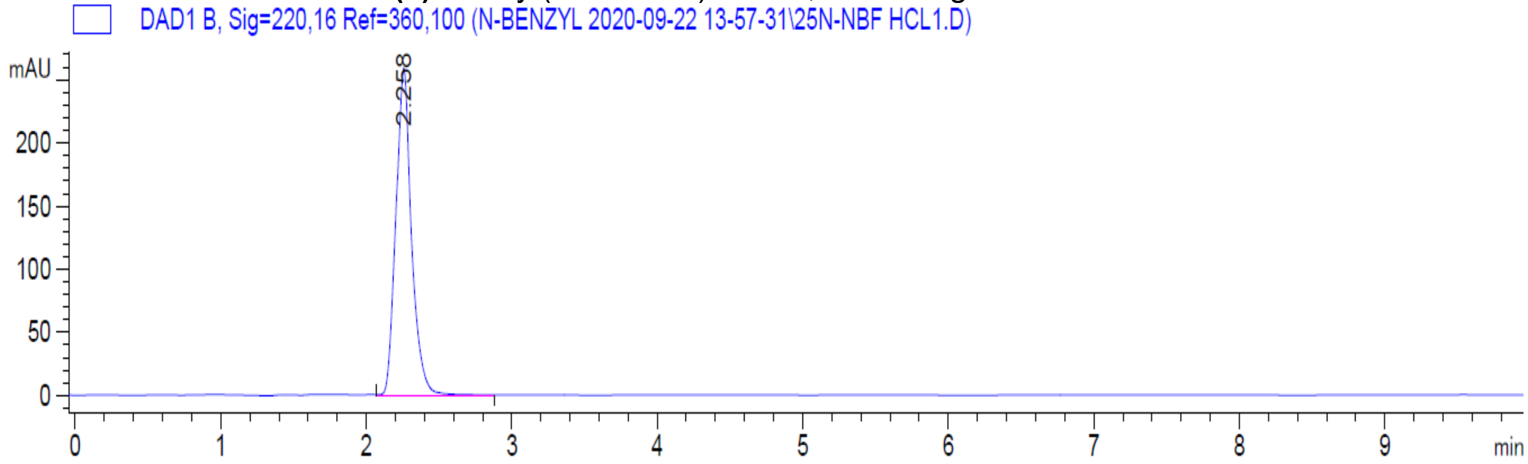

HPLC Trace of 25N-NBCl (8). Purity (Peak area): 98.8%; Wavelength: 220 nm.

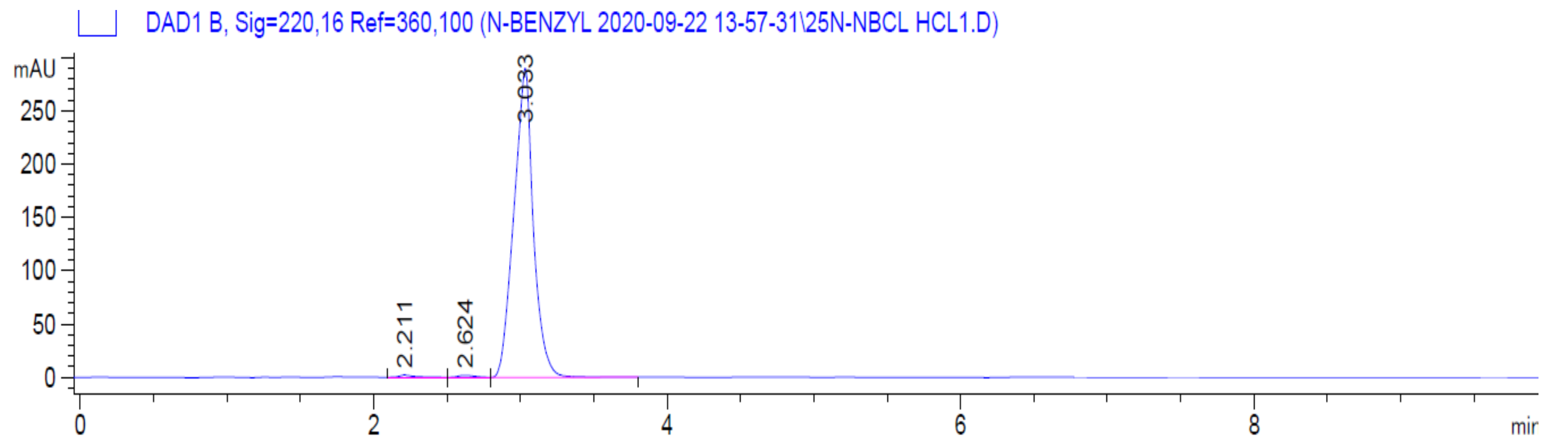

HPLC Trace of 25N-NBBr (9). Purity (Peak area): 99.2%; Wavelength: 220 nm.

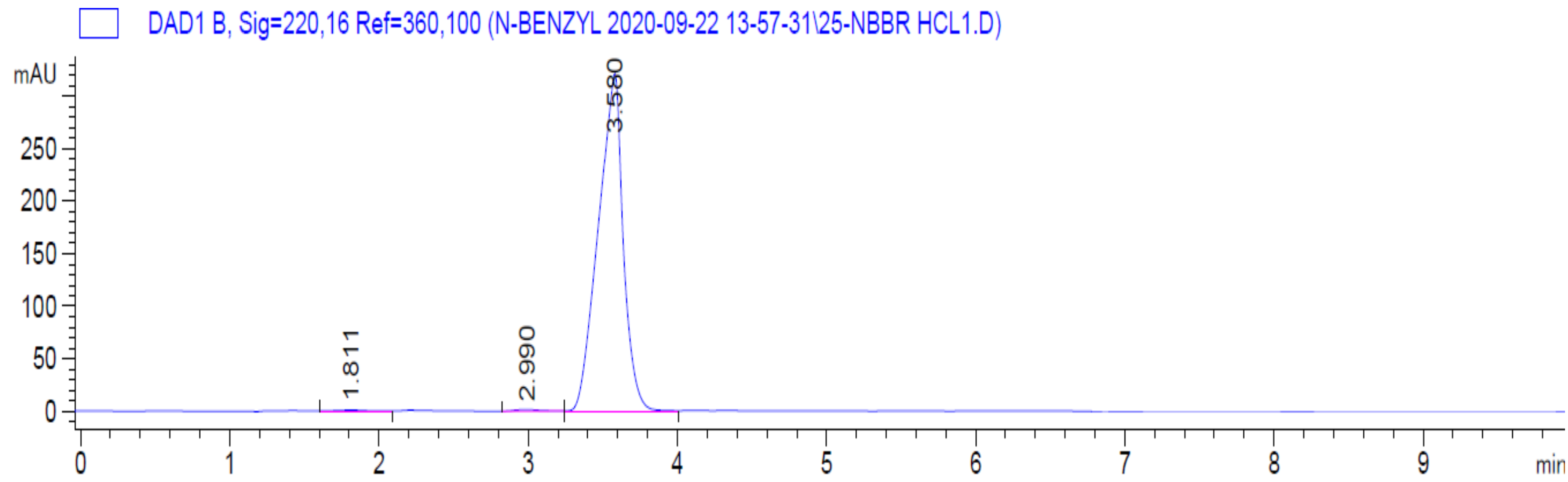

HPLC Trace of 25N-NBI (10). Purity (Peak area): 99.0%; Wavelength: 220 nm.

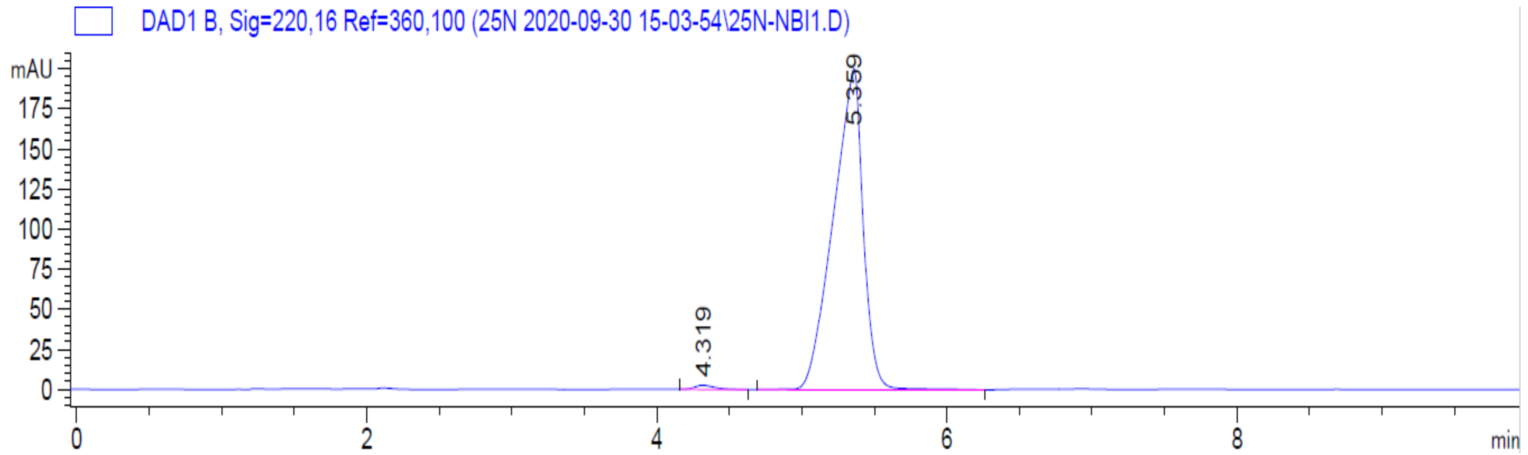

HPLC Trace of 25N-NBOCF$_2$H (11). Purity (Peak area): 93.1%; Wavelength: 220 nm.

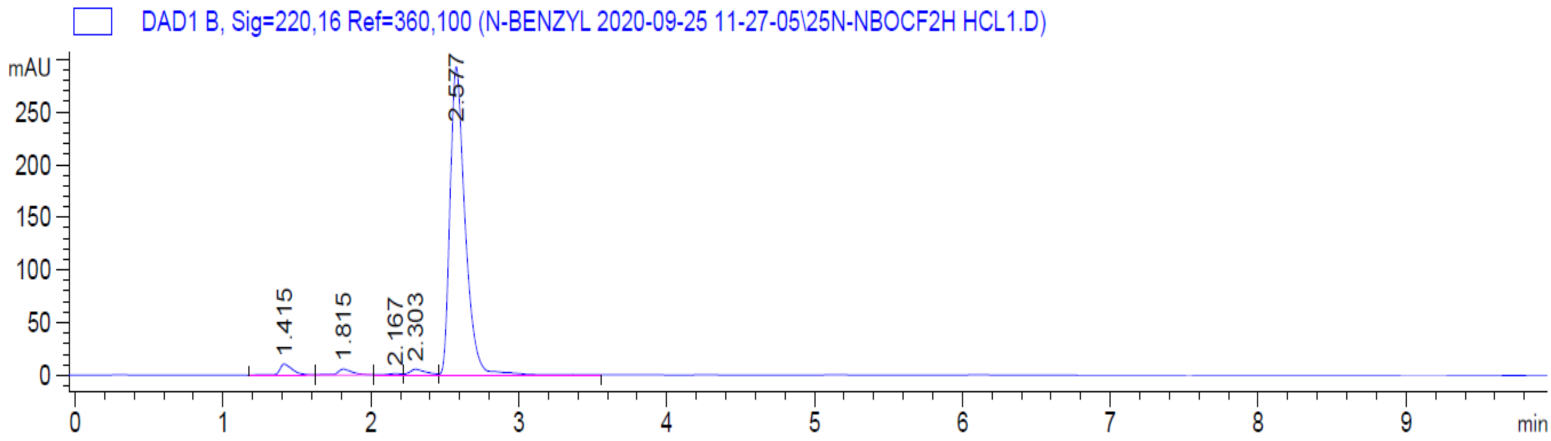

HPLC Trace of 25N-NBOCF$_3$ (12). Purity (Peak area): 98.7%; Wavelength: 220 nm.

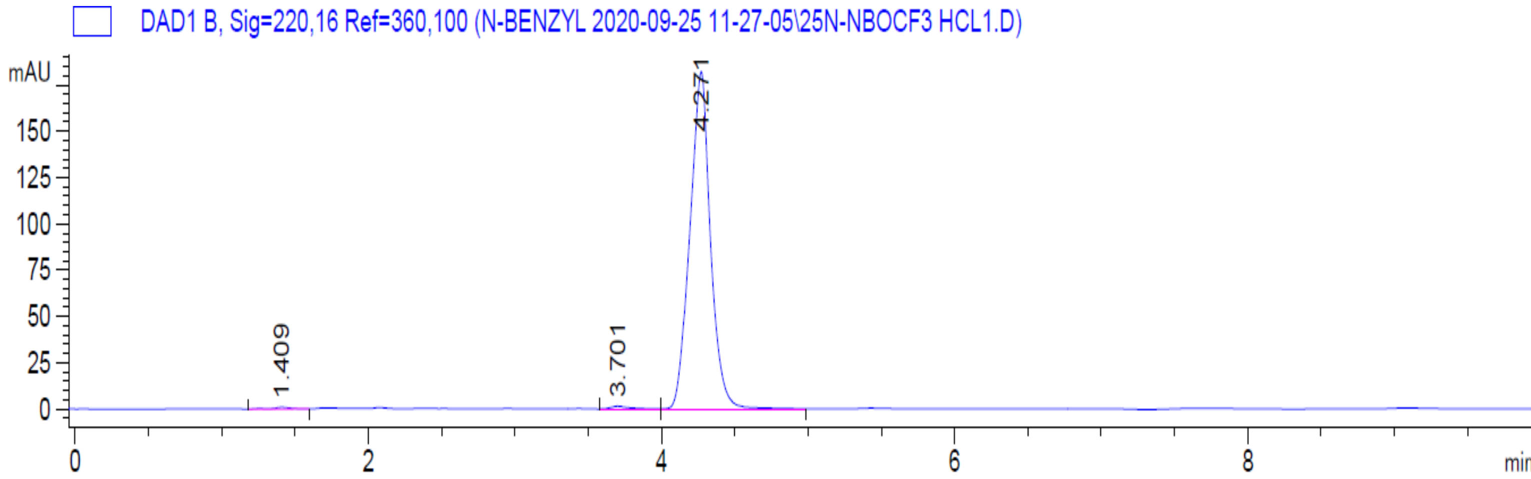

HPLC Trace of 25N-NBMDF$_2$ (13). Purity (Peak area): 98.9%; Wavelength: 220 nm.

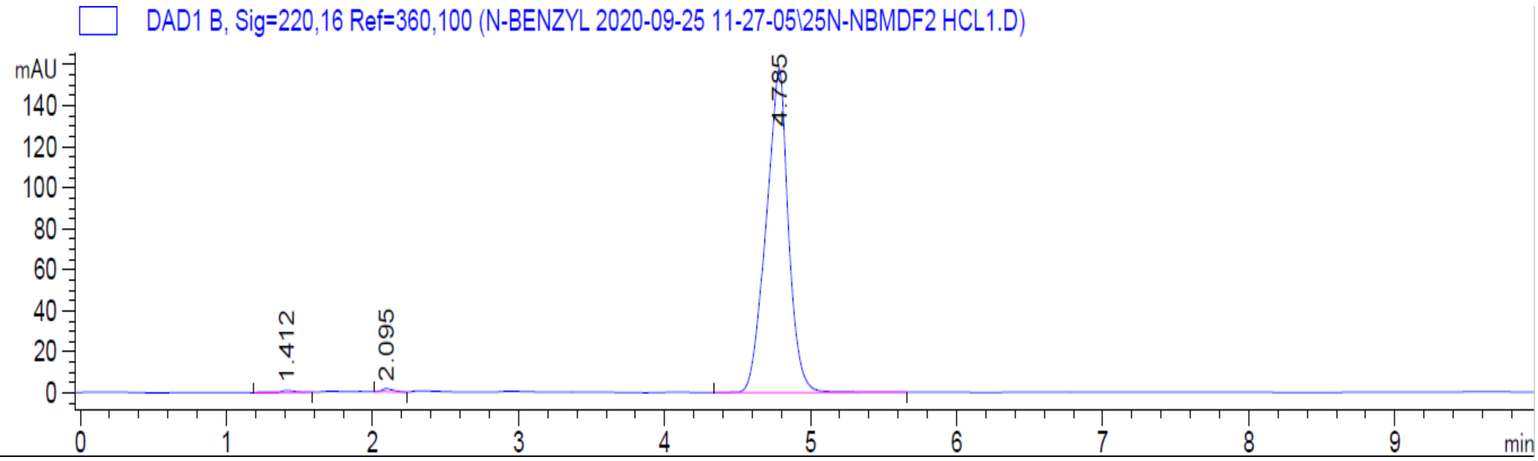

HPLC Trace of 25N-NBCF$_3$ (14). Purity (Peak area): 99.2%; Wavelength: 220 nm.

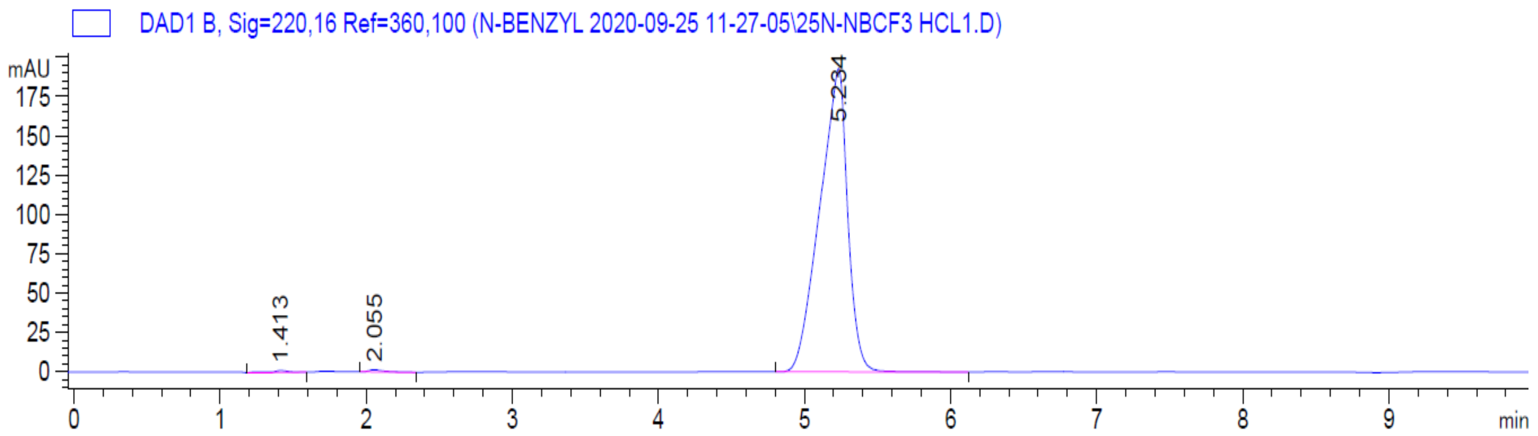

HPLC Trace of 25N-NBNO$_2$ (15). Purity (Peak area): 99.4%; Wavelength: 220 nm.

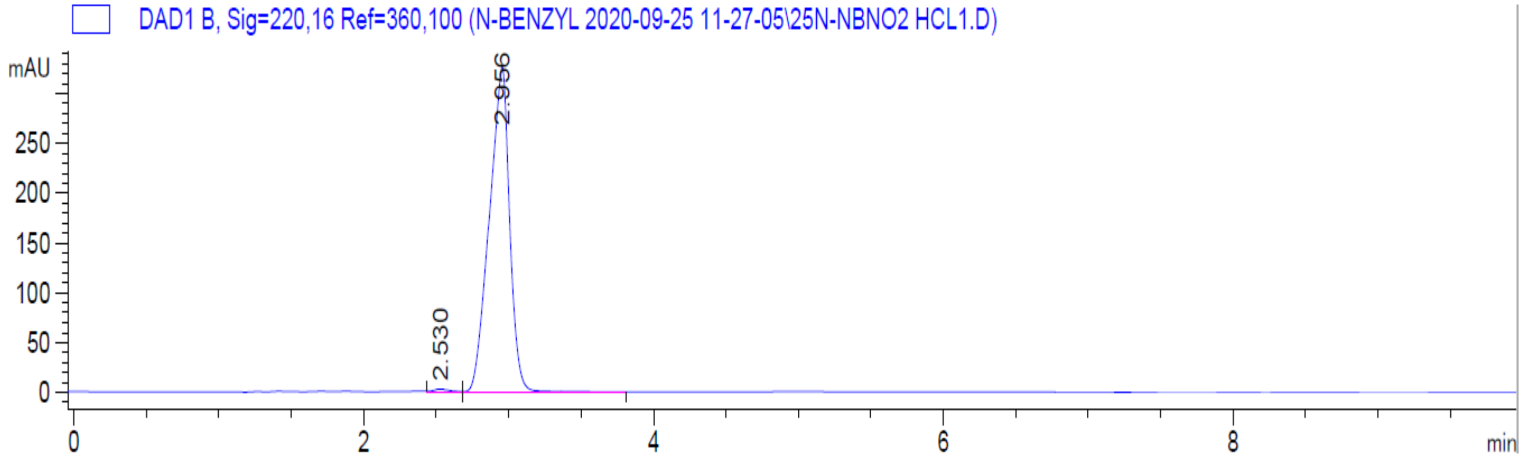

HPLC Trace of 25N-N1-Nap (16). Purity (Peak area): 99.2%; Wavelength: 220 nm.

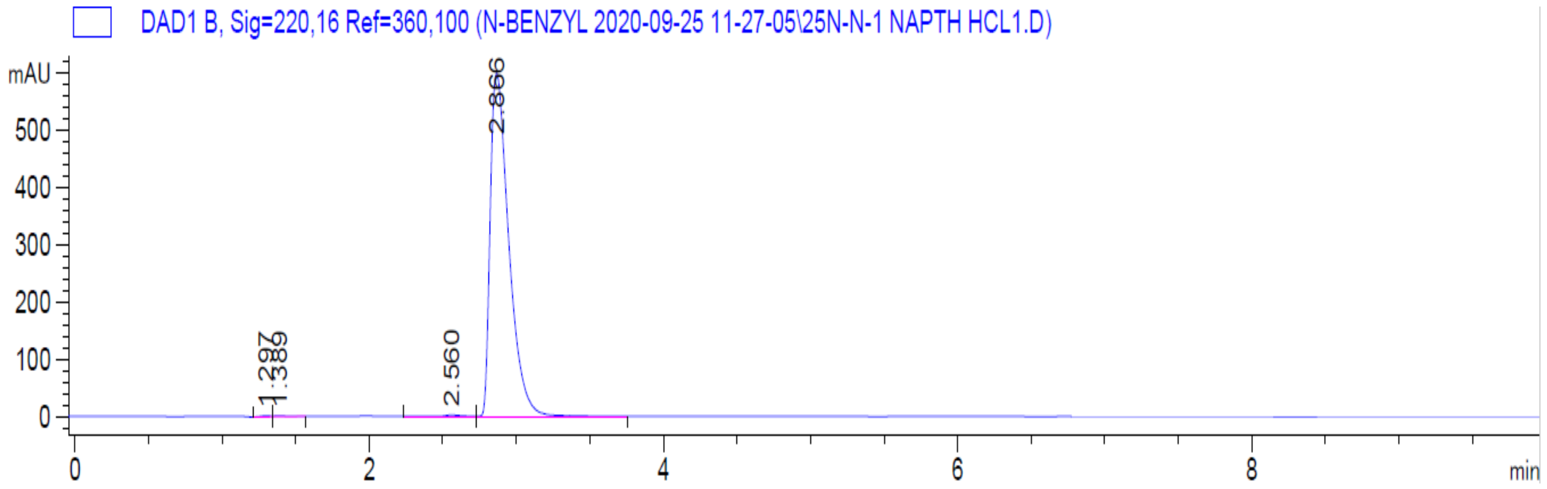

HPLC Trace of 25N-NBPh (17). Purity (Peak area): 98.7%; Wavelength: 220 nm.

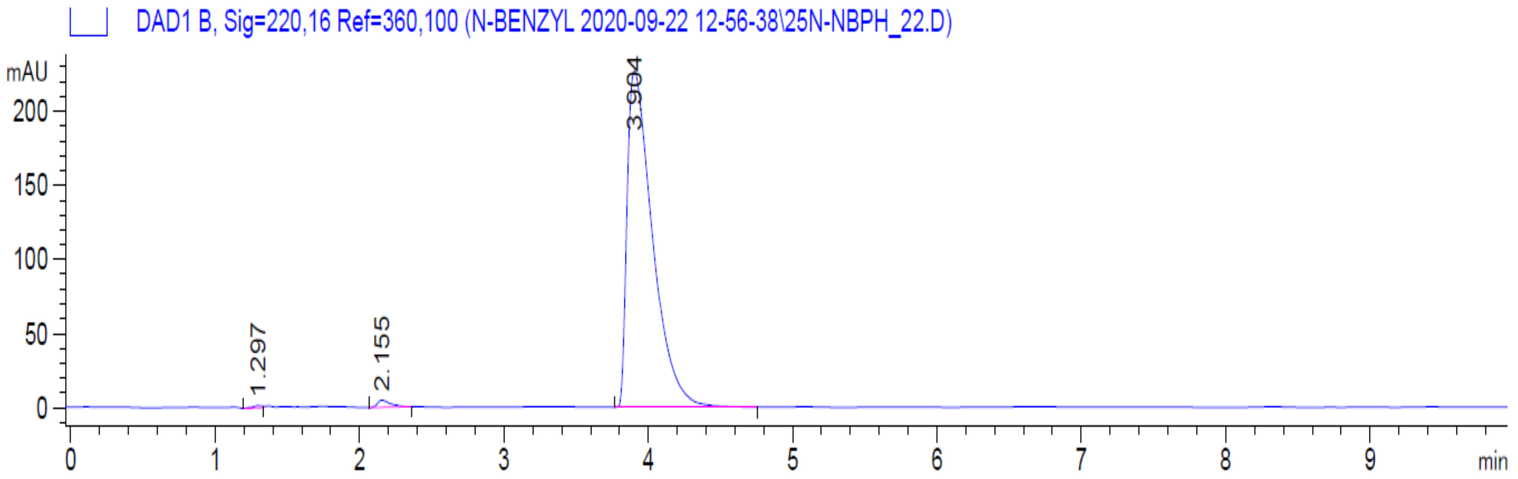

HPLC Trace of 25N-NB-2-OH-3-Me (18). Purity (Peak area): 100%; Wavelength: 220 nm.

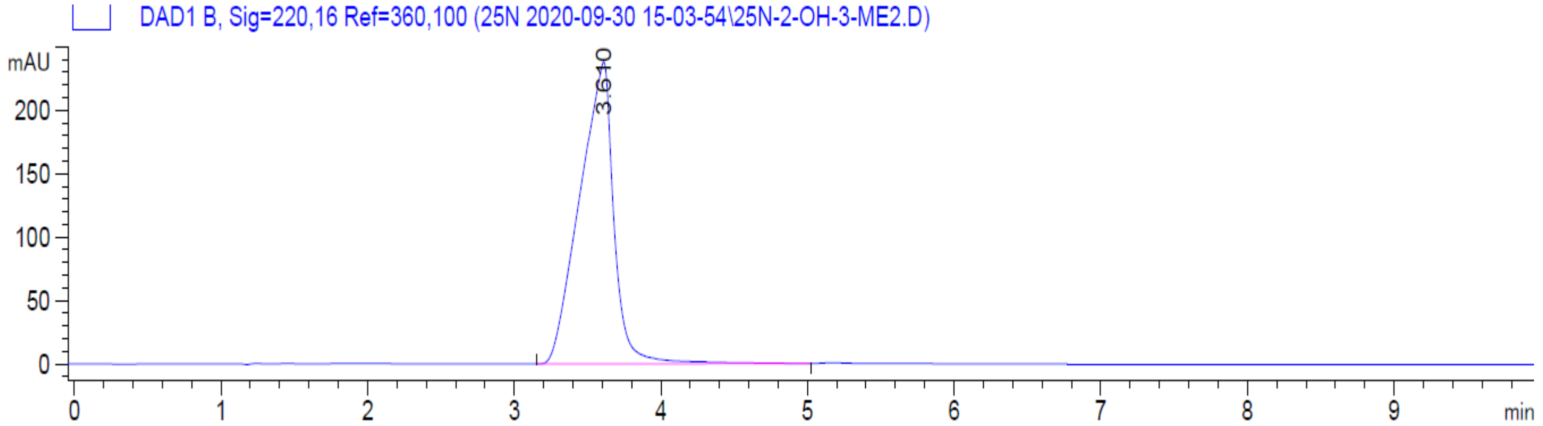

HPLC Trace of 25N-NB-2-MeO-3-F (19). Purity (Peak area): 96.1%; Wavelength: 220 nm.

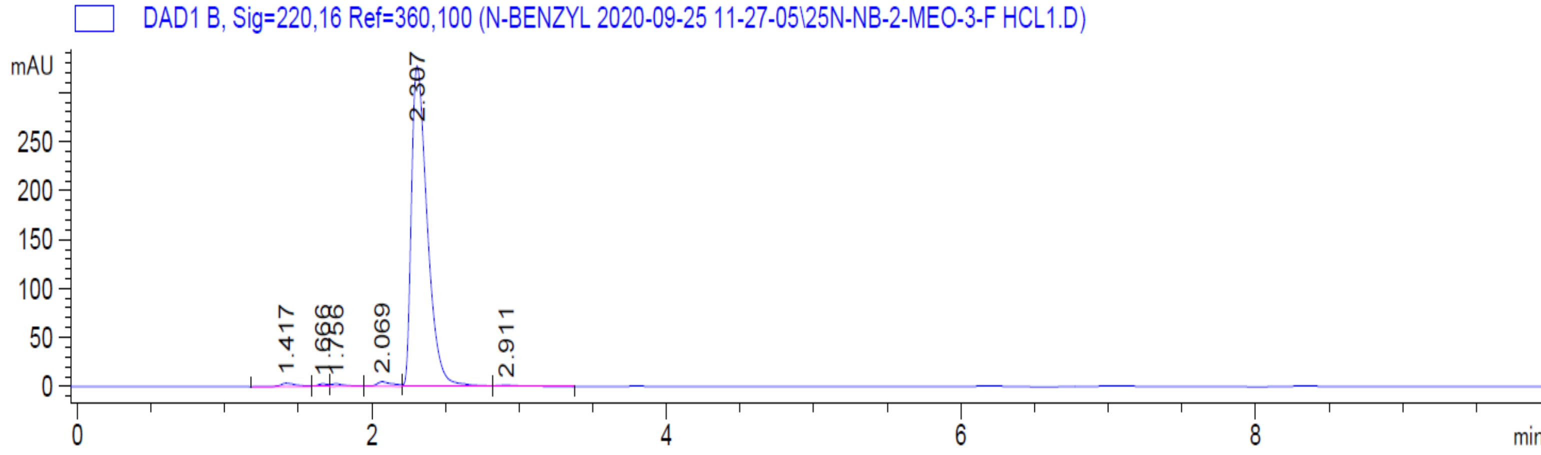

HPLC Trace of 25N-NB-2,5-DiMeO (20). Purity (Peak area): 95.6%; Wavelength: 220 nm.

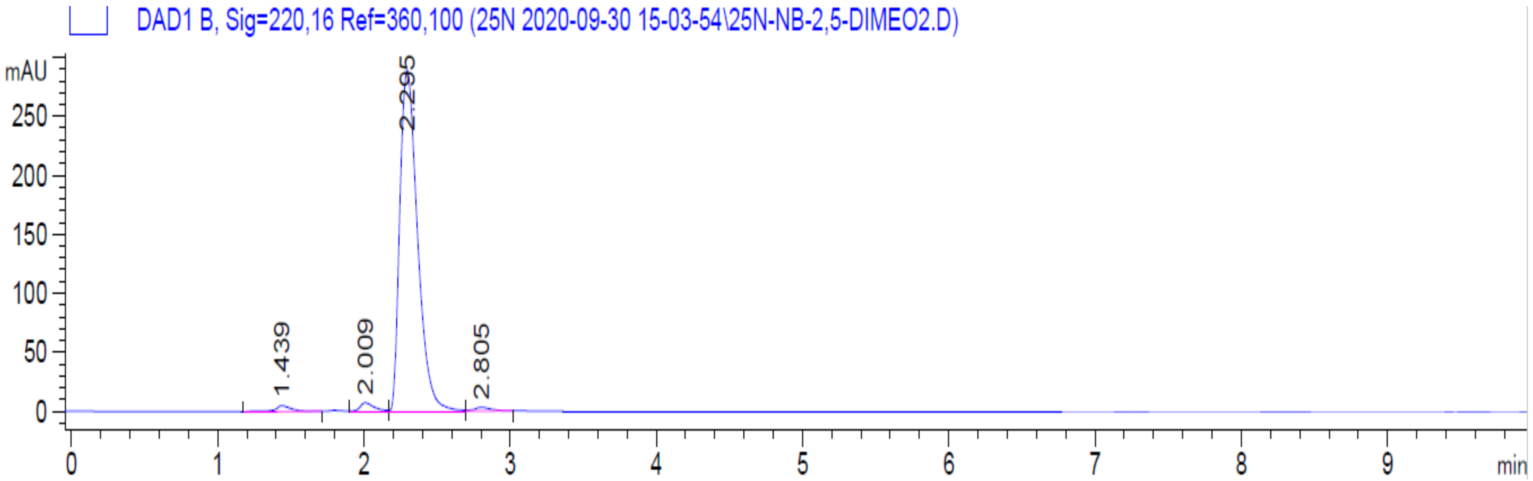

HPLC Trace of 25N-NB-3-OH (21). Purity (Peak area): 99.1%; Wavelength: 220 nm.

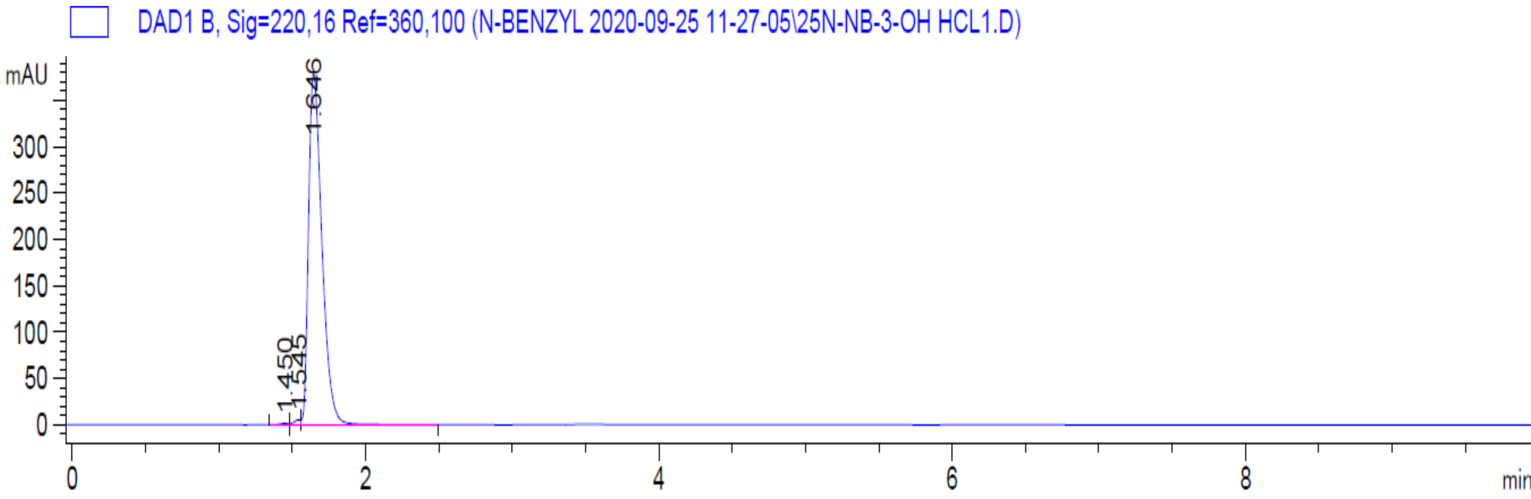

HPLC Trace of 25N-NB-3-Me (22). Purity (Peak area): 98.8%; Wavelength: 220 nm.

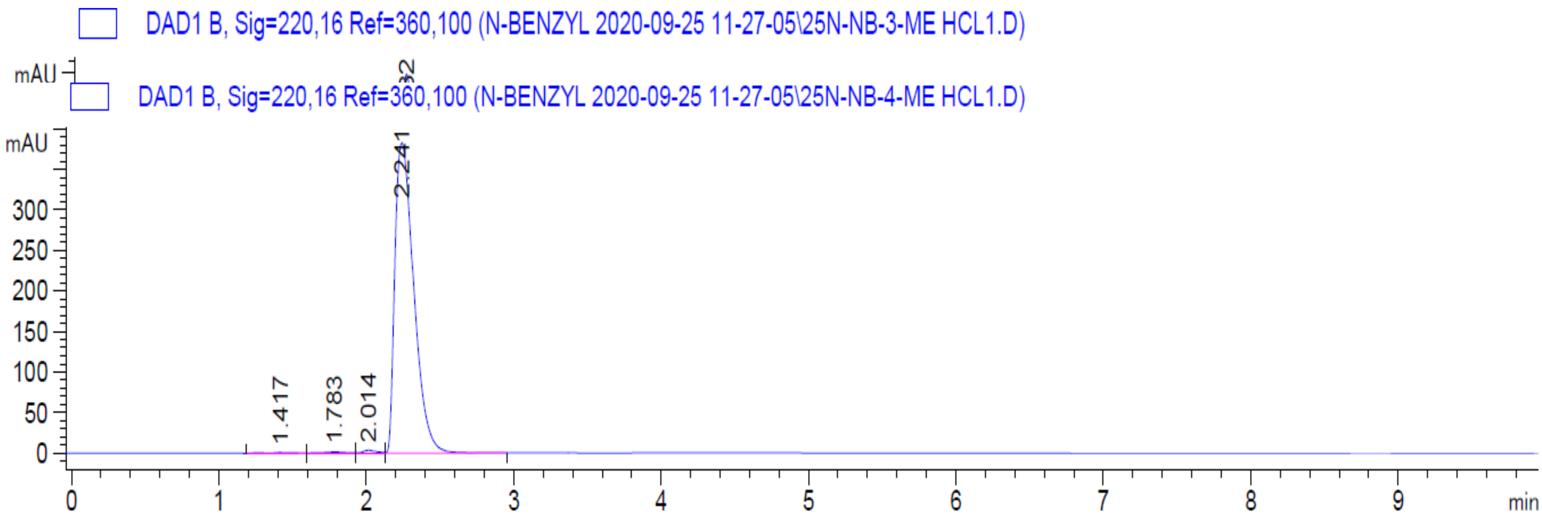

HPLC Trace of 25N-NB-4-Me (23). Purity (Peak area): 98.4%; Wavelength: 220 nm.

HPLC Trace of 25N-NB-3-F (24). Purity (Peak area): 100%; Wavelength: 220 nm.

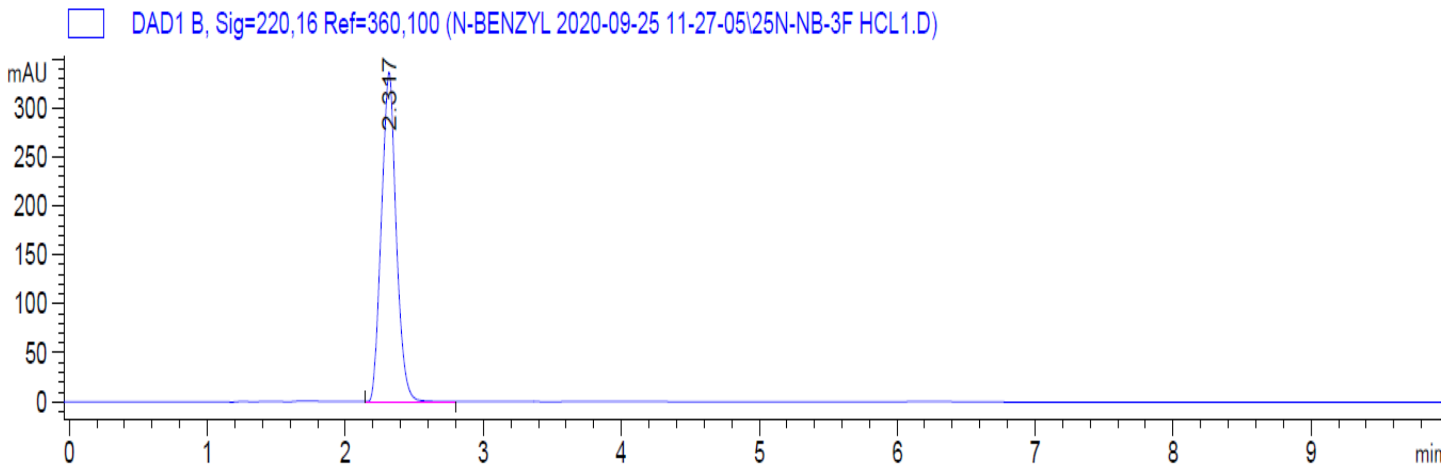

HPLC Trace of 25N-NB-4-F (25). Purity (Peak area): 99.4%; Wavelength: 220 nm.

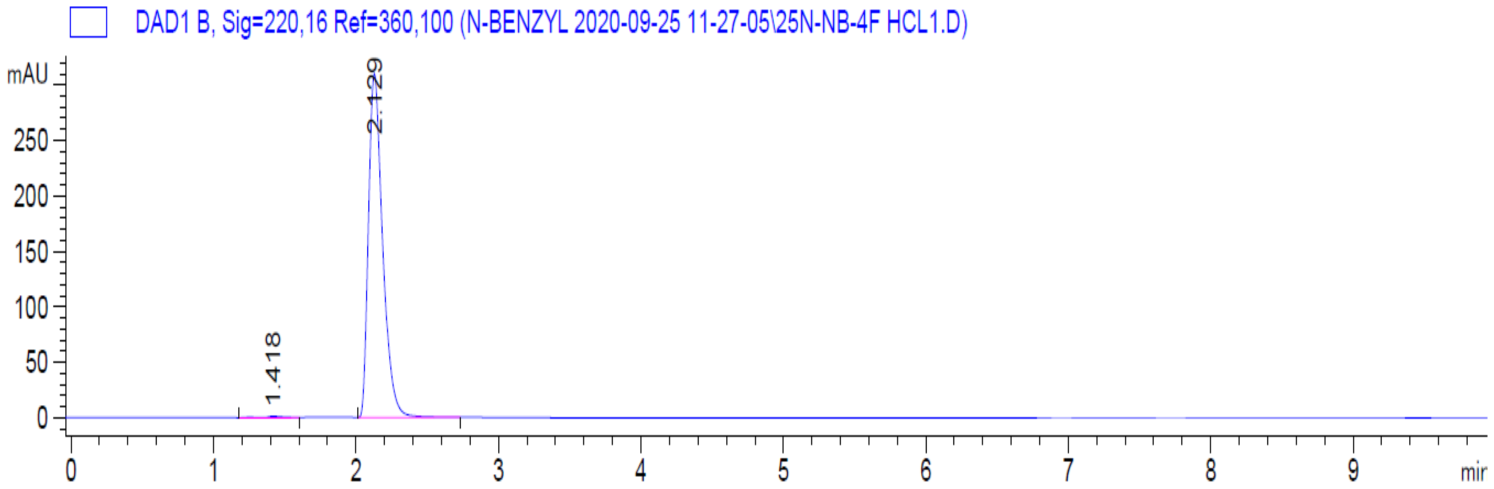

HPLC trace of 25D-N1-Nap (26). Purity (Peak area): 100%; Wavelength: 220 nm.

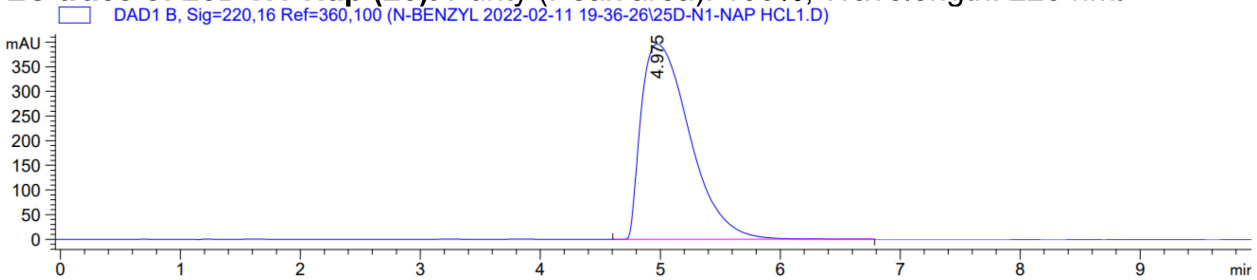

HPLC trace of **25D-NBPh (27).** Purity (Peak area): 98.9%; Wavelength: 220 nm.

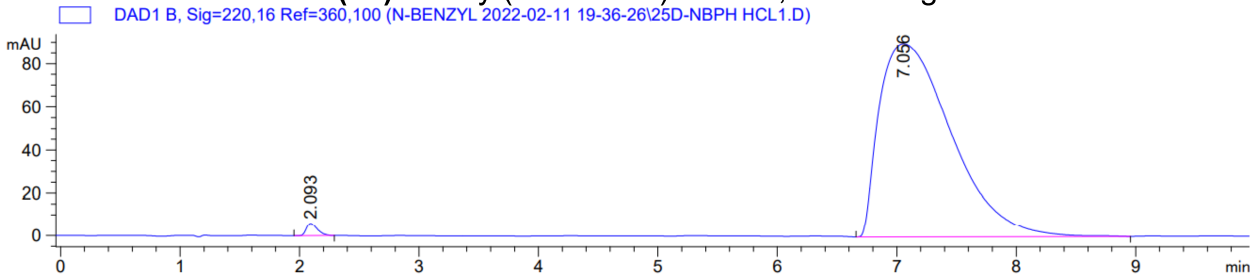

HPLC trace of 25O-N1-Nap (28). Purity (Peak area): 100%; Wavelength: 220 nm.

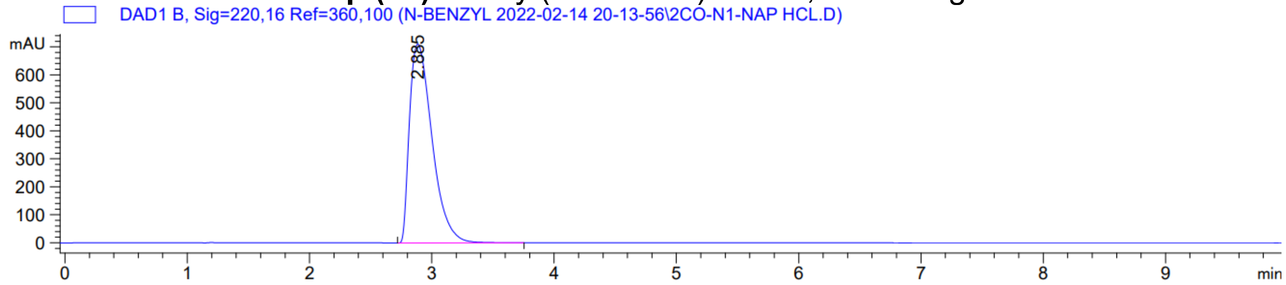

HPLC trace of 2C2-N1-Nap (29). Purity (Peak area): 99.8%; Wavelength: 220 nm.

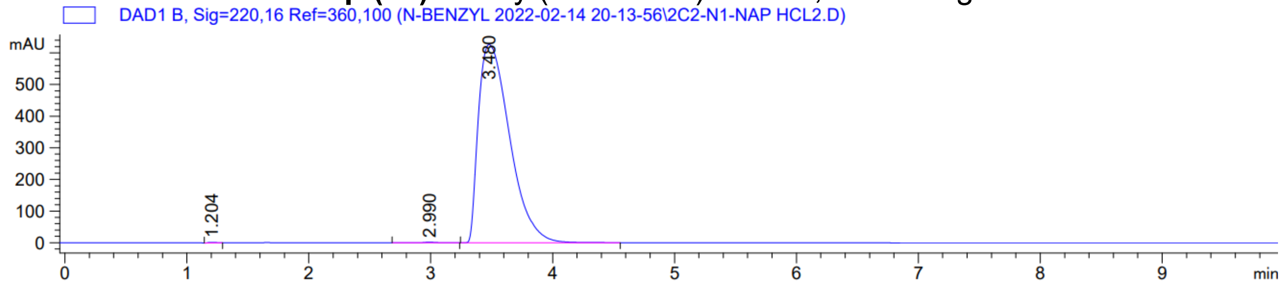

HPLC trace of Psi-DOB-N1-Nap (30). Purity (Peak area): 98.3%; Wavelength: 220 nm.

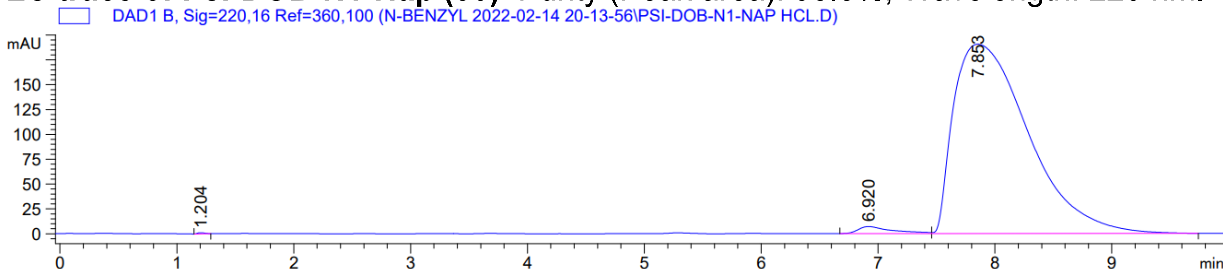

HPLC Trace of 2C2-NBOMe (31). Purity (Peak area): 100%; Wavelength: 220 nm.

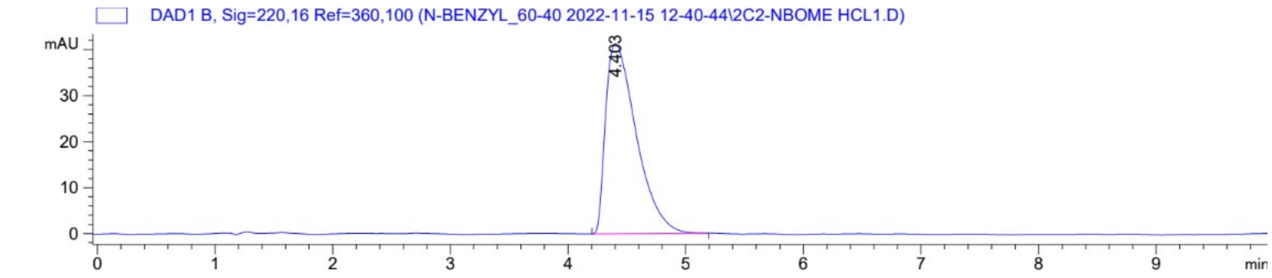

HPLC Trace of 25O-NBOMe (32). Purity (Peak area): 99.5%; Wavelength: 220 nm.

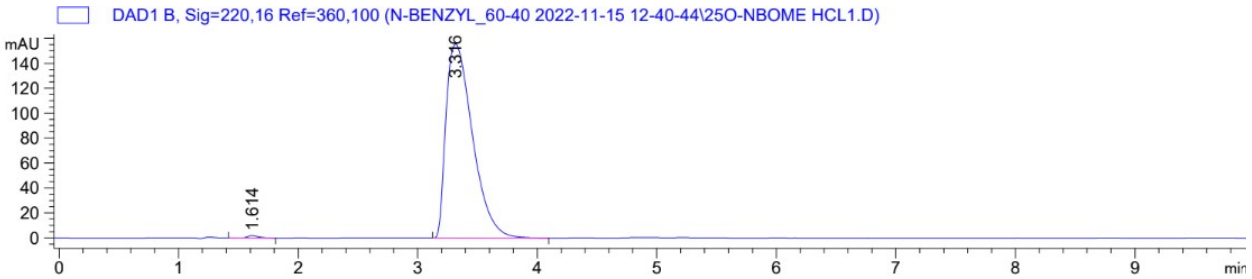

HPLC Trace of 25O-NBcP (33). Purity (Peak area): 100%; Wavelength: 220 nm.

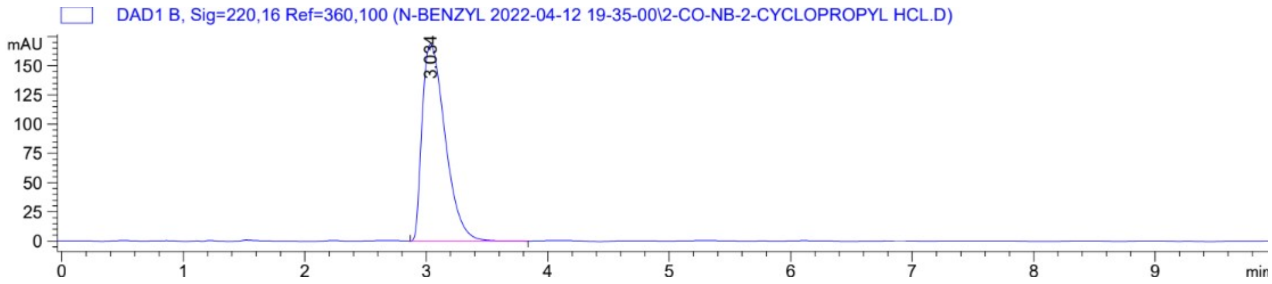

HPLC Trace of 25O-NB-3-I (34). Purity (Peak area): 99.3%; Wavelength: 220 nm.

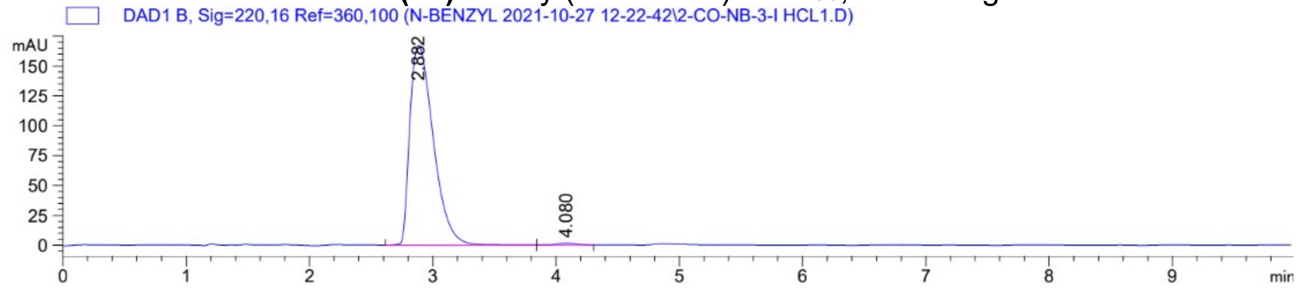

HPLC Trace of 25O-NBPh-10’-OH (35). Purity (Peak area): 97.8%; Wavelength: 220 nm.

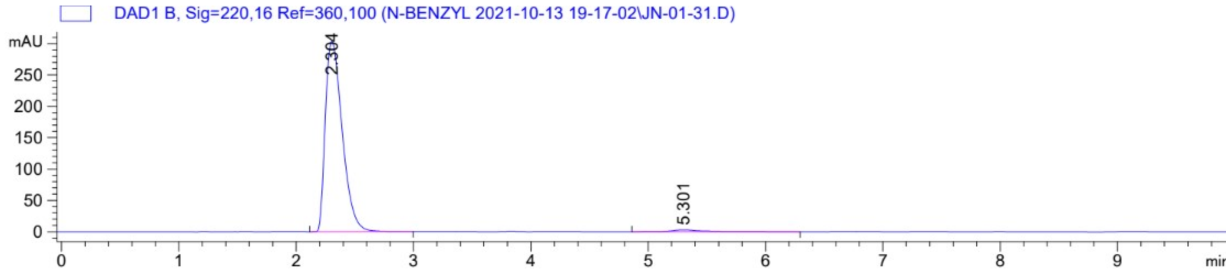

Supplement: Supplementary file 6 — Supplementary Dataset 3 [file 41467_2023_44016_MOESM6_ESM.pdf]
