# Supplementary material for: Identification of 5-HT2A receptor signaling pathways associated with psychedelic potential
Source: Nat Commun. 2023 Dec 15;14:8221. doi: 10.1038/s41467-023-44016-1 (PMC10724237; doi:10.1038/s41467-023-44016-1)
Supplement: Supplementary file 11 — Reporting Summary [file 41467_2023_44016_MOESM11_ESM.pdf]

# nature portfolio

Corresponding author(s): Jason Wallach, Adam Halberstadt, John McCorvy

Last updated by author(s): 11/17/2023

# Reporting Summary

Nature Portfolio wishes to improve the reproducibility of the work that we publish. This form provides structure for consistency and transparency in reporting. For further information on Nature Portfolio policies, see our Editorial Policies and the Editorial Policy Checklist.

## Statistics

For all statistical analyses, confirm that the following items are present in the figure legend, table legend, main text, or Methods section.

| n/a | Confirmed | |
|---|---|---|
| ☐ | ☒ | The exact sample size (*n*) for each experimental group/condition, given as a discrete number and unit of measurement |
| ☐ | ☒ | A statement on whether measurements were taken from distinct samples or whether the same sample was measured repeatedly |
| ☐ | ☒ | The statistical test(s) used AND whether they are one- or two-sided<br>*Only common tests should be described solely by name; describe more complex techniques in the Methods section.* |
| ☐ | ☒ | A description of all covariates tested |
| ☐ | ☒ | A description of any assumptions or corrections, such as tests of normality and adjustment for multiple comparisons |
| ☐ | ☒ | A full description of the statistical parameters including central tendency (e.g. means) or other basic estimates (e.g. regression coefficient) AND variation (e.g. standard deviation) or associated estimates of uncertainty (e.g. confidence intervals) |
| ☐ | ☒ | For null hypothesis testing, the test statistic (e.g. *F*, *t*, *r*) with confidence intervals, effect sizes, degrees of freedom and *P* value noted<br>*Give P values as exact values whenever suitable.* |
| ☒ | ☐ | For Bayesian analysis, information on the choice of priors and Markov chain Monte Carlo settings |
| ☒ | ☐ | For hierarchical and complex designs, identification of the appropriate level for tests and full reporting of outcomes |
| ☐ | ☒ | Estimates of effect sizes (e.g. Cohen's *d*, Pearson's *r*), indicating how they were calculated |

*Our web collection on statistics for biologists contains articles on many of the points above.*

## Software and code

Policy information about availability of computer code

| | |
|---|---|
| Data collection | 1H and 13C NMR spectra data were obtained on a Bruker Avance III with PA BBO 400S1 BBF-H-D-05 Z plus probe (Bruker Corporation, Billerica, MA, USA). HRMS data were obtained on a Thermo Orbitrap Exactive Mass Spectrometer with an Orbitrap mass analyzer. HPLC analyses were performed on an Agilent 1260 Infinity system that includes a 1260 quaternary pump VL, a 1260 ALS autosampler, a 1260 Thermostatted Column Compartment, and a DAD Multiple Wavelength Detector (Agilent Technologies, Santa Clara, CA, USA). In vitro assay data were read using a Mithras LB940 (Berthold) or a PheraStar FSX (BMGLabTech) or a Fluorescence for the FLIPRTETRA (Molecular Devices). Induced fit docking was performed with LIGPREP tool of the Schrödinger (release 2020c). MD simulations used GROMOS96 54a7 and GROMACS v2021.2. HTR data was collected with a coil voltage was low-pass filtered (2 kHz), amplified, and digitized (20-kHz sampling rate) using a Powerlab (model /8SP or 8/35) with LabChart software (ADInstruments, Colorado Springs, CO, USA). |
| Data analysis | Data was plotted and analysis was performed in Graphpad Prism 9. Chromatograms were analyzed using the Agilent ChemStation Software (Agilent Technologies, Santa Clara, CA, USA). Poses generated in the IFD runs were analyzed with PyMol visualization application (The PyMOL Molecular Graphics System, Version 2.0 Schrödinger, LLC). MD plots Plots were made in Grace v5.1.25, Seaborn and Matplotlib, GROMACS's mdrun command, and visualized with Schrödinger's Maestro . |

For manuscripts utilizing custom algorithms or software that are central to the research but not yet described in published literature, software must be made available to editors and reviewers. We strongly encourage code deposition in a community repository (e.g. GitHub). See the Nature Portfolio guidelines for submitting code & software for further information.

## Data

Policy information about availability of data

All manuscripts must include a data availability statement. This statement should provide the following information, where applicable:

- Accession codes, unique identifiers, or web links for publicly available datasets
- A description of any restrictions on data availability
- For clinical datasets or third party data, please ensure that the statement adheres to our policy

All data generated in this study are included in this article, Supplementary Information, and in Supplementary Datasets. Source data are provided with this paper.
PDB deposited structural data are as follows:
6WHA, https://doi.org/10.2210/pdb6WHA/pdb, 5-HT2A cryo-EM structure in complex with 25CN-NBOH
6WGT, https://doi.org/10.2210/pdb6WGT/pdb, 5-HT2A crystal structure in complex with LSD
6A94, https://doi.org/10.2210/pdb6A94/pdb, 5-HT2A crystal structure in complex with zotepine
6A93, https://doi.org/10.2210/pdb6A93/pdb, 5-HT2A crystal structure in complex with risperidone
6WH4, https://doi.org/10.2210/pdb6WH4/pdb, 5-HT2A crystal structure in complex with methiothepin
Custom code is available at github: https://github.com/jruhym/jNotebooksForMD/blob/trunk/pocket-ligandInteractionTimeSeriesHeatmap.ipynb.

## Human research participants

Policy information about studies involving human research participants and Sex and Gender in Research.

| | |
|---|---|
| Reporting on sex and gender | N/A |
| Population characteristics | N/A |
| Recruitment | N/A |
| Ethics oversight | N/A |

Note that full information on the approval of the study protocol must also be provided in the manuscript.

# Field-specific reporting

Please select the one below that is the best fit for your research. If you are not sure, read the appropriate sections before making your selection.

☒ Life sciences ☐ Behavioural & social sciences ☐ Ecological, evolutionary & environmental sciences

For a reference copy of the document with all sections, see nature.com/documents/nr-reporting-summary-flat.pdf

# Life sciences study design

All studies must disclose on these points even when the disclosure is negative.

| | |
|---|---|
| Sample size | In vitro experiments were performed in biological triplicate forming independent replicates (technical duplicates or triplicate per biological replicate) which is standard for the field. HTR counts were analyzed using one-way or two-way ANOVAs; Dunnett's test or Tukey's test was used for post hoc comparisons. Significance was demonstrated by surpassing an $\alpha$ level of 0.05. |
| Data exclusions | No data were excluded from the study. |
| Replication | Data were replicated using technical and independent replicates. See figures and table legends for details. |
| Randomization | For the behavioral studies, mice were randomly assigned across treatments. |
| Blinding | In vitro and the HTR experiments done on Male C57BL/6J mice (6–8 weeks old) were performed by blinding the experimenter to drug identity. |

# Reporting for specific materials, systems and methods

We require information from authors about some types of materials, experimental systems and methods used in many studies. Here, indicate whether each material, system or method listed is relevant to your study. If you are not sure if a list item applies to your research, read the appropriate section before selecting a response.

## Materials & experimental systems

| n/a | Involved in the study |
| --- | --- |
| ☒ | ☐ Antibodies |
| ☐ | ☒ Eukaryotic cell lines |
| ☒ | ☐ Palaeontology and archaeology |
| ☐ | ☒ Animals and other organisms |
| ☒ | ☐ Clinical data |
| ☒ | ☐ Dual use research of concern |

## Methods

| n/a | Involved in the study |
| --- | --- |
| ☒ | ☐ ChIP-seq |
| ☒ | ☐ Flow cytometry |
| ☒ | ☐ MRI-based neuroimaging |

# Eukaryotic cell lines

Policy information about cell lines and Sex and Gender in Research

| | |
| --- | --- |
| Cell line source(s) | HEK293T (CRL-11268) was purchased from American Type Culture Collection (Rockville, MD, USA). Flp-In 293 cells were purchased from Invitrogen. |
| Authentication | Cells were authenticated by ATCC upon purchase by STR markers. |
| Mycoplasma contamination | Cells were tested and certified to be mycoplasma-free. |
| Commonly misidentified lines (See ICLAC register) | No commonly misidentified cell lines were used in the study. |

# Animals and other research organisms

Policy information about studies involving animals; ARRIVE guidelines recommended for reporting animal research, and Sex and Gender in Research

| | |
| --- | --- |
| Laboratory animals | Male C57BL/6J mice (6–8 weeks old) from Jackson Labs (Bar Harbor, ME, USA) were used for the behavioral experiments. The mice were housed on a reversed light-dark cycle (lights on at 1900 h, off at 0700 h,) in an AALAC-approved vivarium at the University of California San Diego. Mice were housed up to four per cage in a climate-controlled room and with food and water provided ad libitum except during behavioral testing. Testing was performed between 1000 and 1800 h (during the dark phase of the light-dark cycle). |
| Wild animals | Study did not involved wild animals. |
| Reporting on sex | Study reports sex of animals used. |
| Field-collected samples | No field collected samples were used in this study. |
| Ethics oversight | The studies were conducted in accordance with National Institutes Health (NIH) guidelines and were approved by the University of California San Diego Institutional Animal Care and Use Committee (Protocol #S17044). |

Note that full information on the approval of the study protocol must also be provided in the manuscript.
